# Discovery of Genomic Characteristics and Selection Signatures in Korean Indigenous Goats Through Comparison of 10 Goat Breeds

**DOI:** 10.3389/fgene.2019.00699

**Published:** 2019-08-08

**Authors:** Jae-Yoon Kim, Seongmun Jeong, Kyoung Hyoun Kim, Won-Jun Lim, Ho-Yeon Lee, Namshin Kim

**Affiliations:** ^1^Genome Editing Research Center, Korea Research Institute of Bioscience and Biotechnology (KRIBB), Daejeon, South Korea; ^2^Department of Bioinformatics, KRIBB School of Bioscience, University of Science and Technology (UST), Daejeon, South Korea

**Keywords:** Korean indigenous goats, selection signature, genomic characteristics, population genetics, *Capra hircus* (goat)

## Abstract

Indigenous breeds develop their own genomic characteristics by adapting to local environments or cultures over long periods of time. Most of them are not particularly productive in commercial terms, but they have abilities to survive in harsh environments or tolerate to specific diseases. Their adaptive characteristics play an important role as genetic materials for improving commercial breeds. As a step toward this goal, we analyzed the genome of Korean indigenous goats within 10 goat breeds. We collected 136 goat individuals by sequencing 46 new goats and employing 90 publicly available goats. Our whole-genome data was comprised of three indigenous breeds (Korean indigenous goat, Iranian indigenous goat, and Moroccan indigenous goat; *n* = 29, 18, 20), six commercial breeds (Saanen, Boer, Anglo-Nubian, British Alpine, Alpine, and Korean crossbred; *n* = 16, 11, 5, 5, 2, 13), and their ancestral species (*Capra aegagrus*; *n* = 17). We identified that the Iranian indigenous goat and the Moroccan indigenous goat have relatively similar genomic characteristics within a large category of genomic diversity but found that the Korean indigenous goat has unique genomic characteristics distinguished from the other nine breeds. Through population analysis, we confirmed that these characteristics have resulted from a near-isolated environment with strong genetic drift. The Korean indigenous goat experienced a severe genetic bottleneck upon entering the Korean Peninsula about 2,000 years ago, and has subsequently rarely experienced genetic interactions with other goat breeds. From selection analysis and gene-set enrichment analysis, we revealed selection signals for *Salmonella* infection and cardiomyopathy in the genome of the Korean indigenous goat. These adaptive characteristics were further identified with genomic-based evidence. We uncovered genomic regions of selective sweeps in the LBP and BPI genes (*Salmonella* infection) and the TTN and ITGB6 genes (cardiomyopathy), among several candidate genes. Our research presents unique genomic characteristics and distinctive selection signals of the Korean indigenous goat based on the extensive comparison. Although the adaptive traits require further validation through biological experiments, our findings are expected to provide a direction for future biodiversity conservation strategies and to contribute another option to genomic-based breeding programmes for improving the viability of *Capra hircus*.

## Introduction

Goats (*Capra hircus*) are one of the oldest domesticated animals, originating from the wild bezoar goat (*Capra aegagrus*) near the Fertile Crescent of western Asia (Iranian region) (Zeder and Hesse, 2000; Zeder, 2005). Their domestication occurred around the Neolithic period, approximately 10,000 years ago, when human lifestyles moved from hunting to farming (Li and Zhang, 2009). At this time, the goats started supplying milk, meat, fur, and hair to humans in a stable manner, and gradually began to establish a close relationship economically, culturally, and religiously with human civilization (Naderi et al., 2008). As their contribution to humanity increased, goats spread rapidly to the rest of the world following human migration and trade routes (Taberlet et al., 2008; Tresset and Vigne, 2011), and they now comprise more than 1,006 million individuals and over 300 breeds, including commercial and indigenous breeds (http://faostat3.fao.org/browse/Q/QA/E).

Indigenous breeds have locality-specific characteristics, with considerable regional diversity. During the geographical expansion, goats have spread to a wide range of environments spanning hot to cold climates, humid to dry climates, and tropical rainforests to hypoxic high-altitude regions. They have successfully adapted to these diverse environments (Nomura et al., 2013), and have developed distinctive characteristics in their local environments. For instance, in desert areas, one of the Moroccan indigenous goat breeds (the Draa population) has been reported to have acquired the characteristics of frequently gasping to regulate body temperature (Benjelloun et al., 2015). In the highlands, Tibetan indigenous goats have been reported to have developed an oxygen-sensing ability for adapting to hypoxia in high altitudes (Song et al., 2016; Wang et al., 2016). Additionally, Ugandan indigenous goats have been reported to have enhanced their immune competence in order to resist infection by parasites in Africa’s tropical environment (Onzima et al., 2018). As useful information, these adaptive characteristics have provided an important base to various breeding programs aimed at improving goat breeds (Giovambattista et al., 2001; Babayan, 2016). For example, Chinese indigenous goats of the Shandong Province, with adaptive characteristics to the humid climate, were used to develop Laoshan dairy goats through selective crossbreeding with Saanen dairy goats (Porter et al., 2016). Due to this breeding effort, the Laoshan dairy goats have acquired both humid climate adaptability (Chinese indigenous goats) and high dairy productivity (Saanen goats) (Li et al., 2008). Also, Indonesian indigenous goats (Katjang goats), which are adapted to the equatorial climate, were utilized to develop Peranakan Etawah goats through crossbreeding with Indian indigenous goats (Jamunapari goats) (Porter et al., 2016). The Peranakan Etawah goats, thus, have shown both equatorial climate adaptability (Indonesian indigenous goats) and high dairy and meat productivity (Indian indigenous goats) (Sodiq, 2004).

**Figure 1:**
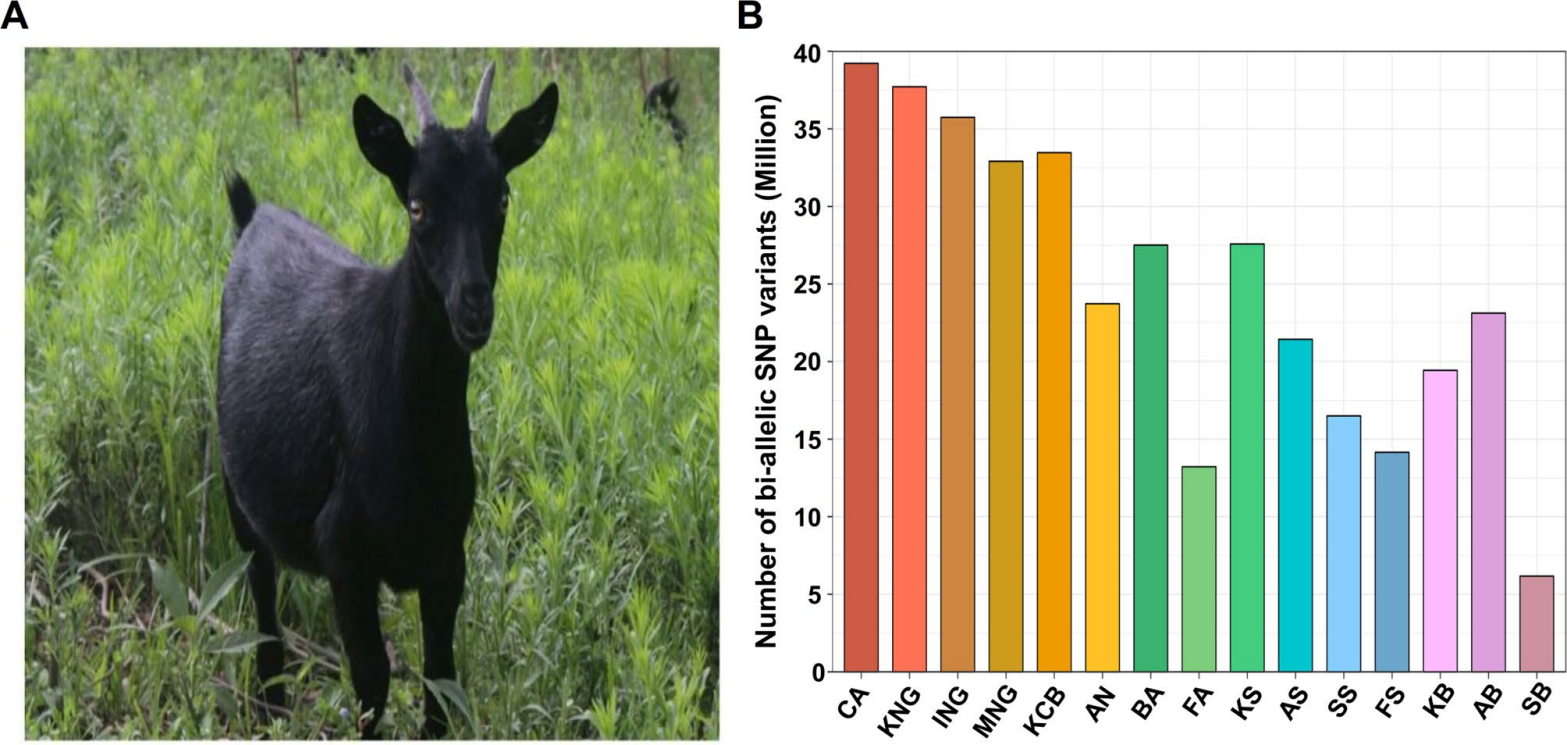
Appearance of Korean indigenous goats (KNG) and distribution of bi-allelic single nucleotide polymorphism (SNP) variants. **(A)** Appearance of KNG with black coat color. **(B)** Distribution of bi-allelic SNPs for 15 goat populations. The *y-axis* represents the number of bi-allelic SNPs detected in each goat population with respect to the reference genome CHIR 2.0. The abbreviation CA means *C. aegagrus* (See **Data sheet 1: Table S5** for a detailed summary of each breed).

Korean indigenous goats (KNG) are the only indigenous goat breed inhabiting the Korean Peninsula. The KNG is characterized by black fur (**Figure 1A**) and is registered with the Food and Agriculture Organization of the United Nations as a single breed (Kim et al., 2011). The origin of the KNG is unclear, but according to previous reports and historical documents, it is estimated that they moved into the Korean Peninsula at least 2,000 years ago after passing through the Northern Mongolia or the Southern coast of China (Tavakolian, 2000; Zeder and Hesse, 2000). Since the influx, the KNG has developed its own unique characteristics while adapting to the peninsula environment for a long time (Rischkowsky and Pilling, 2007). Some of their unique characteristics have been reported through several previous studies. In terms of genetic diversity, Odahara et al. reported that the KNG has not undergone genetic interactions with imported breeds (Odahara et al., 2006). With respect to disease resistance, Jang revealed that *Salmonella* species was not isolated from the feces of either 49 KNG with symptoms of diarrhea or 620 healthy KNG (Jang, 1995). Kang, and Lee et al. also revealed that the KNG lacks *Salmonella* infection due to their excellent antibody production and innate resistance factors (Kang and Tak, 1996; Lee et al., 2000). In addition, Lee et al. reported that the KNG has an adaptive characteristic associated with lumbar paralysis resistance when compared with their crossbreed, Korean crossbred goats (KCB) (Lee et al., 2016). Although the KNG has not been investigated in as much depth as other breeds, these studies have suggested that KNG possesses unique and useful characteristics as an indigenous breed, and also have raised the need for additional research to further reveal their characteristics.

In recent times, the KNG is gradually losing its unique characteristics. After an agreement with the World Trade Organization in the 1990s, various commercial breeds have been introduced into Korea in earnest (Son, 1999). Since then, the KNG with relatively low commercial productivity has been extensively crossed with imported breeds such as Boer or Saanen, and more recently the KCB, a crossbred of KNG, has even been developed by these hybridizations. However, the genomic characteristics and biodiversity of the KNG have not been extensively investigated. Only one study has reported on the KNG’s characteristics at the whole-genome level (Lee et al., 2016). This study compared KNG with only their hybrid KCB, and thus had limitations in identifying the various genomic and adaptive characteristics of KNG. Also, other studies on the KNG’s *Salmonella* infection and isolated environmental characteristics require further research based on the genome (Jang, 1995; Kang and Tak, 1996; Lee et al., 2000; Odahara et al., 2006).

In this paper, we conducted a comparative genomic study to reveal the genomic and adaptive characteristics of KNG. For extensive comparison, we analysed whole-genome variations of 10 goat breeds comprising three indigenous breeds (KNG, Iranian indigenous goats, and Moroccan indigenous goats), six commercial breeds (KCB, Saanen, Alpine, British-Alpine, Boer, and Anglo-Nubian), and an ancestral species (*C. aegagrus*). We not only identified the characteristics of KNG, but also established for the first time the genetic relationships between the 10 goat breeds, with the criteria of the ancestral species and Iranian indigenous goats. The aims of our study were: to unravel the genomic characteristics of KNG in the 10 goat breeds; to present genomic evidence that KNG has rarely experienced interactions with other breeds; and to elucidate selection signals that KNG has adapted to their environment.

## Material and Methods

### Sample Preparation and Re-Sequencing

Blood samples from 46 goats were obtained from the Animal Genetic Resources Station, National Institute of Animal Science, Rural Development Administration in Korea. The blood samples comprised 14 Korean indigenous goats, 10 Korean Saanen, and 4 Korean Boer, which live in Korea; and 5 Anglo-Nubian, 5 British Alpine, 6 Australian Boer, and 2 Australian Saanen, which live in Australia. DNA was isolated according to the manufacturer’s protocol using the G-DEXTMIIb Genome DNA Extraction Kit (iNtRoN Biotechnology, Korea), and 3 µg of this genomic DNA was randomly sheared to have an insert size of 300bp using the Covaris System. The fragments of sheared DNA were amplified with the TruSeq DNA Sample Prep Kit (Illumina, USA) and were then sequenced as paired-end reads with approximately 10-fold coverage using the Illumina HiSeq 2000 platform with the TruSeq SBS Kit v3-HS (Illumina). These 46 goat sequences with paired-end reads were deposited in the European Nucleotide Archive under the accession number PRJEB25062. Additionally, we used 90 publicly-available goat genomes comprising 15 Korean indigenous goats, 13 Korean crossbred, 20 Moroccan indigenous goats, 18 Iranian indigenous goats, 17 C*. aegagrus*, two French Saanen, two French Alpine, two Swiss Saanen, and one Swiss Boer. As for the Australia Saanen, the French Saanen, the Swiss Saanen, and the Swiss Boer, we mentioned only their overall trends, because they could have a sampling bias due to a small number of samples. We mainly used the integrated Saanen population (*n* = 16) and Boer population (*n* = 11). Additional information of these breeds about sample sizes and bio-project IDs are summarized in **Data sheet 1: Table S1**, and brief sampling information is provided in **Data sheet 1: Note S1.1**.

### Data Processing and Variant Calling

We conducted a per-base sequence quality check for the 136 goat samples using FastQC (Andrews, 2010) and controlled sequences with low quality using NGSQCToolkit (Patel and Jain, 2012). The paired-end sequence reads of each of the 136 samples were then mapped against the reference goat genome, the genome of China’s Yunnan black goat 2.0 version (CHIR v2.0), through BWA (Li and Durbin, 2010). The mapped BAM files were sorted into the genomic coordinates of their reference genome using the Picard software’s “AddOrReplaceReadGroup” (http://broadinstitute.github.io/picard), and potential PCR duplicates were removed using the “MarkDuplicates” option of the software (**Data sheet 1: Tables S2 and S3**). Then, the “RealignerTargetCreator” and “IndelRealigner” of the Genome Analysis Toolkit v3.7 (GATK) (Van der Auwera et al., 2013) were used to correct misalignments resulting from INDELs that may exist in the mapped reads. Following this preparation, we generated gVCF files for each of the 136 samples, which were called to all base sites of the reference genome using the GATK’s “HaplotypeCaller,” combined these gVCF files as one gVCF file through the GATK’s “CombineGVCFs,” and converted the file into a VCF file using the GATK’s “GenotypeVCFs.” To exclude as many false positively called variants as possible, the arguments “Variant Filtration” and “Select Variants” of the GATK were adopted with the following options: 1) Phred-scaled quality score (QUAL) < 35.0; 2) Quality score by depth (QD) < 5.0; 3) Genotype quality score (GQ) < 15.0; 4) Mapping quality score (MQ) < 30.0; 5) Phred-scaled *P*-value score of Fisher’s exact test for identifying strand bias (FS) > 30.0; 6) Depth of coverage across all samples (DP) < 7; 7) Rank sum test for mapping quality of reference and alternative reads (MQRankSum) < −2.0; and 8) Ranks sum test on the bias of the relative positions of the reference alleles and the alternative alleles in the read (ReadPosRankSum) < −2.0. We additionally filtered variants with genotype missing rates of >50% in order to use relatively common variants. Single nucleotide polymorphism (SNP) and INDEL variants were then separated from the VCF, and bi-allele-type SNPs were extracted (**Figure 1B** and **Data sheet 1: Table S4**). For loci with three or more alleles, we maintained only the allele with the highest allele frequency as the only alternative allele representing the corresponding locus. Lastly, haplotype phasing and imputation were conducted using BEAGLE v4.18 (Browning and Browning, 2007). This variant calling process was also performed for each breed to obtain breed-specific SNPs (**Data sheet 1: Table S5**). The functional effects of these SNPs on the genomic and protein regions were annotated by SnpEff (Cingolani et al., 2012) (**Data sheet 1: Table S6**). Since the gene set of the reference genome CHIR v2.0 has not been fully developed, we used a gene set that mapped the CHIR v1.0 gene set to the CHIR v2.0 reference genome using GMAP (Wu and Watanabe, 2005).

### General Genomic Characteristics

Nucleotide diversity (π) was calculated by sliding 50 Kb with a window size of 100 Kb using VCFtools v4.1 (Danecek et al., 2011). Inbreeding coefficient (*F*) was calculated using the same software. The individual’s *F* value was obtained by averaging the deviations of observed heterozygous genotype frequency (Ho) from expected heterozygous genotype frequency under random mating (He) (*F* = 1 – Ho/He) for all loci, and the breed’s *F* value was derived by averaging these *F* values of all individuals belonging to each breed. Linkage disequilibrium (LD) was measured as *r* ^2^ statistic suggested by Hill and Robertson (Hill and Robertson, 1968), and computed using all bi-allelic SNPs through PopLDdecay v3.2 (https://github.com/BGI-shenzhen/PopLDdecay). Then, the averages of pairwise LDs for all SNPs within 30 Kb, 50 Kb, 100 Kb, and 500 Kb regions were calculated. A summary of these three measurements, π, *F*, and LD, is provided in **Data sheet 1: Table S7**, and the average degree of collapse of the LD up to 500 Kb is displayed in **Data sheet 1: Figures S1A–D**.

### Population Differentiation and Genetic Structure

**Figure 2:**
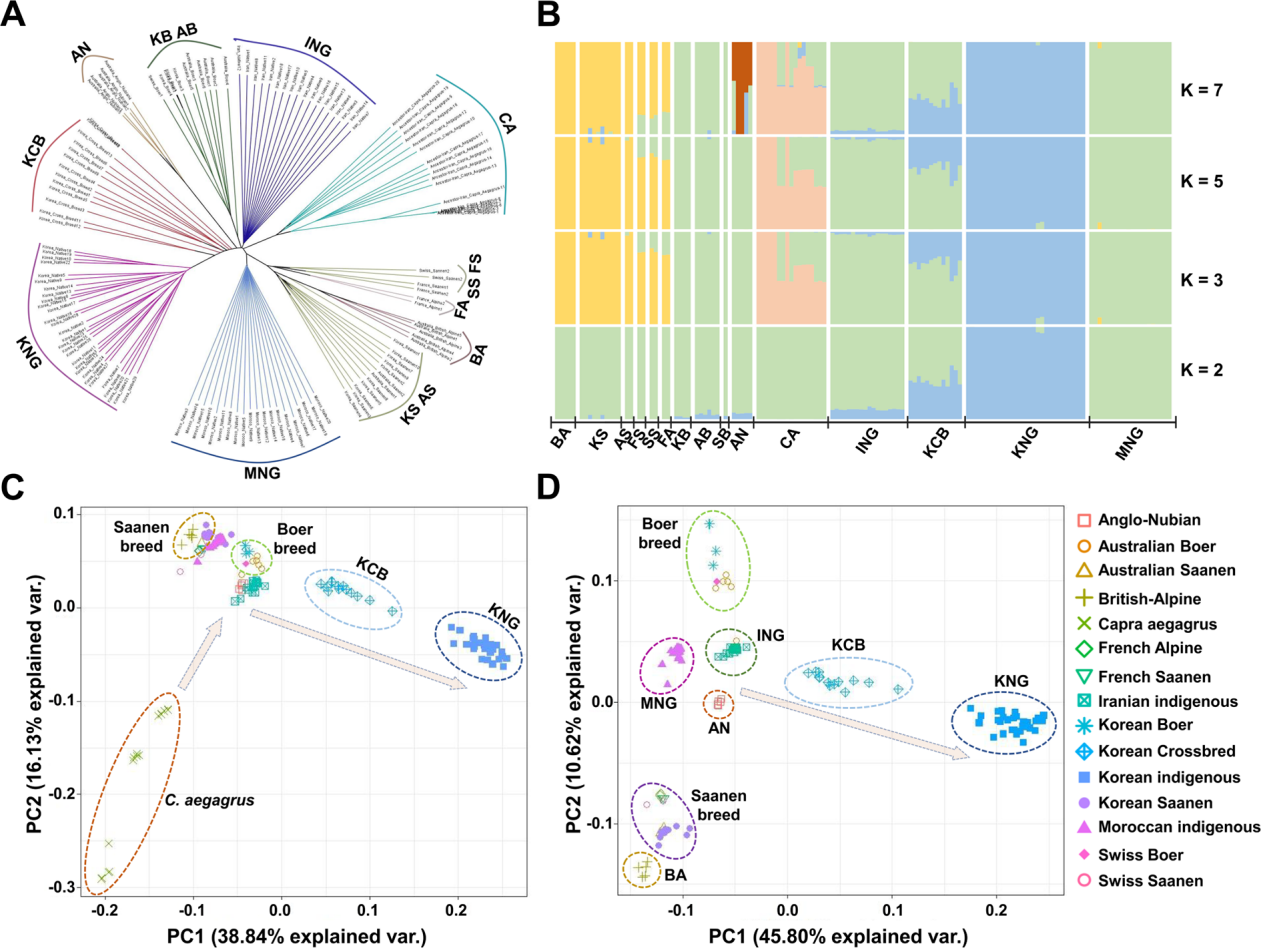
Genomic structure and relationship of 15 goat populations. **(A)** Bio-neighbor-joining tree for the relationship computed using the identical-by-state (IBS). **(B)** Genomic structure computed based on the maximum likelihood-based clustering algorithm. **(C)**, **(D)** Principal component analysis for the genomic differentiation including or excluding *C. aegagrus*, respectively.

Fixation index value (Fst) (Weir and Cockerham, 1984) was calculated for 15 goat populations by sliding 50 Kb with a window size of 100 Kb using the VCFtools (**Data sheet 1: Table S8**). A phylogenetic tree was computed based on the identity-by-state matrix (**Data sheet 1: Figure S2**) which was calculated from all 136 goat samples using Plink v1.90b (Purcell et al., 2007) and reconstructed using the BIO-neighbor-joining algorithm (Gascuel, 1997) which is an improved version of the neighbor-joining algorithm. Then, the tree was visualized using FigTree v1.4.3 (http://tree.bio.ed.ac.uk/software/figtree/) (**Figure 2A**). A structure analysis was performed using FAST-STRUCTURE v1.0 (Raj et al., 2014), which is based on a variational Bayesian framework (**Figure 2B** and **Data sheet 1: Figure S3**). The number of genetic clusters (K) was estimated from 2 to 10, and each genetic cluster was calculated *via* cross-validation 10 times with the 1e-7 convergence criterion using the simple prior model. In our case, with the high population structure, the simple prior model was appropriate. A principal component analysis (PCA) was performed by the singular value decomposition of the relationship matrix derived from the Kimura two-parameter model (Kimura, 1980). The PCA plots were displayed using principal components 1, 2, and 3, and the scree plots were presented with their eigenvectors and explanatory powers (**Figures 2C, D** and **Data sheet 1: Figures S4A–D**).

### Inference of Gene Flow and Demographic History

**Figure 3:**
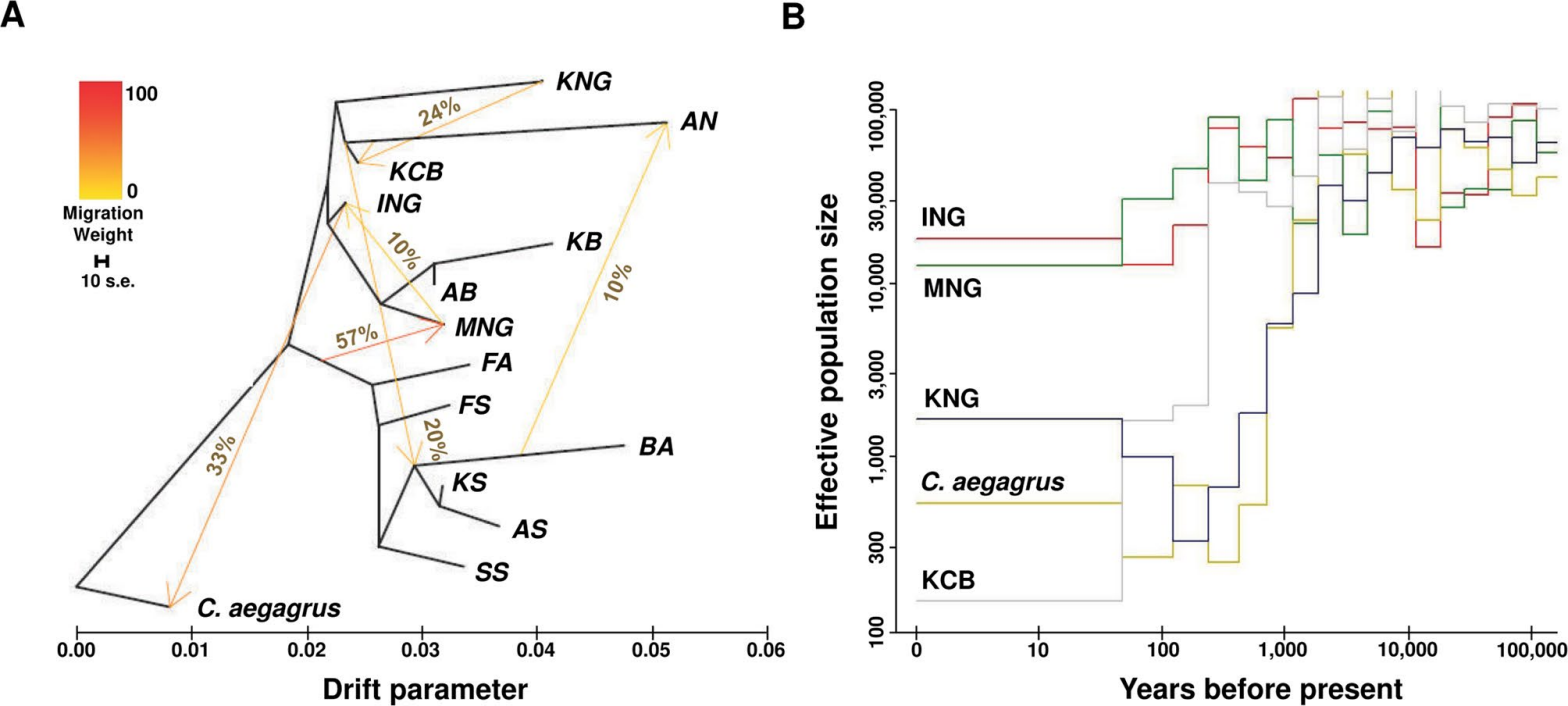
Inferred patterns of gene flow, genetic drift, and effective population size. **(A)** Pattern of population splits and mixture among 14 goat populations. The drift parameter is proportional to effective population size. The migration proportion, above the arrow, indicates the fraction of ancestry derived from the source population. The scale bar represents the standard error calculated 10 times from the sample covariance matrix. **(B)** Inferred demographic history for *C. aegagrus*, KCB, and indigenous breeds (ING, MNG, and KNG) (See **Data sheet 1: Figure S7 and Table S9**).

A maximum likelihood tree indicative of the genetic relationships among populations with directions of genetic drift and gene flow was reconstructed using TreeMix v1.13 (Pickrell and Pritchard, 2012) (**Figure 3A** and **Data sheet 1: Figure S5**). *C. aegagrus* was used as the root, and the block size for estimating the covariance matrix was chosen as 200 Kb, in consideration of the LD. The number of migration events was calculated as six, considering the complexity of our goat populations. The scale bar in the upper left corner represents the standard error of the tree, which represents the variation width of the tree estimated from the 10-time calculations. The reliability of this maximum likelihood tree was confirmed *via* multiple repeats (**Data sheet 1: Figures S6A–B**). To further validate the migration edges identified in the maximum likelihood tree, we conducted the Patterson’s D-statistic test (Durand et al., 2011) and the 3-population test (Reich et al., 2009) (**Data sheet 2**). The demographic history of each population was estimated using PopSizeABC (Boitard et al., 2016) (**Figure 3B** and **Data sheet 1: Figure S7**). The mutation rate of a base per generation was calculated as 1e-8, and the lower and upper bounds of the recombination rate were calculated as 1e-9 and 1e-8, respectively. The criterion of minor allele frequency was less than 0.2, and the segment size was 2,000,000. These demographic estimates were obtained through 100,000 iterations (**Data sheet 1: Figure S8 and Table S9**).

### Detection of Selection Signals and Gene-Set Enrichment Analysis

Cross-population extended haplotype homozygosity (XP-EHH) and cross-population composite likelihood ratio (XP-CLR) methods were analyzed using Selscan v1.1.0b (Szpiech and Hernandez, 2014) and XP-CLR v1.0 (Chen et al., 2010), respectively. The genetic positions were assumed to be equivalent to the physical positions due to the absence of a genetic map (1Mb = 1cM). The raw scores of the XP-EHH were standardized to the mean and the standard deviation, and –log(1 − *p*-value) of the two-tailed test was calculated through the empirical distribution (**Data sheet 1: Figure S9**). Based on this *p*-value, the outlier regions belonging to the top 0.1% were classified into specific candidate regions for further analysis, and the genes closest to these regions were designated as putative selected genes (**Data sheet 3**). The XP-CLR analysis was calculated by sliding 5 Kb with a window size of 10 Kb. A maximum of 2,000 SNPs were considered for each window, and a correlation level of 0.95 was used. The outlier regions with the top 0.1% of the raw scores were regarded as putative selection regions (**Data sheet 1: Figure S10**), and the closest genes to these candidate regions were designated as selected putative genes (**Data sheet 1: Figures S11A–J** and **Data sheet 4**). The number of selected genes detected in 10 goat populations by these two methods is summarized in **Data sheet 1: Table S10**. To identify the patterns of the adaptation process, we pooled the candidate genes detected by the XP-CLR and XP-EHH methods into one gene set for each population and performed gene set enrichment analysis (GSEA) for each pooled gene set using GeneTrail2 v1.5 (Stöckel et al., 2016) These candidate genes were grouped into various categories involved in similar functions, pathways, and biological processes through the Kyoto Encyclopedia of Genes and Genomes pathway database and the Gene Ontology database. The statistical significance level for the categories was a *p*-value of about 0.05 adjusted using the Benjamini-Hochberg method (**Table 1** and **Data sheet 5**).

**Table 1:** Significantly enriched terms identified in Korean indigenous goats (KNG) through gene set enrichment analysis (GSEA) (see Data sheet 5 for summary of all enriched terms).

Selected terms	Number of selected genes^a^	Adjusted *p*-value range^b^	Selected breeds^c^
*Salmonella *infection	11	(0.0170, 0.0648)	CA, IN, MN, TB, KB, TS, KS, AN, BA
Dilated cardiomyopathy (DCM)	21	(0.0002, 0.0318)	CA, IN, MN, KCB, TB, KB, TS, KS, AN
Hypertrophic cardiomyopathy (HCM)	15	(0.0041, 0.0318)	CA, IN, MN, TB, KB, TS, KS, AN
Arrhythmogenic right ventricular cardiomyopathy (ARVC)	17	(0.0001, 0.0575)	CA, IN, MN, TB, KB, TS, KS, AN, BA

a The total number of selected genes enriched in the selected term.

b The range of the minimum and maximum values of the adjusted p-values which each selected breed has for the selected term.

c Abbreviations in the selected breeds column means: IN is Iranian native goat, MN is Moroccan native goat, CA is C. aegagrus, KCB is Korean crossbred, KB is Korean Boer, KS is Korean Saanen, BA is British Alpine, AN is Anglo-Nubian, TB is the entire Boer group, and TS is the entire Saanen group.

## Results

### Data Collection, Re-Sequencing, and Identification of SNPs and INDELs

We generated whole-genome data for 46 goats and collected publicly available whole genome data for an additional 90 goats (**Data sheet 1: Table S1**). Our whole-genome data of the 136 individual goats covered 10 goat breeds [*C. aegagrus*, Iranian indigenous goats (ING), Moroccan indigenous goats (MNG), Korean indigenous goats (KNG), Korean crossbred (KCB), Saanen, Boer, British Alpine (BA), French alpine (FA) and Anglo-Nubian (AN)]. The Saanen and the Boer breeds constituted four sub-groups [Swiss Saanen (SS), Australian Saanen (AS), Korean Saanen (KS), and French Saanen (FS)] and three sub-groups [Australian Boer (AB), Korean Boer (KB), and Swiss Boer (SB)]. In total, 50.13 billion reads of 136 goat samples were aligned to the goat reference genome CHIR v2.0 (Dong et al., 2013). The average alignment rate was 99.47%, and it covered 98.61% of the reference genome (**Data sheet 1: Tables S2 and S3**). The average depths of the reads that removed potential PCR duplicates were 13.07X in the 90 publicly available goats and 12.14X in the 46 newly sequenced goats. To exclude as many false-positive called variants as possible, we strictly performed various filtering processes because the depths were not high (see “Materials and Methods”). After the variant calling and the filtering processes, a total of 5,629,521 INDEL variants and 39,830,354 bi-allelic SNPs were finally identified. Breed-specific SNPs were then extracted from the bi-allelic SNPs (**Figure 1B** and **Data sheet 1: Tables S4 and S5**). The numbers of bi-allelic SNPs were markedly different between commercial and indigenous breeds (including *C. aegagrus*). In the commercial breeds, the number of bi-allelic SNPs was at least 20% fewer than those of the indigenous breeds and detection in exon regions was also at least 32% less (**Data sheet 1: Table S6**). These lower tendencies in commercial breeds are considered to be the result of efforts to maintain breed homogeneity through artificial selection. One of the indigenous breeds, KNG, showed the highest number of bi-allelic SNPs (37,715,208) and missense mutations (188,265) except for *C. aegagrus*. Considering that the reference genome, China’s Yunnan black goat, has the same black coat color as KNG and the origin of KNG is indirectly related to China, these observations suggest that KNG possesses many SNPs that might have a functional influence on the formation of its unique genomic characteristics. From the following analysis, we used 38,658,962 bi-allelic autosomal SNPs, with an average distance of 64.88 bases between SNPs. This data set covered a significant portion of the reference genome. Additional results and discussions for other breeds are provided in **Data sheet 1: Note S1.2**.

### General Genomic Characteristics

To obtain a catalog of general genomic characteristics of the 10 goat breeds comprising the 15 goat populations, we estimated nucleotide diversity (π), inbreeding coefficient (F), and linkage disequilibrium (LD) (**Data sheet 1: Table S7**). The three estimates were quite variable between the populations. The π was the highest in ING and KCB, at 0.001908 and 0.001804, respectively, while the F was the highest in *C. aegagrus* and ING, at 0.0682 and 0.0622, respectively. The average LD patterns showed rapid declines within 50 Kb in all populations and, except for AN, BA and KNG, reached a plateau at around 200 Kb, implicating independent haplotype structures (**Data sheet 1: Figures S1A–D**). The average LD up to 500 Kb was the highest in AN and BA, at 0.3275 and 0.2888, respectively. In this catalog, KNG exhibited the distinctive genomic characteristics close to an isolated population. Among the indigenous breeds, the LD pattern of KNG was the highest at 0.0884, while the π and the F were the lowest, at 0.001472 and 0.01661, respectively. The higher LD indicates that KNG had initiated its breeding history with a limited number of founders in which recombination events occurred infrequently (Chakraborty and Deka, 2005), and has formed a comparatively homogeneous genome until now without few external pressures. The reduced π and F also indicate that KNG is a homogeneous population which has a relatively small number of homozygous genotypes. Along with the detection of the largest number of bi-allelic SNPs (**Figure 1B** and **Data sheet 1: Table S5**), these results suggest that KNG possesses many distinctive SNPs formed by their environmental influence. Moreover, the lower π was consistent with a previous study reporting that KNG has a lower genetic diversity than other Asian goat populations (Odahara et al., 2006). Additional results and discussion on the genomic characteristics for other goat breeds are provided in **Data sheet 1: Note S1.3**.

### Population Differentiation and Genic Structure

To obtain a refined picture of the 15 goat populations, we examined the patterns of genetic differentiation and genomic structure using reconstructed tree analysis (Gascuel, 1997), structure analysis (Raj et al., 2014), principal component analysis (PCA), and fixation index value (Fst) (Weir and Cockerham, 1984). These analyses revealed that KNG has genomic characteristics distinct from those of other goat populations (**Figures 2A–D**). The reconstructed tree showed that seven goat breeds, except for the Saanen and Alpine breeds, form their own clade which is genetically distinguished from each other (**Figure 2A**). The Saanen and Alpine breeds, improved similarly for the dairy purpose, formed three sister clades within a common large clade. In the structure analysis calculated ranging from *K* = 2 to *K* = 10 (**Figure 2B** and **Data sheet 1: Figure S3**), we obtained the most reasonable biological interpretation at *K* = 7. At *K* = 2, KNG, ING, and KCB were separated with having a common genomic composition (blue color). With increasing *K* values, *C. aegagrus*, Saanen and Boer breeds were further separated, and at the *K* = 7, AN was lastly separated with highly mixed genomic compositions observed. We found that KNG has almost a single genomic composition that is not mixed with other goat breeds. This finding indicates that a substantial portion of the KNG’s genome is distinct from those of other goat breeds. In addition, the result that the KNG’s genomic composition coincided with one of ING’s, suggests that KNG originated from the Iranian region where *C. hircus* appeared. The PCA clarified the complex stratifications of 15 goat populations. The first PC in **Figure 2C**, explaining 38.84% of the total genetic variation, separated *C. aegagrus* the farthest to the left and KNG the farthest to the right. The second PC, explaining 16.13% of the total genetic variation, separated Boer and Saanen breeds. **Figure 2D**, which excluded the out-group *C. aegagrus*, distinguished this complex structure in more detail. Centered on ING nearest to the wild-type, Boer breeds and Saanen breeds were separated from each other up and down, and then KNG was separated to the rightmost. The KCB, which was formed by hybridization of the KNG with various commercial breeds, was positioned between ING and KNG (**Figure 2D**). The Fst, calculated in a pair-wise manner, supported these qualitative distinctions (**Data sheet 1: Table S8**). The KNG showed the highest differentiation level between the 14 goat populations. The KNG had the highest differentiation level with BA (0.1908), which was the farthest from KNG, and had the lowest differentiation level with KCB (0.0733), which was the nearest to KNG, as shown in **Figures 2A–D**. Our refined picture indicates that KNG has unique genomic characteristics, and it suggests that the KNG has formed its own genome by accumulating the pressure of their local environment for a long period time, with little interaction with other goat populations. Additional results and discussion on the genomic status of other goat populations are provided in **Data sheet 1: Note S1.4**.

### Gene Flow and Demographic History

To visualize the genetic interaction of the 14 goat populations (excluding SB), we constructed a maximum likelihood tree using TreeMix (Pickrell and Pritchard, 2012) (**Figure 3A** and **Data sheet 1: Figure S5**). In this dendrogram, *C. hircus* was differentiated from *C. aegagrus* and then largely divided into the dairy breed and the meat type breed. KNG was directly differentiated from *C. aegagrus* and later ING, and showed an independent long branch indicating a high level of genetic drift. We found evidence that *C. aegagrus* and the indigenous breeds (ING and MNG) have interacted with each other, but no evidence that KNG has interacted with other goat breeds, except for KCB. This evidence was also not detected in additional analyses using the D-statistic (Durand et al., 2011) and 3-population (Reich et al., 2009) tests. These two tests supported the hypothesis that KNG has interacted only with KCB (**Data sheet 2**). Our result provides genomic evidence for existing reports that KNG has not gone through any genetic interchanges with imported breeds since its influx into the Korean Peninsula (Son, 1999; Kim et al., 2011).

We inferred the effective population size (N_e_) over the past time, in order to clarify the genetic drift which indigenous breeds and *C. aegagrus* have experienced (**Figure 3A**). The amount of genetic drift depends on the N_e_ (Ewens, 1990; Ballou et al., 2010; Frankham et al., 2010; Gasca-Pineda et al., 2013) (**Figure 3B** and **Data sheet 1: Figures S7A–D**). The reconstructed N_e_ patterns showed the domestication event between *C. aegagrus* and *C. hircus*, and a demographic event of KNG. The *C. aegagrus* maintained a high N_e_ for a long time, despite the appearance of *C. hircus*, which was domesticated about 10,000 years ago. However, the N_e_ started to decrease sharply about 1,000 years ago and has remained low until now. During the same period, the N_e_ of ING and MNG (both of which belong to *C. hircus*) increased about 1.5 times, and their genetic diversity has been maintained without loss until now. The crossing pattern of these N_e_ between *C. aegagrus* and *C. hircus* indicates the increased utilization of *C. hircus* and the decreased utilization of *C. aegagrus*, due to the successful domestication of *C. hircus*. Notably, at the time the N_e_ of these indigenous breeds began to increase, KNG experienced a serious loss of genetic diversity. This period nearly coincided with the time when KNG was estimated to be introduced into the Korean Peninsula (about 2,000 years ago) (Kang, 1967; Son, 1999). Since that time, the N_e_ of KNG steadily decreased until 100 years ago. At present, the N_e_ has increased slightly, but it showed still much lower than those for other indigenous breeds (**Data sheet 1: Table S9**). This N_e_ pattern represents that KNG had experienced a genetic bottleneck event during its influx into the Korea Peninsula and has relatively well adapted to the Korean environment since then. The 90% credible intervals of the estimated N_e_ for each population are displayed in **Data sheet 1: Figure S8**, and additional results and discussion for other goat populations are provided in **Data sheet 1: Note S1.5**.

### Detection of Selection Signals and Selective Sweep Regions in KNG

Nature selects a genomic region associated with specific traits such as disease or parasite resistance and temperature, or high-altitude adaptation, in order to increase the organisms’ chance of survival or reproduction in a particular environment (Futuyma, 2009). In the case of an isolated population, their genome is more susceptible to natural selection due to an environment with low confounding effects (Losos and Ricklefs, 2009; Pergams and Lawler, 2009). With this in mind, we compared the genome of KNG with those of 10 goat populations in order to uncover selection signatures of KNG. The 10 populations were *C. aegagrus*, ING, MNG, KCB, AN, BA, KS, KB, the entire Saanen group (AS, KS, SS and FS), and the entire Boer group (AB, KB and SB). To consider the overall genomic characteristics, the sub-populations of Boer and Saanen were pooled as the entire Boer and Saanen groups, respectively. We then searched for extended linked regions with extreme haplotype homozygosity and highly differentiated regions with variations of allele frequency, using cross-population extended haplotype homozygosity (XP-EHH) (Sabeti et al., 2007) and cross-population composite likelihood ratio (XP-CLR) (Chen et al., 2010) analyses. The XP-EHH method, based on the extended haplotype homozygosity concept, is not sensitive to allele frequencies and is effective for the unreliable demographic model (Sabeti et al., 2007). The XP-CLR method, based on the composite likelihood ratio test, has the advantage of effectively detecting selective sweep regions when a population has a simple structure, a low migration rate, or difficulty in estimating the local recombination rate (Racimo, 2016). Therefore, these methods were appropriate for our study. Particularly, the approach combining these two methods has been reported to be able to increase the power to pinpoint selected regions, and has been used widely to uncover genes involved in local adaptations (Vatsiou et al., 2016). After analysis, we set a strict cut-off line in order to exclude false-positive results due to the genetic drift as many as possible. We considered outlier regions belonging to the top 0.1% of the empirical distributions of XP-EHH and XP-CLR statistics as candidate regions (**Data sheet 1: Figures S9 and S10**, and **Data sheet 3** and **4**). Genes corresponding to these regions were annotated as candidate selected genes (**Data sheet 1: Figures S11A–J and Table S10**). The candidate genes derived from these two methods were pooled into one gene set for each population, in order to consider all genes that had undergone recent, soft, or hard sweeps. Then, gene set enrichment analysis (GSEA) (Stöckel et al., 2016) was performed for each gene set of each population to search for evidence of adaptation processes due to environmental selection. As a result, we found that KNG has selection signals for *Salmonella* infection pathway and cardiomyopathy pathway, respectively (**Table 1** and **Data sheet 5**).

**Figure 4:**
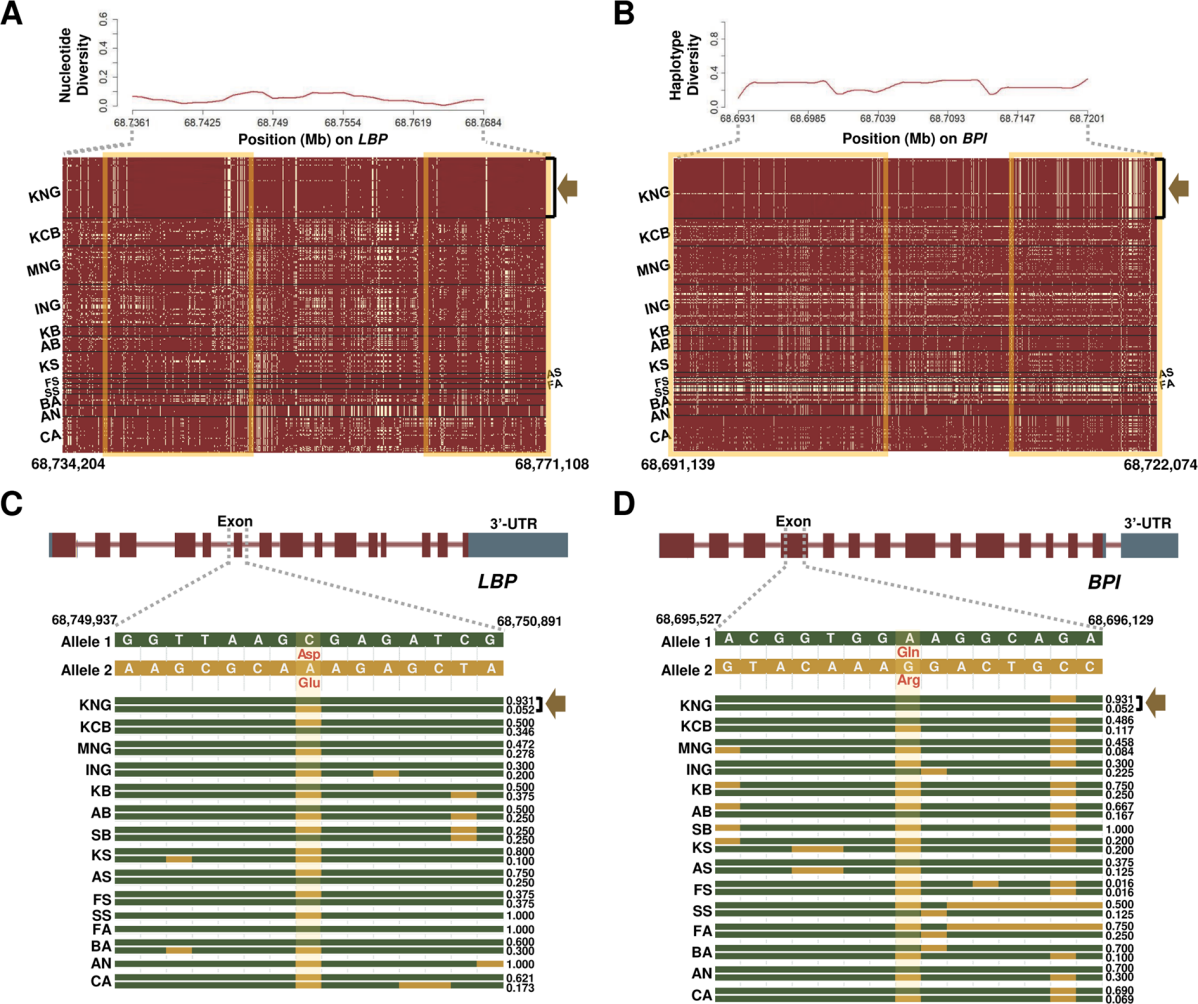
Selection signature for *Salmonella* infection in KNG. **(A)** Nucleotide diversity (above) and haplotype sharing (bottom) patterns for the region of 68,734,204-68,771,108-bp of the LBP gene located on chromosome 13. **(B)** Haplotype diversity (above) and haplotype sharing (bottom) patterns for the region of 68,691,139-68,722,074-bp of the BPI gene located on chromosome 13. In the haplotype sharing plots, the yellow rectangle highlights the pattern in which KNG is differentiated from other goat populations. **(C–D)** Gene structures and haplotype frequencies of regions containing a missense SNP in LBP and BPI genes, respectively. The missense SNPs, highlighted in yellow, represent p.Asp217Glu on the 68,750,237 bp position (LBP) and p.Gln104Arg on the 68,695,875 bp position (BPI) (See **Data sheet 6**).

**Figure 5:**
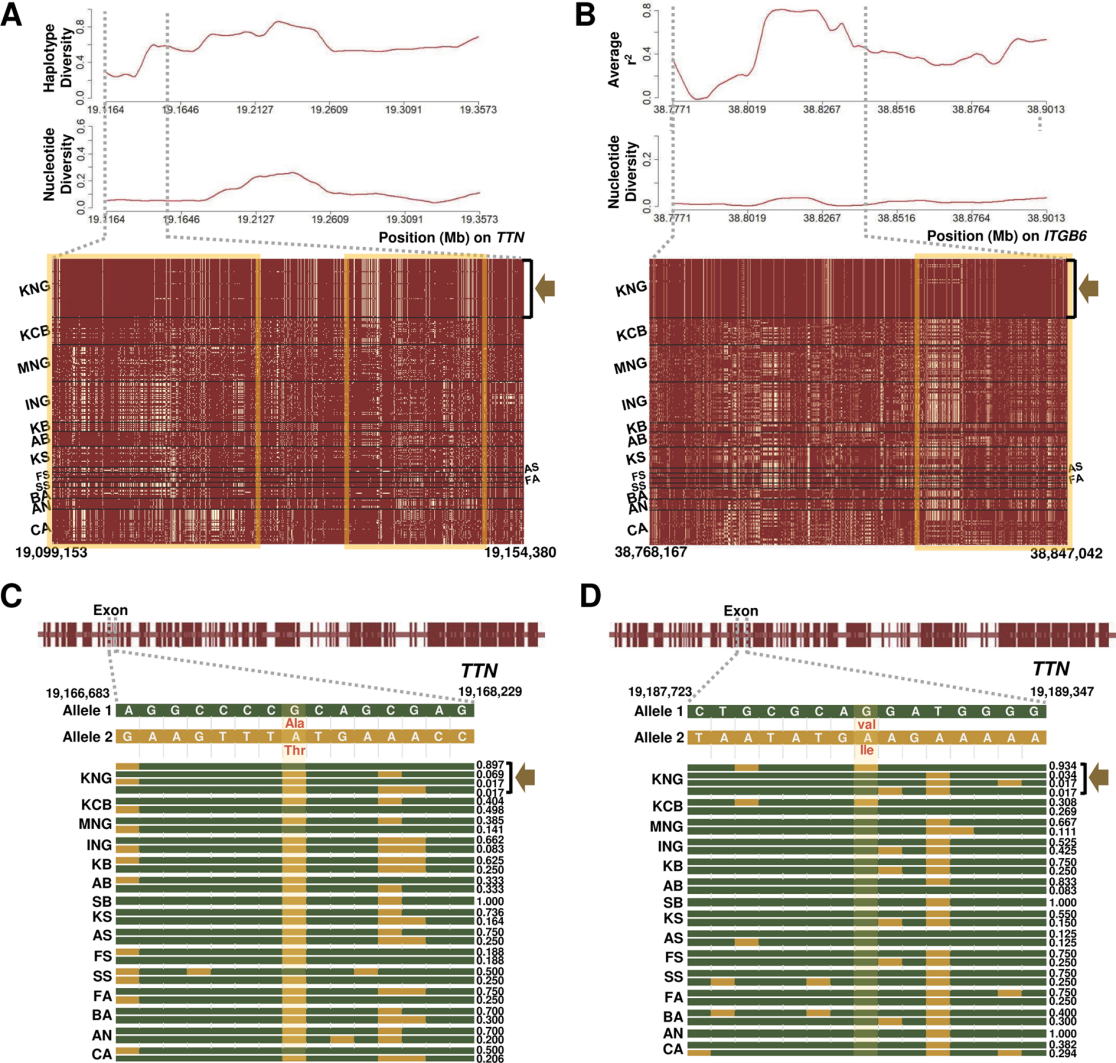
Selection signature for cardiomyopathy in KNG. **(A)** Haplotype and nucleotide diversity patterns (above) and haplotype sharing pattern (bottom) for the 19,099,153-19,154,380-bp region of the TTN gene located on chromosome 2. **(B)** Average linkage disequilibrium and nucleotide diversity patterns (above) and haplotype sharing pattern (bottom) for the 38,768,167-38,847,042-bp region of the ITGB6 gene located on chromosome 2. In the haplotype sharing plots, the yellow rectangle highlights the pattern in which the KNG is differentiated from other goat populations. **(C–D)** Gene structures and haplotype frequencies of regions containing missense SNPs in the TTN gene. The missense SNPs, highlighted in yellow, represent p.Val7638Ile on the 19,188,702 bp position and p.Ala3702Thr on the 19,167,388 bp position, respectively (See **Data sheet 6**).

Selection leaves detectable patterns in linkage disequilibrium, genetic diversity, and site frequency spectrum at the genome level, since it modifies the neutral pattern of the genomic region under the neutral theory of molecular evolution (Ross-Ibarra et al., 2007; Qanbari et al., 2010). When an allele frequency of a specific locus is affected by the selection, allele frequencies of closely linked loci around the locus are also affected, unlike the random process of genetic drift (Nielsen et al., 2005; Gianola et al., 2010; Qanbari et al., 2011). Therefore, we further investigated patterns of nucleotide diversity, linkage disequilibrium, haplotype diversity, and Fst of LBP, BPI, ITGB6, and TTN genes, among KNG’s candidate genes enriched in *Salmonella* infection and cardiomyopathy pathways (**Table 2**, **Figures 4A–D**, **Figures 5A–D**, and **Data sheet 1: Figures S12–S16**). These genes revealed traces of the environmental selection with the genetic drift that KNG underwent.

**Table 2:** Candidate genes showing distinct patterns among genes involved in *Salmonella* infection and cardiomyopathy terms of KNG (See Data sheet 3 and 4 for summary of all selected genes).

Selected genes	Association	CHR^a^	XP-CLR	XP-EHH		Candidate SNP position	Selected breeds^d^
			Score range^b^	Score range^b^	*p*-value range^c^		
LBP	*Salmonella* infection	13	(9.27, 9.27)	(3.31, 4.52)	(2.91, 3.33)	68,750,237 (p.Asp217Glu)	CA, IN, MN, KCB, TB, KB
BPI	Antimicrobial activity	13	(6.74, 9.96)	(3.31, 4.87)	(2.91, 3.33)	68,695,875 (p.Gln104Arg)	CA, IN, MN, KCB, TB, TS, BA
ITGB6	Cardiomyopathy	2	(8.11, 11.57)	(3.4, 3.4)	(3.16, 3.16)	–	CA, IN, MN, KCB, TB, KS, AN, BA
TTN	Cardiomyopathy	2	(7.94, 13.81)	(3.46, 3.72)	(2.9, 3.45)	19,127,870 (p.Ile1202Thr) 19,167,388 (p.Ala3702Thr) 19,188,702 (p.Val7638Ile)	CA, IN, MN, KCB, TB, TS, KS

Dash (-) indicates non-significant results

a Chromosome

b The range of minimum and maximum values of XP-CLR and XP-EHH scores which each selected breed has for the selected gene.

c The range of minimum and maximum values of - log p-values for the XP-EHH scores, derived from the empirical distribution.

d Abbreviations in the selected breeds column means: IN is Iranian native goat, MN is Moroccan native goat, CA is C. aegagrus, KCB is Korean crossbred, KB is Korean Boer, KS is Korean Saanen, BA is British Alpine, AN is Anglo-Nubian, TB is the entire Boer group, and TS is the entire Saanen group.

### The Adaptive Characteristics of KNG to *Salmonella* Infection

In previous experimental studies, Jang and Kang reported that *Salmonella* species were not isolated from the feces of 49 KNG with diarrhea symptoms or 620 healthy KNG (Jang, 1995; Kang and Tak, 1996). Lee suggested that the reason for the absence of *Salmonella* infection in KNG was due to their excellent antibody productivity and inherent resistance factors (Lee et al., 2000). Through the selection analysis and the GSEA comparing KNG with 10 goat populations, we found that KNG showed selection signals for the *Salmonella* infection pathway in nine goat populations, excluding KCB (**Table 1** and **Data sheet 5**). The KCB, which was formed by hybridizing KNG with other goat breeds, is presumed to preserve a substantial portion of the genomic characteristics derived from KNG.

We further confirmed the LBP (Lipopolysaccharide Binding Protein) and BPI (Bactericidal Permeability Increasing Protein) genes, among 11 genes showing selection signals in the *Salmonella* infection pathway (**Table 2** and **Figures 4A, B**). The LPB gene, which encodes a lipopolysaccharide-binding protein, binds to the lipid A moiety of bacterial lipopolysaccharides to promote the release of cytokines, and the BPI gene, which encodes the bactericidal/permeability-increasing protein, regulates the LPS-dependent monocyte responses by binding to LPS along with the product of the LBP gene. The LBP gene plays an important role in the innate immune response of organisms (Wilde et al., 1994; Eckert et al., 2013), and the BPI gene plays an important role in antimicrobial activity against gram-negative organisms, as a paralogue of the LBP gene (Brister et al., 2014). Throughout the entire region of each gene, KNG showed the low nucleotide diversity and haplotype diversity patterns and presented a distinctive haplotype sharing pattern (a yellow square in **Figures 4A, B**, and **Data sheet 1: Figures S12A–D**). The pattern of the almost pure haplotype homozygosity, which distinguished noticeably from other populations, provides evidence that KNG has experienced the strong genetic drift which extensively affected the frequency of the alleles. Additionally, we discovered one selective sweep region with one missense variant in each gene, where KNG has been affected by their environment (**Figures 4C, D**). One missense variant was found in the 68,750,237 bp position (p.Asp217Glu) of the LBP gene (**Figure 4C**), and the other was found in the 68,695,875 bp position (p.Gln104Arg) of the BPI gene (**Figure 4D**). The haplotype frequencies of these variants in the LBP and BPI genes were the lowest in KNG, at 0.052 and 0.017, respectively, when excluding FA, SA, FS, and SB, which have low sample sizes. Conversely, the haplotype frequencies without these variants were the highest in KNG, at 0.930 and 0.983, respectively.

We confirmed that these LBP and BPI genes have been fixed or are being fixed in the direction of conserving their function in KNG. These results suggest that KNG has been more stabilized than other breeds for antimicrobial activity against gram-negative organisms as well as the innate immune response to *Salmonella* infection. We propose that KNG has accumulated local environmental pressure along with gene drift and has partially adapted to the *Salmonella* infection. In **Figures 4C, D**, only the top two haplotype frequencies for each goat population are illustrated, due to the limitations of illustration size. All haplotype frequencies are provided in **Data sheet 6**.

### The Adaptive Characteristics of KNG to Cardiomyopathy Challenge

Cardiomyopathy is any disease affecting the muscle, size, and shape of the heart, and is presents as three main types: dilated cardiomyopathy (DCM), hypertrophic cardiomyopathy (HCM), and arrhythmogenic right ventricular cardiomyopathy (ARVC) (Rush et al., 2002). It is mostly idiopathic, and symptoms are different ranging from no symptoms, to difficulty breathing, to sudden death. This disease has been reported to occur not only in humans but also in dogs and cats (Broschk and Distl, 2005; Meurs et al., 2007), and even in the Saanen goat breed (Tontis et al., 1992). Using the selection analysis and the GSEA, we found that KNG has selection signals for all of DCM, HCM, and ARVC pathways in eight goat populations excepting for KCB and BA (**Table 1** and **Data sheet 5**). The candidate genes for KCB and BA were significantly enriched only in DCM pathway and ARVC pathway, respectively. Although further research is needed, KCB is presumed to preserve a significant portion of the genomic characteristics derived from KNG, and BA is presumed to have partially adapted to this disease due to the artificial or environmental effects (**Figure 3A**).

We further confirmed the ITGB6 (Integrin Subunit Beta 6) and TTN (Titin) genes among the several genes showing selection signals in the three cardiomyopathy pathways (**Table 2** and **Figures 5A, B**). The ITGB6 gene, which encodes a protein of the integrin superfamily, is involved in all of the DCM, HCM, and ARVC pathways, and it has been reported to be particularly closely related to the ARVC pathway (O’Leary et al., 2015; Stelzer et al., 2016). The TTN gene, which encodes a large abundant protein of striated muscle containing cardiac muscle tissues, is involved in the DCM and HCM pathways, and it has been reported as one of the positively selected genes that influence cardiomyopathy in a bear breed (Liu et al., 2014). In both genes, KNG showed the lowest nucleotide diversity and haplotype diversity patterns and presented an almost pure haplotype sharing pattern as a result of the genetic drift (A yellow square in **Figures 5A, B** and **Data sheet 1: Figures S13–S16**). In addition, KNG showed traces of selective sweeps with three missense variants in the TTN gene (**Figures 5C, D** and **Data sheet 1: Figure S16A)**. We screened the regions containing these missense mutations along with haplotype frequencies. The haplotype frequency with a missense SNP (p.Ile1202Thr) found at the 19,127,870 bp position was highest in KNG at 0.948, followed by in KCB and KB at 0.538 and 0.375, respectively. (**Data sheet 1: Figure S16A**). This missense SNP showed a tendency to hitchhike the SNPs of 19,127,266 bp and 19,128,208 bp positions together. Another missense SNP (p.Ala3702Thr) found at the 19,167,388 bp position showed a tendency to replace the SNP of 19,167,677 bp position with the reference SNP (**Figure 5C**). The haplotype frequency of this region was the highest in KNG at 0.897, followed by in SS and KCB at 0.498 and 0.500, respectively. Most goat populations possessed this missense mutation, but KNG maintained this SNP as the reference variant with a high frequency. The other missense SNP was found at the 19,188,702 bp position (**Figure 5D**). This variant showed a tendency to replace the SNP of 19,188,088 bp position with an alternative SNP and the SNP of 19,189,131 bp position with a reference SNP, respectively. The haplotype frequency was the highest in KNG at 0.934, followed by in KCB at 0.404. We further identified the region where the ITGB6 gene has been affected by the selective sweep. KNG showed the highest average LD with the lowest nucleotide diversity in the region of 38,805,100 bp–38,833,000 bp (**Figure 5B** and **Data sheet 1: Figures S13A–C and S14A–B**).

We confirmed that these ITGB6 and TTN genes have been affected by the local environment along with the genetic drift. Particularly, the coexistence of three missense SNPs with the highest and the lowest frequencies in KNG suggests that this TTN gene has been playing a functional role in adapting to cardiomyopathy as one of several candidate genes. Based on our genomic research, we propose that KNG has partially adapted to the cardiomyopathy under their various environmental pressure.

## Discussion

### Genomic Characteristics of KNG

Domestication and subsequent geographical expansion have generated a variety of indigenous livestock breeds. These breeds have accumulated multiple genetic variations affecting a variety of traits over time and have developed their own unique genomic characteristics in the course of enhancing their fitness in different local environments. These genomic characteristics are important as a genomic basis for coping with future threats to the species arising from environmental change (Benjelloun et al., 2015), but are rapidly disappearing due to extensive crossbreeding and substitution with imported breeds. Therefore, to reveal their unique genomic characteristics, many genomic studies have been carried out in various indigenous livestock: cattle (Browett et al., 2018; Weldenegodguad et al., 2018); chicken (Johansson and Nelson, 2015; Walugembe et al., 2018); sheep (Yang et al., 2016; Edea et al., 2017); and goat (Benjelloun et al., 2015; Cao et al., 2019). In this context, our study focused on identifying KNG and revealed their distinct genomic characteristics.

To investigate KNG in detail, we utilized the whole-genome variations of a total of 10 goat breeds, including three indigenous breeds (KNG, ING, and MNG), six commercial breeds (Saanen breed, Boer breed, AN, BA, FA, and, KCB), and one ancestral species (*C. aegagrus*). A total of 38,658,962 bi-allelic SNPs were detected in 29 autosomes of 10 breeds, and we identified that these SNPs covered a considerable portion of their reference genome at an average distance of 64.88 bases between SNPs (**Data sheet 1: Table S4**). With the exception of their ancestral species, the number of bi-allelic SNPs was the highest for KNG (37,715,208) and followed by ING, KCB, and MNG (35,742,191, 33,464,841, and 32,914,220) (**Figure 1B** and **Data sheet 1: Table S5**). In respect of π and LD calculated using these bi-allelic SNPs, the KNG exhibited the lowest π (0.001472) and the highest LD (0.088431) among three indigenous breeds (**Data sheet 1: Table S7**). Particularly, the KNG’s π value was consistent with the adjusted π value reported by a previous study (calculation window size adjusted from 1Mb to 100Kb) (Lee et al., 2016). Considering their low π and many SNPs, our results indicate that the KNG has a fair number of homozygous SNP variants distinguished from other goat breeds, relatively. In addition to this, the high LD value implies that their homozygous SNP variants have a high level of association with each other due to evolutionary pressures such as selection or genetic drift.

The population analyses conducted through various methodologies supported our hypothesis that the KNG has unique genomic characteristics, which are distinct from those of other goat breeds. Within a large category of genomic diversity parameters, the genomic features of the eight goat breeds did not show large differences, but KNG and *C. aegagrus* showed distinctive genomic characteristics (**Figures 2A–D**). The KNG was separated to the rightmost in PCA, showed a near-identical genomic composition in structure analysis (**Figure 2B**), and exhibited high levels of genetic differentiation compared with other goat breeds (**Data sheet 1: Table S8**). Our results additionally confirmed that the genomic composition of KNG (blue color) coincided with one of ING, and another genomic composition of ING (green color) was consistent with one of their ancestral species (**Figure 2B**). Particularly, the ING inhabiting the region of Iran where *C. hircus* was first domesticated showed a linear relationship with the *C. aegagrus*, and they positioned at the center of the 10 goat breeds in the PCA (**Figure 2C**). Given the origins of *C. hircus* and KNG, these results suggest that the ING has maintained a substantial portion of genomic characteristics derived from its ancestral species since the domestication, and that the KNG has formed its own genomic characteristics since influx into the Korean Peninsula about 2,000 years ago (Tavakolian, 2000; Zeder and Hesse, 2000). Meanwhile, a previous study reported that the ING inhabiting the north of the Zagros mountain has the most similar genomic structure to their ancestor, *C. aegagrus* (Vahidi et al., 2014). In our study, the genomic compositions of ING samples were almost identical to those of ING samples which have been reported to be the indigenous goats of the north Zagros mountain. This result indicates that the ING samples were suitable for our study to compare KNG with various goat breeds, as the closest domesticated goats to their ancestral species.

From the analyses of the gene flow and N_e_, we revealed that the KNG’s unique genomic characteristics are associated, at least in part, with their isolated environment (**Figures 3A, B**). We confirmed that the KNG underwent a severe genetic bottleneck event as they entered the Korean Peninsula about 2,000 years ago (**Figure 3B**), and have experienced little genetic interactions with other breeds (only except for KCB) (**Figure 3A**). To clarify the interaction signals of KNG, the D-statistic (Durand et al., 2011) and 3-population (Reich et al., 2009) tests were also performed, but no signal was detected except for the KCB (**Data sheet 2**). These results indicate that the KNG has accumulated their local environmental pressure for a long time, and has developed their own genomic characteristics with little genetic interaction with other breeds. Also, as genomic evidence, these results support the previous studies which reported on the origin and isolated environment of KNG (Son, 1999; Tavakolian, 2000; Odahara et al., 2006). So far, we revealed the unique genomic characteristics of KNG through a comparison of 10 goat breeds. We expect that our detailed review for the KNG including other goat breeds would contribute to the establishment of biodiversity conservation strategies regarding indigenous goats.

### Adaptive Characteristics of KNG

During long-term adaptation to the various environments, indigenous livestock breeds have developed their own adaptive characteristics which enhance fitness to harsh environments or resistance to specific diseases. These characteristics have provided an important genetic basis for various breeding programs to improve livestock (Guan et al., 2016). Thus, to identify their adaptive characteristics, many studies on selection signatures have been conducted in various indigenous livestock: cattle (Taye et al., 2017); chicken (Lawal et al., 2018); goat (Guo et al., 2018); sheep (Liu et al., 2016); and pig (Li et al., 2014). From this perspective, our study compared KNG with other 10 goat breeds, and revealed that the KNG has selection signatures for *Salmonella* infection and cardiomyopathy pathways (**Table 1**).

*Salmonella* infection has effects ranging from growth delay to livestock death (Cummings et al., 2010). The identification of indigenous breeds adapted to this infection can be valuable in livestock breeding programs for enhancing the survival rate and preventing disease transmission. However, there have been few investigations into indigenous livestock breeds which carry this resistance, except for the Sri Lankan indigenous chicken (Weerasooriya et al., 2017) and the KNG (Jang, 1995; Kang and Tak, 1996; Lee et al., 2000). Although these two breeds have been reported to be resistant to *Salmonella* infection through experimental studies, their utilization in breeding programs has been limited due to the lack of genomic studies. In this study, we identified that the KNG exhibits selection signals with respect to the *Salmonella* infection pathway for nine goat breeds except for KCB (**Table 1**). To clarify the KNG’s selection signals, we further examined the LBP and BPI genes among their 11 candidate genes (**Table 2**). The KNG showed low nucleotide and haplotype diversity patterns and a unique haplotype-sharing pattern over the entire region of these genes (**Figures 4A, B**). Also, as a consequence of strong selection pressures, the KNG exhibited selective sweep regions with one missense SNP variant in each gene (**Figures 4C, D**). The haplotype frequencies containing these missense variants were the lowest in KNG when excluding FA, SA, FS, and SB with low sample sizes (**Data sheet 6**). Considering the functions of two genes, these results indicate that KNG has been more stabilized than other breeds for the antimicrobial activity to gram-negative organisms and the innate immune response to *Salmonella* infection. Our results provide genomic evidence to support previous biological studies, and statistically, propose that KNG has adaptive characteristics for *Salmonella* infection.

As one of the novel adaptive characteristics, we observed that the KNG has selection signals for all three types of cardiomyopathy pathways in eight goat breeds (**Table 1**). The exceptions were KCB and BA, and the KNG’s candidate genes were significantly enriched only in DCM pathway for KCB and only in ARVC pathway for BA. Among the KNG’s candidate genes, we further investigated the TTN (associated with DCM and HCM) and ITGB6 (associated with ARVC) genes which show distinctive selection patterns (**Table 2** and **Figures 5A, B**). For TTN, the KNG exhibited a trace of selective sweep together with hitchhiking effects in three missense SNP variants (**Figures 5C, D**, and **Data sheet 1: Figure S16A**). In the 38,805,100–38,833,000 bp region of the ITGB6 gene, the KNG showed the highest LD and almost pure haplotype patterns due to strong selection pressure (**Figure 5B**, and **Data sheet 1: Figure S14A-B**). These results indicate that the ITGB6 and TTN genes, particularly, have been playing a functional role in adapting to cardiomyopathy in the KNG. Based on our genomic research, we statistically propose that the KNG has partially adapted to cardiomyopathy.

In our results, the KNG did not show selection signals for *Salmonella* infection in KCB and for cardiomyopathy in KCB (HCM and ARVC) and BA (DCM and HCM). The KCB was recently formed by hybridization of KNG with other goat breeds, in order to improve various traits of the KNG (Lee et al., 2016). The KCB shared a large amount of genomic composition with KNG in structure analysis (**Figure 2B**) and exhibited a similar genomic characteristic to KNG in PCA (**Figures 2C, D**). Also, they showed substantial interactions with the KNG in gene flow analysis, Patterson’s D-statistic test, and the 3-Population test (**Figure 3A** and **Data sheet 2**). These results indicate that the KCB has acquired a considerable portion of their genome characteristics from KNG, and that the purpose of the crossbreeding program has been achieved to a large extent. However, considering their still high π (0.001908) and low LD (0.068539) (**Data sheet 1: Table S7**), it is suggested that the KCB need an additional breeding program to stabilize their genomic and adaptive characteristics. Meanwhile, the BA was developed in the Swiss and Austrian Alps in the early 1900s and introduced into Australia in about 1960. Our BA samples collected in Australia showed the lowest π (0.001251) and the highest LD (0.288801), except for the AN (π: 0.001117, and LD: 0.327566) (**Data sheet 1: Table S7**). These genomic characteristics imply that the BA had undergone significant genetic drift upon being introduced to Australia and have experienced multiple selection events. Our study confirmed the possibility that the BA may have partially adapted to cardiomyopathy in their environment, but we propose further research to clarify this adaptive characteristic, due to their small sample size.

Our study has several limitations. First, the SNP variants of some breeds (FA, AS, SS, FS, and SB) may have been affected by SNP ascertainment bias due to their small sample sizes. Their SNP variants may not have adequately represented their entire breeds, and some analysis results for them may have been distorted. Therefore, to minimize this problem, our study utilized these breeds as only references against which to compare the genomic characteristics of the other breeds. In contrast, we could avoid another SNP ascertainment bias due to SNP discovery protocols by using the whole-genome sequencing protocol. In the case of using Illumina’s Goat SNP50 BeadChip (Tosser-Klopp et al., 2014), which contains approximately 53,346 SNP variants, some results of the population analyses could be distorted due to this bias, since its SNP markers cover neither all goat breeds nor entire genomic regions (Lachance and Tishkoff, 2013). Second, some adaptive characteristics for KNG have been identified, but have not been validated by biological experiments. To minimize this limitation, we conducted rigorous statistical calculations. We compared the KNG with other 10 goat breeds by using two selection analysis methods, XP-CLR and XP-EHH, and detected candidate selected genes using strict cut-offs. We then confirmed the KNG’s adaptation signals through the GSEA of the candidate genes, and revealed the genomic regions affected by the selection pressure in some candidate genes. Despite these efforts, our results still require further experimental validation, but we anticipate that these candidate genes and their targeted genomic regions will be helpful in future experimental studies aimed at identifying the characteristics of KNG.

Although our study has some limitations, our catalog of genome characteristics of 10 goat breeds would provide the basis for establishing various appropriate breeding strategies. Also, our findings on the genomic and adaptive characteristics of the KNG will help to set directions of biodiversity conservation programs as well as crossbreeding and grading-up programs for improving goat breeds.

## Conclusion

The valuable genomic characteristics that indigenous breeds have accumulated for a long time are being threatened by crossbreeding with imported breeds with high productivity. Particularly in the case of Korea, KNG is rapidly being substituted with KCB, which was formed by hybridizing KNG with other breeds to improve KNG’s inferior commercial traits. Although their characteristics may not be commercially valuable, they could have unique abilities to survive in a particular environment or disease. In this respect, our research on the genomic population dynamics of KNG, including various goat breeds, provides an important basis for establishing a direction for biodiversity conservation strategies. Although our findings for adaptive characteristics have a limitation that is provided without biological validation, these are expected to not only provide new and other options to those seeking to improve the viability and the resilience of goats but also present targeted genomic regions to *in vivo* or *in vitro* studies trying to employ our hypothesis. In addition, our newly generated whole-genome data that is opened to the public database will contribute to the knowledge for further research.

## Ethics Statement

This study was carried out in accordance with the guidelines of the Institutional Animal Care and Use Committee (IACUC) and was approved by the National Institute of Animal Science, Rural Development Administration, Republic of Korea (Approval No: 2012-D-010). The animal preparation and experimentation were conducted in accordance with the protocol approved by the guidance of the IACUC.

## Author Contributions

NK conceived and supervised this project. J-YK, and SJ performed the data analysis and wrote the draft manuscript. KHK, and W-JL supported the data analysis and interpretation. H-YL assisted with the literature search and figure preparation. All authors discussed the results and read and approved the final manuscript.

## Funding

This project was supported by grants from the National Research Foundation of Korea (NRF-2014M3C9A3064552), the KRIBB Initiative program, and the Cooperative Research Program for Agriculture Science and Technology Development Project of Rural Development Administration (Republic of Korea) (No. PJ00868002).

## Conflict of Interest Statement

The authors declare that the research was conducted in the absence of any commercial or financial relationships that could be construed as a potential conflict of interest.

## Abbreviations

AB, Australian Boer; AN, Anglo-Nubian; ARVC, arrhythmogenic right ventricular cardiomyopathy; AS, Australian Saanen; BA, British Alpine; CA, *Capra aegagrus*; DCM, dilated cardiomyopathy; F, inbreeding coefficient; FA, French Alpine; FS, French Saanen; Fst, fixation index value; GATK, Genome Analysis Toolkit; GSEA, gene-set enrichment analysis; HCM, hypertrophic cardiomyopathy; He, expected heterozygous genotype frequency; Ho, observed heterozygous genotype frequency; ING, Iranian native goat; KB, Korean Boer; KCB, Korean crossbred; KNG, Korean native goat; KS, Korean Saanen; LD, linkage disequilibrium; MNG, Moroccan native goat; N_e_, effective population size; PCA, principal component analysis; π, nucleotide diversity; SB, Swiss Boer; SS, Swiss Saanen; XP-CLR, cross-population composite likelihood ratio; XP-EHH, cross-population extended haplotype homozygosity.

## Supplementary Material

Data sheet 1 Supplemental information including Note, Tables S1-S10 and Figures S1-S16 (DOC 8,082 kb).

Data sheet 2 The results of D-statistic and three-population tests (XLSX 191 kb).

Data sheet 3 The results of XP-EHH analysis (XLSX 483 kb).

Data sheet 4 The results of XP-CLR analysis (XLSX 255 kb).

Data sheet 5 The summary of gene set enrichment analysis (XLSX 39 kb).

Data sheet 6 The summary of six non-synonymous variants and their surrounding haplotype frequencies (XLSX 49 kb).
