# Supplementary material for: Discovery of Genomic Characteristics and Selection Signatures in Korean Indigenous Goats Through Comparison of 10 Goat Breeds
Source: Front Genet. 2019 Aug 8;10:699. doi: 10.3389/fgene.2019.00699 (PMC6694180; doi:10.3389/fgene.2019.00699)
Supplement: Data sheet 1 — Supplemental information including Note, Tables S1-S10 and Figures S1-S16 (DOC 8,082 kb). [file DataSheet_1.doc]

***Supplementary Material***

**Discovery of Genomic Characteristics and Selection Signatures in Korean Indigenous Goats through Comparison of 10 Goat Breeds**

**Article type:** ORIGINAL RESEARCH

**Authors and affiliations:**

Jae-Yoon Kim^1,2†^, Seongmun Jeong^1†^, Kyoung Hyoun Kim^1,2^, Won-Jun Lim^1,2^, Ho-Yeon Lee ^1,2^, and Namshin Kim^1,2*^

^1^Genome Editing Research Center, Korea Research Institute of Bioscience and Biotechnology (KRIBB), Daejeon 34141, Republic of Korea

^2^Department of Bioinformatics, KRIBB School of Bioscience, University of Science and Technology (UST), Daejeon 34141, Republic of Korea

^†^These authors contributed equally to this research

**Corresponding author:**

Namshin Kim, Genome Editing Research Center, Korea Research Institute of Bioscience and Biotechnology (KRIBB), Daejeon 34141, Korea, +82-42-879-8162, [deepreds@kribb.re.kr](mailto:deepreds@kribb.re.kr)

**Supplementary information includes:**

S1: Supplementary Note

S2: Supplementary References

S3: Supplementary Figures S1-S16

S4: Supplementary Tables S1-S10

S5. Supplementary Excel Tables, provided as additional files 2-6.

**Supplementary Information**

| **S1. Supplementary Note**……….…………...………………………….…………………. | | **3** |
| --- | --- | --- |
|  | 1.1 Data collection and brief sampling information…………………………………….. | **3** |
|  | 1.2 Identification of SNPs………………………………………………………………. | **3** |
|  | 1.3 General genomic characteristics…………………………………………………….. | **4** |
|  | 1.4 Population differentiation and genic structure………………………………………. | **5** |
|  | 1.5 Gene flow and demographic history………………………………………………… | **6** |
| **S2. Supplementary References**…………………………………………………………... | | **8** |
| **S3. Supplementary Figures S1-S16**……………………………………………………… | | **11** |
|  | 3.1 Supplementary Figure S1…………………………………………………………… | **11** |
|  | 3.2 Supplementary Figure S2…………………………………………………………… | **12** |
|  | 3.3 Supplementary Figure S3…..………………...……………………………………... | **13** |
|  | 3.4 Supplementary Figure S4…………………...………………………………………. | **14** |
|  | 3.5 Supplementary Figure S5……………………………...……………………………. | **15** |
|  | 3.6 Supplementary Figure S6……………………………………...……………………. | **16** |
|  | 3.7 Supplementary Figure S7……………………………………………...……………. | **17** |
|  | 3.8 Supplementary Figure S8……………………………………………………...……. | **18** |
|  | 3.9 Supplementary Figure S9…………………………………………………………… | **20** |
|  | 3.10 Supplementary Figure S10………………………………………………………… | **21** |
|  | 3.11 Supplementary Figure S11…………………………………………………………. | **22** |
|  | 3.12 Supplementary Figure S12………………………………………………………… | **26** |
|  | 3.13 Supplementary Figure S13………………………………………………………… | **27** |
|  | 3.14 Supplementary Figure S14………………………………………………………… | **28** |
|  | 3.15 Supplementary Figure S15………………………………………………………… | **30** |
|  | 3.16 Supplementary Figure S16………………………………………………………… | **31** |
| **S4. Supplementary Tables S1-S10**……………………………………………………….. | | **37** |
|  | 4.1 Supplementary Table S1…………………………………………………………….. | **37** |
|  | 4.2 Supplementary Table S2…………………………………………………………….. | **38** |
|  | 4.3 Supplementary Table S3…………………………………………………………….. | **42** |
|  | 4.4 Supplementary Table S4……………………………………………………………. | **44** |
|  | 4.5 Supplementary Table S5……………………………………………………………. | **46** |
|  | 4.6 Supplementary Table S6……………………………………………………………. | **47** |
|  | 4.7 Supplementary Table S7……………………………………………………………. | **51** |
|  | 4.8 Supplementary Table S8……………………………………………………………. | **52** |
|  | 4.9 Supplementary Table S9……………………………………………………………. | **53** |
|  | 4.10 Supplementary Table S10…………………………………………………………. | **56** |
| **S5. Supplementary Excel Tables………..**……………………………………………….. | | **57** |
|  | 5.1 Additional File 2……………………………..…........................................................ | **57** |
|  | 5.2 Additional File 3……………………………..……...………………………………. | **57** |
|  | 5.3 Additional File 4……………………………...……..………………………………. | **57** |
|  | 5.4 Additional File 5……………………………..…...…………………………………. | **57** |
|  | 5.5 Additional File 6……………………………..……...………………………………. | **57** |

**S1: Supplementary Note**

- 1. **Sample collection and brief sampling information**

The 46 newly re-sequenced goats consisted of 14 Korean indigenous goats (KNG), 10 Korean Saanen (KS), and 4 Korean Boer (KB), which have raised in Korea; and 5 Anglo-Nubian (AN), 5 British-Alpine (BA), 6 Australian Boer (AB), and 2 Australian Saanen (AS), which have raised in Australia (Additional file 1: Table S3). The 90 publicly available goats comprised: 15 Korean indigenous goats (KNG) which same as our newly re-sequenced KNG samples, 17 *Capra aegagrus* that is considered as the ancestor of *Capra hircus* and is inhabiting in Iran, 18 Iranian indigenous goats (ING) that have raised in Iran where *C. hircus* was firstly domesticated, 13 Korean crossbred goats (KCB) that have formed by extensive hybridization of KNG wit commercial breeds, 20 Moroccan indigenous goats (MNG) that have raised in Morocco and adapted to Africa's tropical desert climate, 2 Swiss Saanen (SS) and 2 French Saanen (FS) that are used worldwide as dairy breeds, 2 French Alpines (FA) that are used as a dairy breed and distributed throughout France and Switzerland, and 1 Swiss Boer (SB) that is used worldwide as a meat type breed (Additional file 1: Table S2). These goat samples were collected from goat populations, and their brief sampling information is as follows; KNG was sequenced with blood samples extracted from healthy individuals provided at the Animal Genetic Resources Station, National Institute of Animal Science, and Rural Development Administration in Korea (Lee *et al*., 2016); KCB was sequenced with blood samples collected from black goat farms in Korea (Lee *et al*., 2016); MNG was sampled evenly in different areas to represent Morocco as a whole; ING was collected from individuals which are far away as possible from commercial breeds, with consideration in geographical coordinates and phenotypes; *C. aegagrus* was sequenced from hunted samples in Iran and tissues stored at Iranian Conservation Centers; and SB and SS were sampled from healthy individuals raised on farms in Switzerland (Becker *et al*., 2015; Menzi *et al*., 2016). MNG, ING, FA, and *C. aegagrus* were samples related to NextGen's "Goat sequence project" and their detailed information is described in NextGen's "Project final report" (<https://nextgen.epfl.ch/>) (Benjelloun *et al*., 2015; Taberlet, 2015). In this study, *C. aegagrus* and ING were used as reference populations for comparing genomic characteristics of various goat breeds.

- 1. **Identification of SNPs**

The numbers of bi-allelic SNPs in *C. aegagrus* and the indigenous breeds (KNG, ING, and MNG) were much higher than the other commercial breeds (Figure 1B and Additional file 1: Table S5). The number of bi-allelic SNPs of *C. aegagrus*, which is the wild-type goat and considered as the ancestor of *C. hircus*, was the highest at 39,222,625, followed by KNG and ING at 37,715,208 and 35,742,191, respectively. Corresponding to this result, the number of variants identified in intergenic, intron and exon regions were the highest in *C. aegagrus* at 27,299,228, 22,008,256, and 444,958, respectively; followed by KNG at 26,338,911, 21,076,127 and 412,302, respectively. When excluding *C. aegagrus*, missense and silent mutations were the most abundant in KNG, at 188,265 and 224,869, respectively. Although the numbers of nonsense mutations had slight differences among the indigenous breeds (KNG, ING, and MNG), these numbers were higher than the commercial breeds (Additional file 1: Table S6). Interestingly, KCB, which has formed by extensive hybridization of KNG with commercial breeds, showed similar patterns to other indigenous breeds. This indicates that KCB has retained lots of characteristics derived from KNG, suggesting that the purpose of KCB's formation has been successfully accomplished. Meanwhile, we identified differences in the number of bi-allelic SNPs by different habitat environments in four Saanen sub-populations and three Boer sub-populations, respectively. However, we referred to only the overall trends of these populations because some sub-populations (FA, FS, SS, and SB) may have sampling bias due to the low sample sizes.

- 1. **General genomic characteristics**

To obtain a catalog of general genomic characteristics for 15 goat populations comprised to 10 goat breeds, we reviewed nucleotide diversity (π), inbreeding coefficient (F), and linkage disequilibrium (LD) for each population by using all 39,520,457 bi-allelic SNPs (Additional file 1: Tables S4 and S7). The degree of polymorphism for bi-allelic SNPs was measured by π, the intensity of non-random associations for two or more loci was measured by LD, and the proportion of homozygous genotypes was measured by F. These three statistics not only outline the genomic characteristic of a population but also indirectly present bottleneck and founder effects that the population may have experienced in the past. Particularly, the LD presents traces of evolutionary forces such as bottleneck events, selection, geographical subdivisions, and effective population size as a detectable pattern at the genome level, since it is strongly influenced by domestication and subsequent breed formation events (Slatkin, 2008; Bohmanova *et al*., 2010; Kemper and Goddard, 2012; Szpiech *et al*., 2013).

The π value was the highest in ING at 0.001998, followed by in MNG and *C. aegagrus,* at 0.001859 and 0.001804, respectively. Along with this genetic diversity, ING and MNG, which are indigenous breeds, showed higher F values and long-range of low-level LD than other breeds. These results indicate that these indigenous breeds have relatively larger effective population sizes (Figure 3B and Additional file 1: Figures S7A-D) and have experienced low-level selection and genetic drift (Figure 3A). Particularly, the highest diversity of ING indicates that they are the earliest population differentiated from their ancestor, as considering *C. hircus* was first domesticated in Iran (Figures 2A and 3A).

The differences in genomic characteristics along habitats and environments were confirmed within the Saanen breed and the Boer breed, respectively. Even though the π within the same breed should be relatively similar, KS, AS, SS, and FS (Sub-populations of Saanen breed) showed 0.001733, 0.001577, 0.001582, respectively, and 0.001623, and KB, AB, and SB (Sub-populations of Boer breed) showed 0.001554, 0.001727, and 0.001655, respectively. Particularly, KB and AB not only differed in π values but also differed in estimates of average F and average LD values. The average F values of KB and AB were -0.3578 and 0.0387, respectively, and the average LD values were 0.243723 and 0.137452, respectively. These differences imply that these sub-populations have been influenced by artificial or environmental selections in each of the regions. In this study, we only referred to the overall trends of FA, AS, SS, FS, and SB since they may have sampling bias due to the small sample size.

BA and AN showed the lowest π and the negative F values. Also, they showed the highest average LD value among all breeds excepted for AS, SS, and FS (Additional file 1: Figure S1B). These results are presumed to be due to the influence of genetic drift experienced when they have first introduced to Australia and the influence of artificial selection that they have undergone in Australia. Moreover, their LD with long-range and high-level indicate that they could have small effective population sizes. In the KCB, we identified a fairly high π value and low level of LD. It suggests that KCB has lots of new alleles introduced from other commercial breeds.

- 1. **Population differentiation and genic structure**

To identify the genomic relationship of 10 goat breeds, we performed principal component analysis (PCA), phylogenetic tree analysis, structure analysis, and fixation index analysis (Fst). In the reconstructed tree, Saanen and Alpine breeds form a large clade that separates into three sister clades (Figure 2A): "KS and AS", "FS, SS and FA", and "BA." These differentiations were also confirmed in PCA (Figures 2C-D). However, in the structure analysis (Figure. 2B), KS, AS, and BA together formed almost one unique genomic composition (Yellow color), and FS, SS, and FA together formed two genomic compositions (Yellow and green color) at similar proportions. These results imply that KS and AS together have different genomic characteristics from other Saanen populations, and Alpine and Saanen breeds together share a large proportion of their genomic characteristics with each other. The similarity of Alpine and Saanen breeds supports previous reports that they have been close to genetically until a couple of generations ago (Carillier *et al*., 2013; Brito *et al*., 2015). The Alpine breed and the Saanen breed were originated from Alps region of French and Switzerland (Alpine breed) and the Carton Berne region of Switzerland (Saanen breed), respectively. Additionally, they have been bred for the purpose of dairy, so it is presumed that there have been interactions with each other. Meanwhile, the reason why "BA" and "KS and AS" were classified into one subgroup in structure analysis unlike the PCA and the reconstructed tree, is presumed to be due to very varied population stratifications of the 15 goat populations (Figure 2B and Additional file 1: Figure S3). While "FS, SS, and FA" partially showed their ancestral genomic composition (Green color), "KS and AS" showed almost one genomic composition (Yellow color). They are presumed to have experienced genetic drift, new environmental pressures, and artificial selection in each country.

AN is a breed developed in the 1920s through hybridization of British native goat with large lop-eared goats of India, Russia, and Egypt (Jukes, 2017). We confirmed that AN was completely separated from the other 14 goat populations on the third PC accounting for about 5.58% of the total genetic variation (Additional file 1: Figure S4C) and they also had a distinct genomic composition (Red color) from other populations at K = 7 (Figure 2B). Particularly, their genomic compositions showed a higher degree of admixtures than the other breeds. A further research will be needed but AN is presumed to have undergone artificial selections or admixture events for improving the breed’s production capacity after they have experienced a genetic drift while being introduced into Australia.

Boer breed was developed as hybridization with Indian and European breeds, and is an official breed registered in South Africa in 1959 (Casey and Van Niekerk, 1988). KB and AB, therefore, have been only a few decades even since they were introduced into their respective country. Unlike the Saanen breed, which formed three sister clades, the two Boer sub-populations formed a single integrated clade (Figure 2A) and also showed a close relationship with each other in the structure analysis (Figure 2B); however, they tended to be slightly separated from each other in the PCA (Figures 2C-D). This minor difference indicates that each sub-population has undergone different environmental selections, artificial selections or genetic drifts during a short period of time after being introduced to each country. Meanwhile, Boer breed showed the same genomic composition (Green color) as MNG in the structure analysis but showed a noticeable difference with MNG in the PCA. These results imply that their genome is different for each other but have a common genomic background derived from Africa partially (Figure 3A).

ING was positioned at the center of 15 goat populations on the PCA and showed a linear trend with C. aegagrus (Figure 2C). In the structure analysis, *C. aegagrus* showed the unique genomic composition (Pink color) and ING's genomic composition (Green color) with various degrees of admixture (Figure 2B). This indicates that *C. aegagrus*, which is a largely unmanaged feral goat, have been interacting with ING to date since they were domesticated in the Iranian region (Figure 3A). Meanwhile, a previous study has reported that the Iranian indigenous goats living in the north of the Zagros mountain had the most similar genomic structure to their ancestor, *C. aegagrus*. (Vahidi *et al*., 2014). In this study, we confirmed that ING's genome is almost identical to the genomic structure of the Iranian indigenous goats living in the north of the Zagros mountain. This provided reliability to our results and samples.

KCB was recently formed by extensive hybridization of several commercial breeds with KNG to improve the inferior traits of KNG (Lee *et al*., 2016). Therefore, KCB inherited the genomic characteristic of KNG. KCB shared a significant amount of genomic composition with KNG in structure analysis (Figure 2B) and also showed the similar genomic characteristic to KNG in PCA (Figures 2C-D). In the PCA and the reconstructed tree, individuals of KCB showed the spreading tendency, which indicates that their genetic diversity and their genomic characteristics have not yet stabilized.

- 1. **Gene flow and demographic history**

To better visualize genetic drift and gene flows of 10 goat breeds, we reconstructed the maximum likelihood tree using TreeMix v1.13 (Pickrell and Pritchard, 2012) (Figure 3A). The TreeMix presents the degree of genetic drift and the directions of gene flows existing among the populations by modeling the splits and mixing of populations. This method distinguishes from the structure analysis that cannot address the direction of genetic exchange. To further validate migration edges (gene flows) deduced from the reconstructed tree, we conducted to the Patterson's D-statistic test (Durand *et al*., 2011) and the 3-Population test (Reich *et al*., 2009) using Admixtools v4.1 (Patterson *et al*., 2012). These genomic methods are able to quantitatively estimate the relationship of populations. Particularly, the D-statistic can provide clear evidence although the gene flow events may have occurred hundreds of years ago (Green *et al*., 2010; Pickrell and Pritchard, 2012).

In the maximum likelihood tree, we found that ING, MNG, and *C. aegagrus* have undergone gene flows each other (Figure 3A) C. aegagrus's genome was derived from ING's genome by about 33%. MNG's genome was derived from the genomes of at least more than one population which associated with Saanen breeds and C. aegagrus, by about 57%. C. aegagrus is presumed to have been affected by ING which is being raised as a traditional pastoralism system (Figure 2C). Also, MNG is presumed to have been hybridized with breeds such as Saanen or Alpine to improve their dairy ability, as previously reported that they had undergone intermediate-level of hybridization (Benjelloun *et al*., 2015). Similar to MNG, the ING which has been influenced by MNG's genome by about 10% is also presumed to have undergone hybridization to improve their dairy ability (Vahidi *et al*., 2014).

AN and KCB were identified that they affected about 20% of the genomes of KS, AS, and BA; and KNG was identified that they affected about 24% of the genome of KCB. It is presumed that KCB and AN have been hybridized with KS, AS, and BA to improve their dairy ability. Particularly, KCB genome well reflects their own breed formation history which formed by extensive hybridization of various commercial breeds with KNG. Interestingly, we could not find any evidence that KNG has interacted with other populations except KCB. This was also not found in additional verifications through Patterson's D-statistic test and the 3-Population test (Additional file 2).

The X-axis of the TreeMix, which represents the intensity of the genetic drift, depends on the past effective population size (Ne). The Ne plays a key role in exploring the demographic history of a population because it is a primary factor of genetic drift, inbreeding, and genetic diversity (Ewens, 1990; Ballou *et al*., 2010; Frankham *et al*., 2010; Gasca-Pineda *et al*., 2013). Therefore, we inferred the Ne of each population in order to better understand the history of our goat populations, using the PopSizeABC (Boitard *et al*., 2016). Unlike pairwise sequentially Markovian coalescent (PSMC) and sequentially Markovian coalescent (SMC) methods focusing on the full likelihood of the sampled genome, this new approximate Bayesian computation method (PopSizeABC) focuses on the folded allele frequency spectrum, the average zygotic linkage disequilibrium (LD), and the identity by state (IBS). Thus, this method increased the speed and efficiency of the calculation and was suitable for our case investigating whole genome variation (Figure 3B and Additional file 1: Table S9).

The most recent Ne of AN, BA, AB, AS, KS and KCB were 140, 120, 166, 231, 344 and 149, respectively (Additional file 1: Table S9). These breeds have suffered from strong population declines. These populations, which were recently introduced into Australia and Korea, are presumed to be still adapting to their new environment. Interestingly, KB did not show a tendency for Ne to decrease, unlike AB. Although KB and AB were introduced at similar times in each country (Son, 1999), KB showed a considerably different Ne from AB (Figure 3B) and formed a cluster distinguished from AB in the PCA (Figures 2C-D). The KB, which has experienced a relatively strong genetic drift unlike AB (Figure 3A), is presumed that they were initially suffered from its new environmental condition but was soon artificially or naturally adapted to Korea. On the other hand, AB is presumed that they have not adapted well to several evolutionary forces or is still adapting to its new environment. The AN, which is using for various purposes such as dairy and meat, showed a pattern in which Ne increased slightly and then decreased again, unlike other Australian populations (Additional file 1: Figure S7D). This pattern supports the result of Figure 3A and it suggests that AN suffered from a strong genetic drift as they entered Australia. The BA, which also showed a rather strong genetic drift (Figure 3A), is presumed to have undergone a genetic drift similar to AN. Meanwhile, the low Ne of KCB reflects their breed formation history, and it indicates that the KNG's hybridization has largely been successful (Waples and England, 2011; Gasca-Pineda *et al*., 2013).

The estimates of Ne for 14 goat populations are summarized in Additional file 1: Table S9, and 90% credible intervals of the inferred Ne are shown in Additional file 1: Figure S8. In the analysis of the Ne and the maximum likelihood tree, SS, FS, and FA were only referred to their general tendencies because there may be a bias due to low sampling size.

**S2: Supplementary References**

Benjelloun, B., Alberto, F. J., Streeter, I., Boyer, F., Coissac, E., Stucki, S., et al. (2015). Characterizing neutral genomic diversity and selection signatures in indigenous populations of Moroccan goats (Capra hircus) using WGS data. *Front. Genet.* 6, 107. doi: 10.3389/fgene.2015.00107

Ballou, J. D., Lees, C., Faust, L. J., Long, S., Lynch, C., Bingaman Lackey, L., et al. (2010). “Demographic and genetic management of captive populations,” in *Wild mammals in captivity: principles and techniques for zoo management*, eds. D. G. Kleiman, K. V. Thompson, and C. K. Baer (Chicago, FL: The University of Chicago Press), 219-252.

Becker, D., Otto, M., Ammann, P., Keller, I., Drögemüller, C., and Leeb, T. (2015). The brown coat colour of Coppernecked goats is associated with a non‐synonymous variant at the TYRP1 locus on chromosome 8. *Anim. Genet.* 46(1), 50-54. doi: 10.1111/age.12240

Bohmanova, J., Sargolzaei, M., and Schenkel, F. S. (2010). Characteristics of linkage disequilibrium in North American Holsteins. *BMC Genomics* 11, 421.  doi: 10.1186/1471-2164-11-421

Boitard, S., Rodríguez, W., Jay, F., Mona, S., and Austerlitz, F. (2016). Inferring population size history from large samples of genome-wide molecular data-an approximate Bayesian computation approach. *PLoS Genet.* 12(3), e1005877. doi: 10.1371/journal.pgen.1005877

Brito, L. F., Jafarikia, M., Grossi, D. A., Kijas, J. W., Porto-Neto, L. R, Ventura, R. V., et al. (2015). Characterization of linkage disequilibrium, consistency of gametic phase and admixture in Australian and Canadian goats. *BMC Genet.* 16, 67. doi: 10.1186/s12863-015-0220-1

Carillier, C., Larroque, H., Palhière, I., Clément, V., Rupp, R., and Robert-Granié, C. (2013). A first step toward genomic selection in the multi-breed French dairy goat population. *J. Dairy Sci.* 96(11), 7294-7305. doi: 10.3168/jds.2013-6789

Casey, N., and Van Niekerk, W. (1988). The Boer goat. I. Origin, adaptability, performance testing, reproduction and milk production. *Small Rumin. Res.* 1(3), 291-302. doi: 10.1016/0921-4488(88)90056-9

Durand, EY., Patterson, N., Reich, D., and Slatkin, M. (2011). Testing for ancient admixture between closely related populations. *Mol. Biol. Evol.* 28(8), 2239-2252. doi: 10.1093/molbev/msr048

Ewens, W. (1990). “The minimum viable population size as a genetic and a demographic concept,” in *Convergent issues in genetics and demography*, eds. J. Adams, D. A. Lam, A. I. Hermalin, and, P. E. Smouse (New York, FL: Oxford University Press), 307-316.

Frankham, R., Ballou, J. D., and Briscoe, D. A. (2010). Introduction to conservation genetics. (Second edition). Cambridge: Cambridge University Press.

Gasca-Pineda, J., Cassaigne, I., Alonso, R. A., and Eguiarte, L. E. (2013). Effective population size, genetic variation, and their relevance for conservation: the bighorn sheep in Tiburon Island and comparisons with managed artiodactyls. *PLoS One* 8, e78120. doi: 10.1371/journal.pone.0078120

Green, R. E., Krause, J., Briggs, A.W., Maricic, T., Stenzel, U., Kircher, M., et al. (2010). A draft sequence of the Neandertal genome. *Science* 328(5979), 710-722. doi: 10.1126/science.1188021

Jukes, E. (2017). Mason’s World Encyclopedia of Livestock Breeds and Breeding. *Reference Reviews.* 31(5), 28-30. doi: 10.1108/RR-02-2017-0039

Kemper, K. E., and Goddard, M. E. (2012). Understanding and predicting complex traits: knowledge from cattle. *Hum. Mol. Genet.* 21(R1), R45-R51. doi: 10.1093/hmg/dds332

Lee, W., Ahn, S., Taye, M., Sung, S., Lee, H.-J., Cho, S., et al. (2016). Detecting Positive Selection of Korean Native Goat Populations Using Next-Generation Sequencing. *Mol. Cells* 39(12), 862. doi: 10.14348/molcells.2016.0219

Menzi, F., Keller, I., Reber, I., Beck, J., Brenig, B., Schütz, E., et al. (2016). Genomic amplification of the caprine EDNRA locus might lead to a dose dependent loss of pigmentation. *Sci. Rep.* 6, 28438. doi: 10.1038/srep28438

Patterson, N., Moorjani, P., Luo, Y., Mallick, S., Rohland, N., Zhan, Y., et al. (2012). Ancient admixture in human history. *Genetics* 192, 1065-1093. doi: 10.1534/genetics.112.145037

Pickrell, J. K., and Pritchard, J. K. (2012). Inference of population splits and mixtures from genome-wide allele frequency data. *PLoS Genet.* 8(11), e1002967. doi: 10.1371/journal.pgen.1002967

Reich, D., Thangaraj, K., Patterson, N., Price, A. L., and Singh, L. (2009). Reconstructing Indian population history. *Nature* 461(7263), 489-494. doi: 10.1038/nature08365

Slatkin, M. (2008). Linkage disequilibrium—understanding the evolutionary past and mapping the medical future. *Nat. Rev. Genet.* 9(6), 477-485. doi: 10.1038/nrg2361

Son, Y. S. (1999). Production and uses of Korean Native Black Goat. *Small Rumin. Res.* 34(3), 303-308. doi: 10.1016/S0921-4488(99)00081-4.

Szpiech, Z. A., Xu, J., Pemberton, T. J., Peng, W., Zöllner, S., Rosenberg, N. A., et al. (2013). Long runs of homozygosity are enriched for deleterious variation. *Am. J. Hum. Genet.* 93(1), 90-102. doi: 10.1016/j.ajhg.2013.05.003

Taberlet, P. (2015). PROJECT FINAL REPORT: Next generation methods to preserve farm animal biodiversity by optimizing present and future breeding options. *NEXTGEN*. Available online at: https://cordis.europa.eu/docs/results/244/244356/final1-nextgen-final-report-12092014-v2.pdf

Vahidi, S. M. F., Tarang, A. R., Anbaran, M. F., Boettcher, P., Joost, S., Colli, L., et al. (2014). Investigation of the genetic diversity of domestic Capra hircus breeds reared within an early goat domestication area in Iran. *Genet. Sel. Evol.* 46, 27. doi: 10.1186/1297-9686-46-27

Waples, R. S., and England, P. R. (2011). Estimating contemporary effective population size on the basis of linkage disequilibrium in the face of migration. *Genetics* 189(2), 633-644. doi: 10.1534/genetics.111.132233

**S3: Supplementary Figures S1-S16**

**Figure S1.** The extent of the LD decay for 14 goat populations. **(A)** shows *C. aegagrus*, Korean crossbred, and indigenous breeds (Iranian indigenous goat, Moroccan indigenous goat, and Korean indigenous goat), **(B)** shows Anglo-Nubian, British-Alpine, and Alpine, **(C)** shows Saanen breeds containing four sub-breeds (Swiss Saanen, Australian Saanen, French Saanen, and Korean Saanen), and **(D)** shows Boer breeds containing two sub-breeds (Korean Boer and Australian Boer). For comparison, Korean indigenous goat is included in all plots.


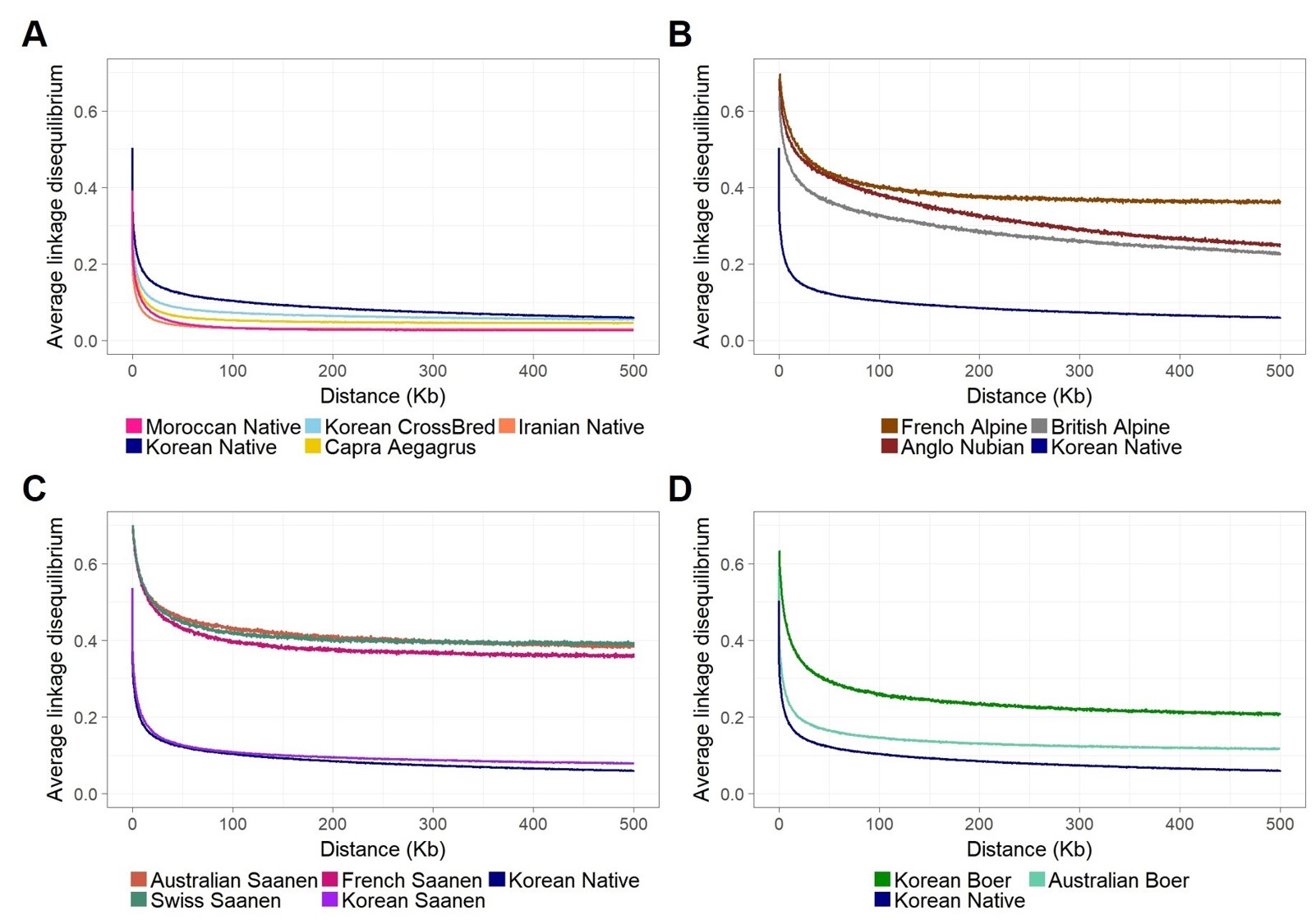


**Figure S2.** The pairwise relationship for identical by state of 136 goat samples. *C. aegagrus* and *C. hircus* tend to be differentiated, and Korean indigenous goat show the tendency to be strongly grouped.


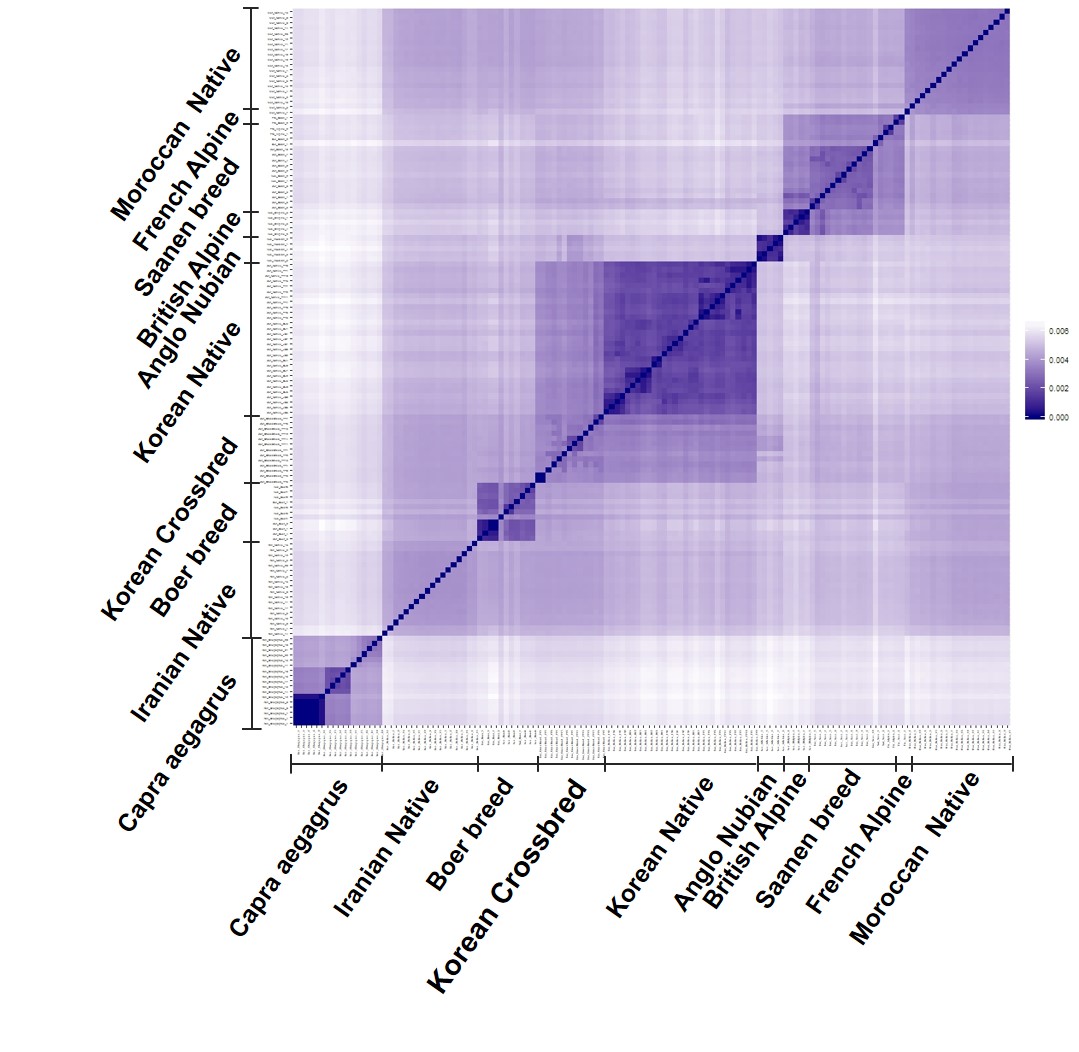


**Figure S3.** The genomic structure for K = 4, 6, 8, and 10 calculated by the clustering algorithm. Korean indigenous goat and Anglo-Nubian forms a unique genomic composition, respectively.


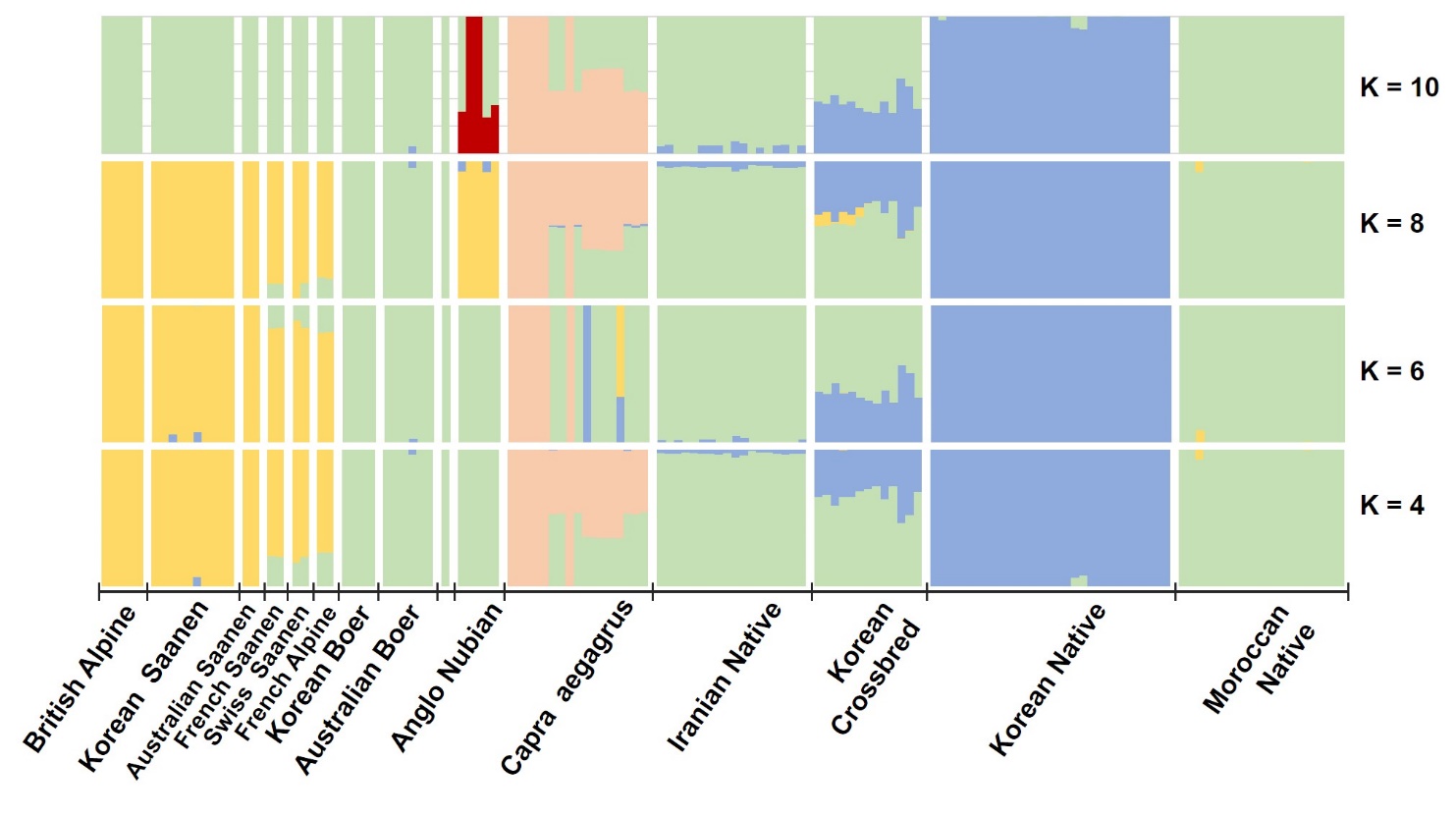


**Figure S4.** Relationship of 15 goat populations. **(A), (B)** The principal component analysis and its scree plot visualized with PC2 and PC3 of Figure 2C including *C. aegagrus*. **(C), (D)** The principal component analysis and its scree plot visualized with PC2 and PC3 of Figure 2D excluding *C. aegagrus*.


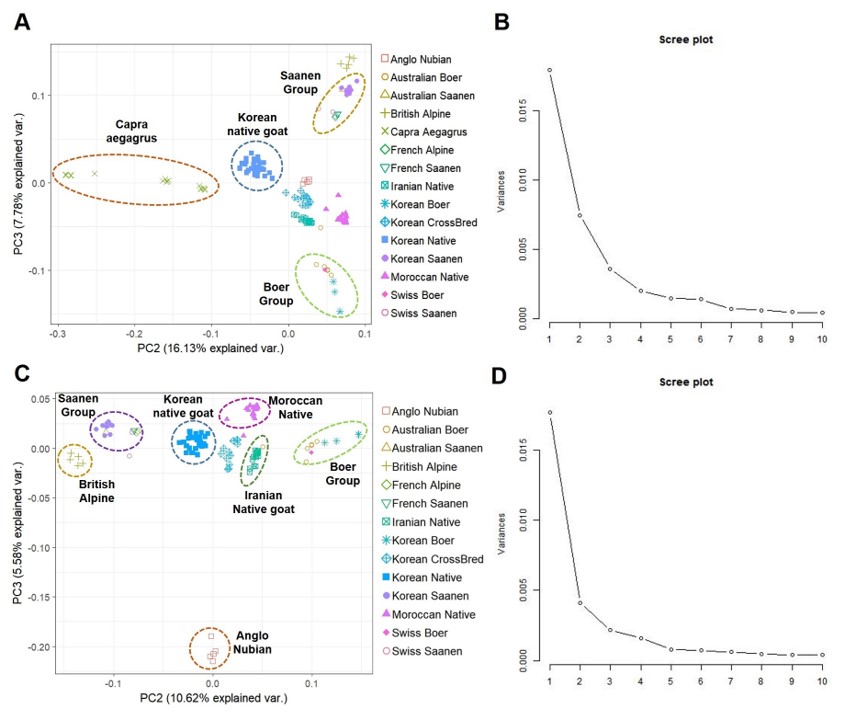


**Figure S5.** The residual matrix of the maximum-likelihood tree of Figure 3A. The residuals above zero represent closely related population pair.


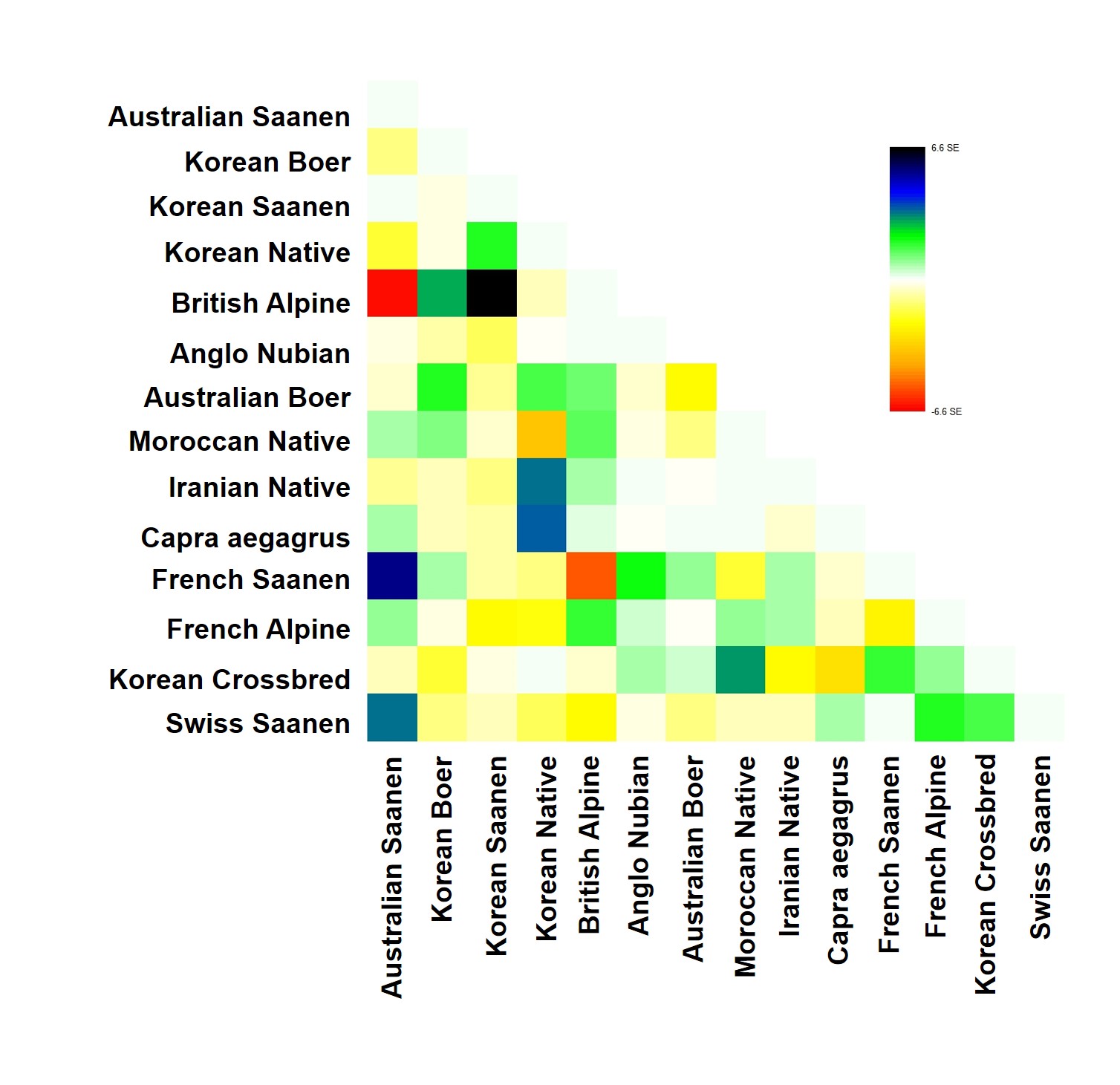


**Figure S6.** Other maximum-likelihood trees derived from Figure 3A. **(A), (B)** Patterns of each tree are very similar to Figure. 3A. As the standard errors of the inferred trees are small, the branches and the migration edges of each plot show little variation. The residual matrix plots for each tree is illustrated to the right of them, respectively. These residual matrices are also very similar to Figure S5 which is the residual matrix of Figure 3A.

**
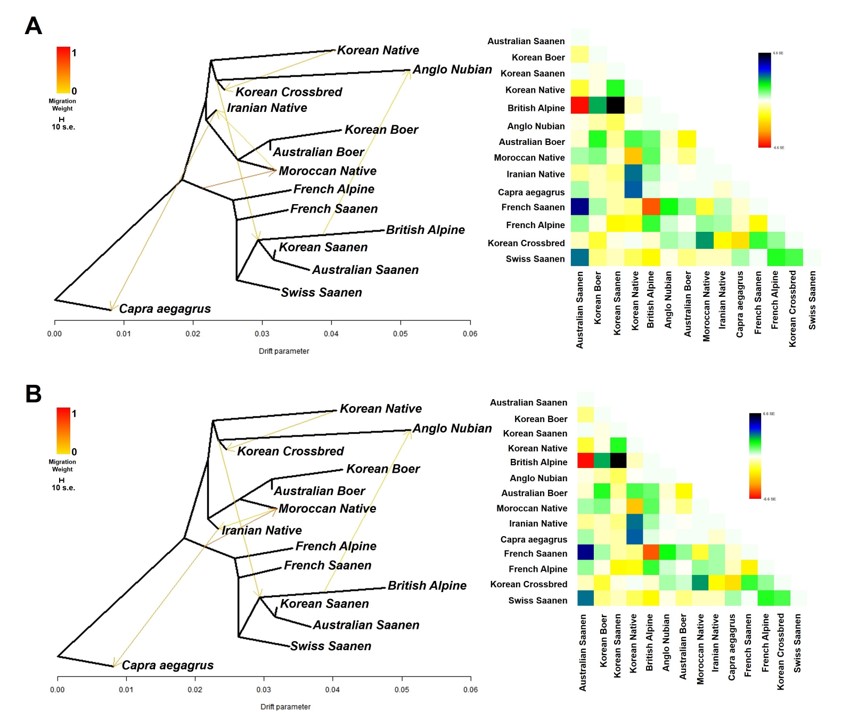
**

**Figure S7.** Inferred demographic history for 14 goat populations. **(A)** shows *C. aegagrus*, Korean crossbred, and indigenous breeds (Iranian indigenous goat, Moroccan indigenous goat, and Korean indigenous goat), **(B)** shows Boer breeds (Korean Boer and Australian Boer), **(C)** shows Saanen Breeds (Swiss Saanen, French Saanen, Australian Saanen, and Korean Saanen), and **(D)** shows Anglo-Nubian, British-Alpine, and Alpine. For comparison, Korean indigenous goat is included in all plots (See Additional file 1: Table S9).

**
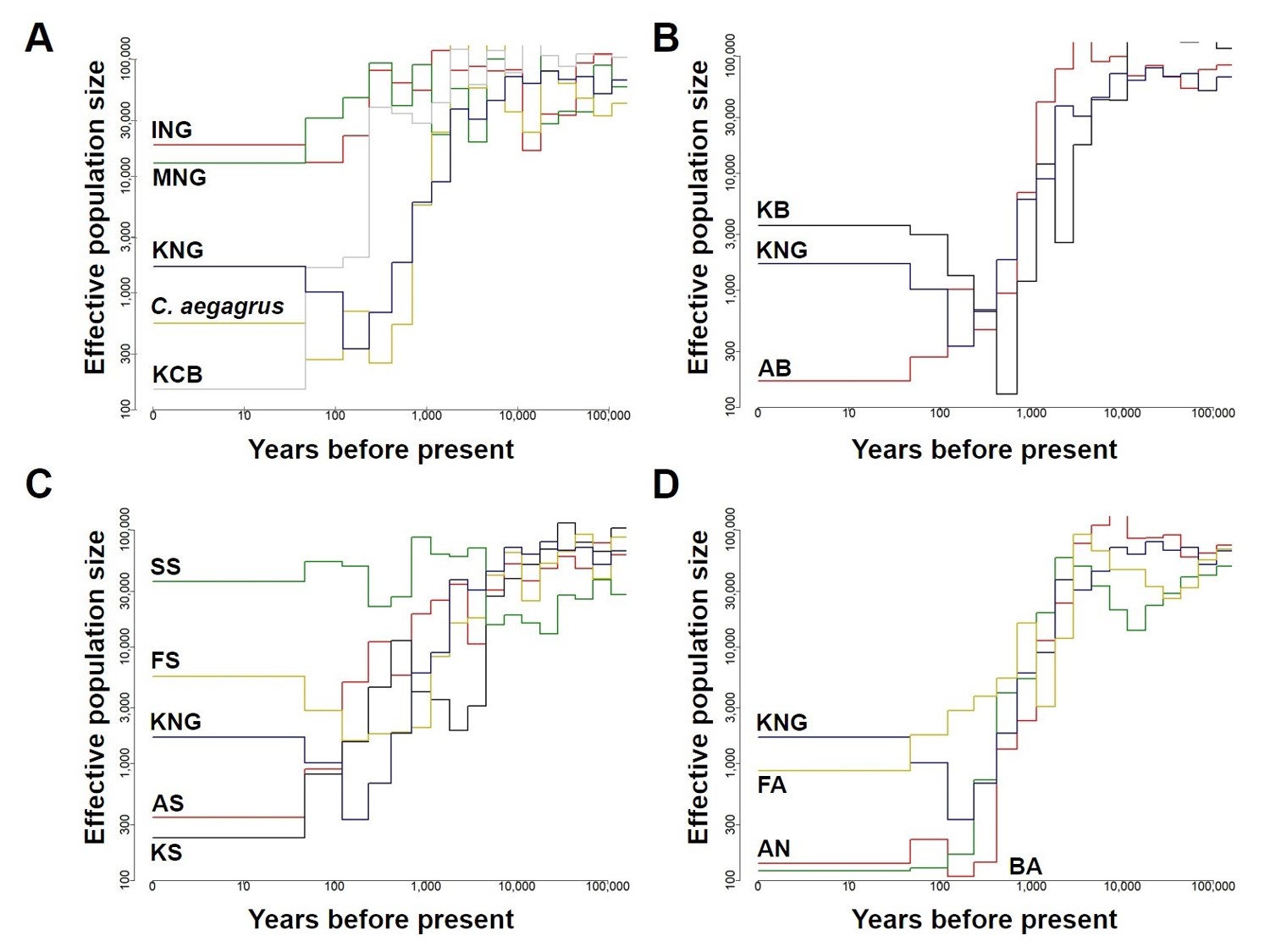
**

**Figure S8.** The estimated effective population size with the credible intervals for 14 goat population. The 90% credible intervals were marked as a dotted line. The unit of the X-axis is years from the present to the past, and the unit of Y-axis is the number of effective population size.


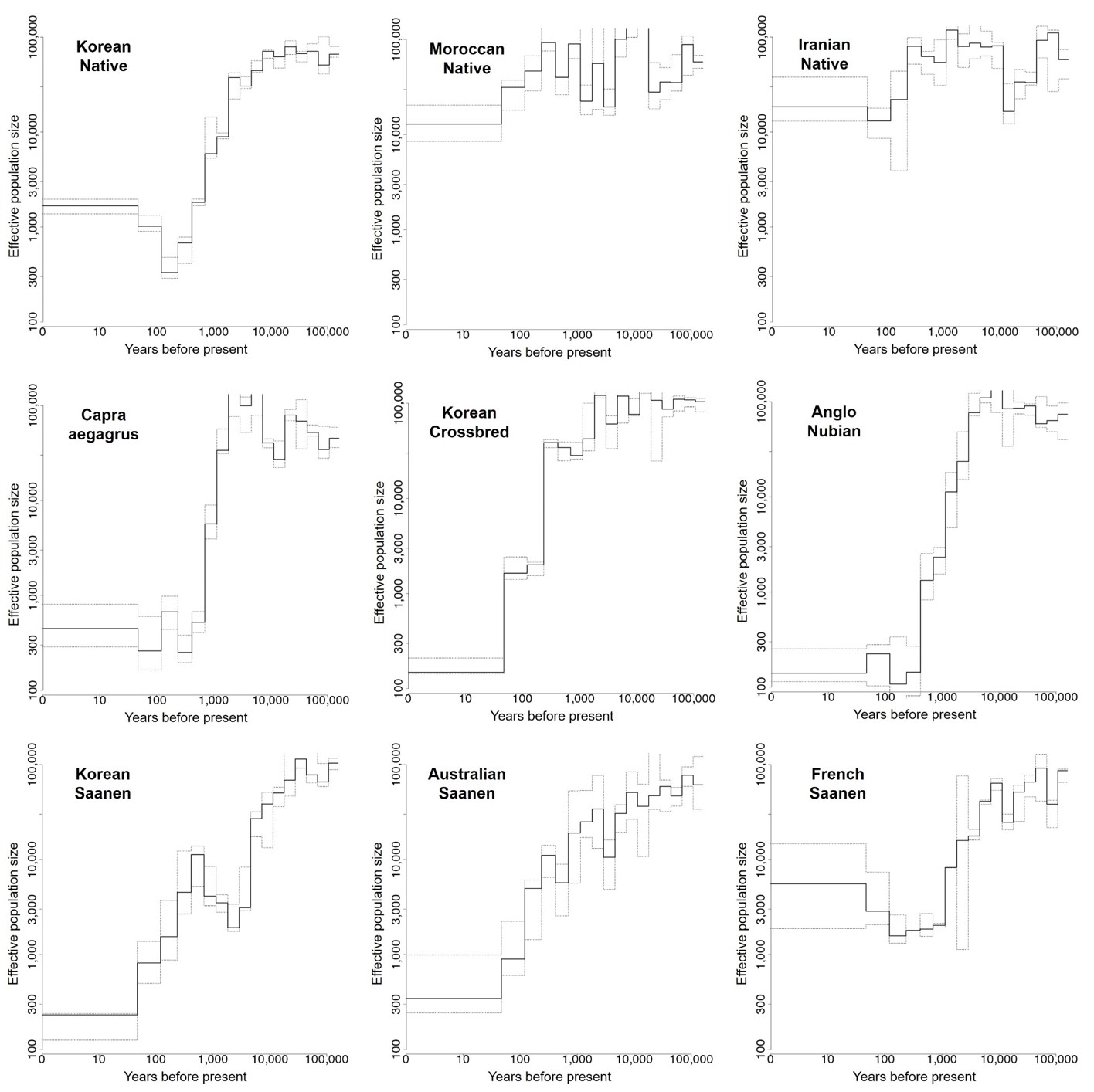


**Figure S8 (cont.).** The estimated effective population size with the credible intervals for 14 goat population. The 90% credible intervals were marked as a dotted line. The unit of the X-axis is years from the present to the past, and the unit of Y-axis is the number of effective population size.


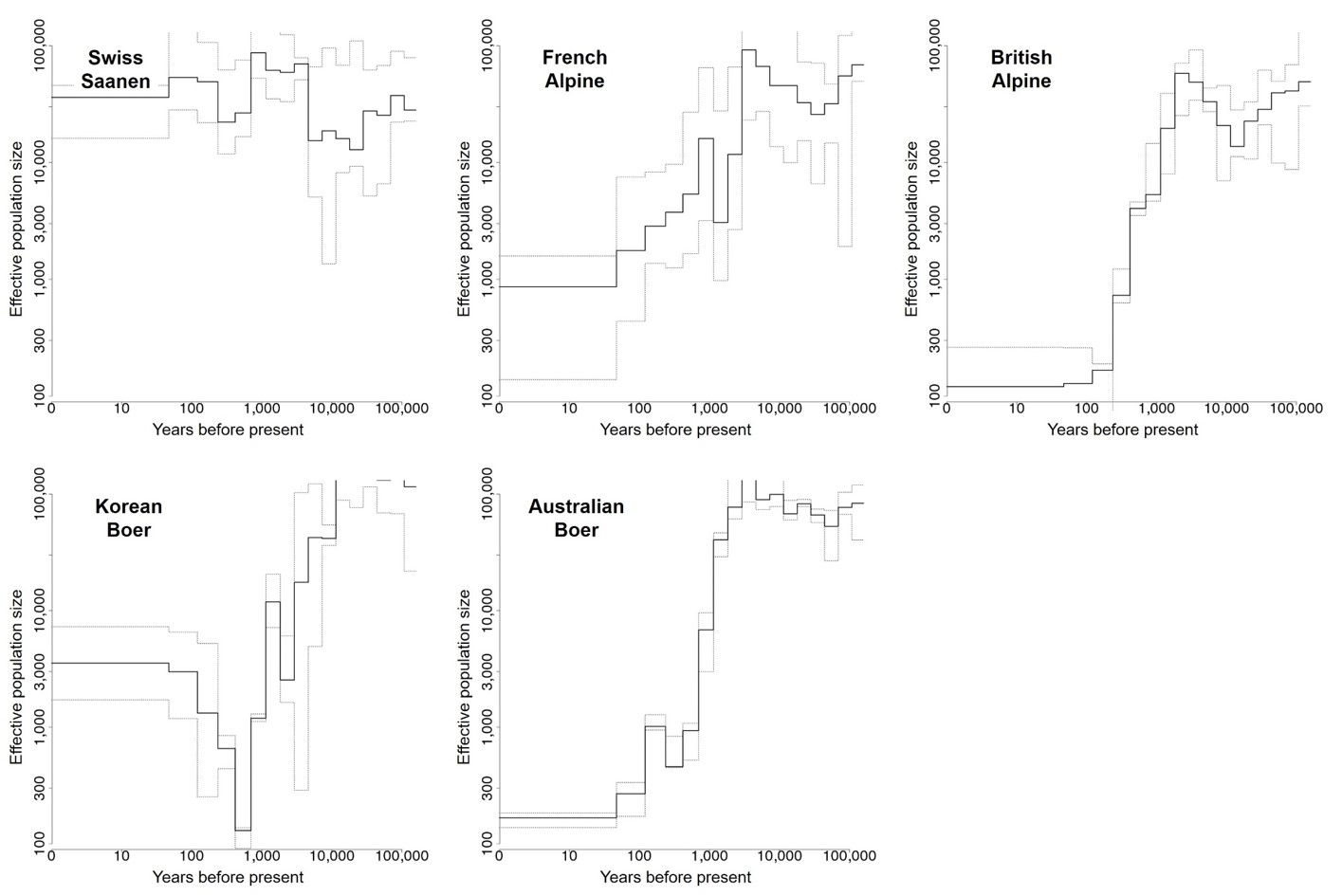


**Figure S9.** The distributions of the XP-EHH raw score for 10 goat populations. Each plot contains the population name compared with Korean indigenous goat.

**
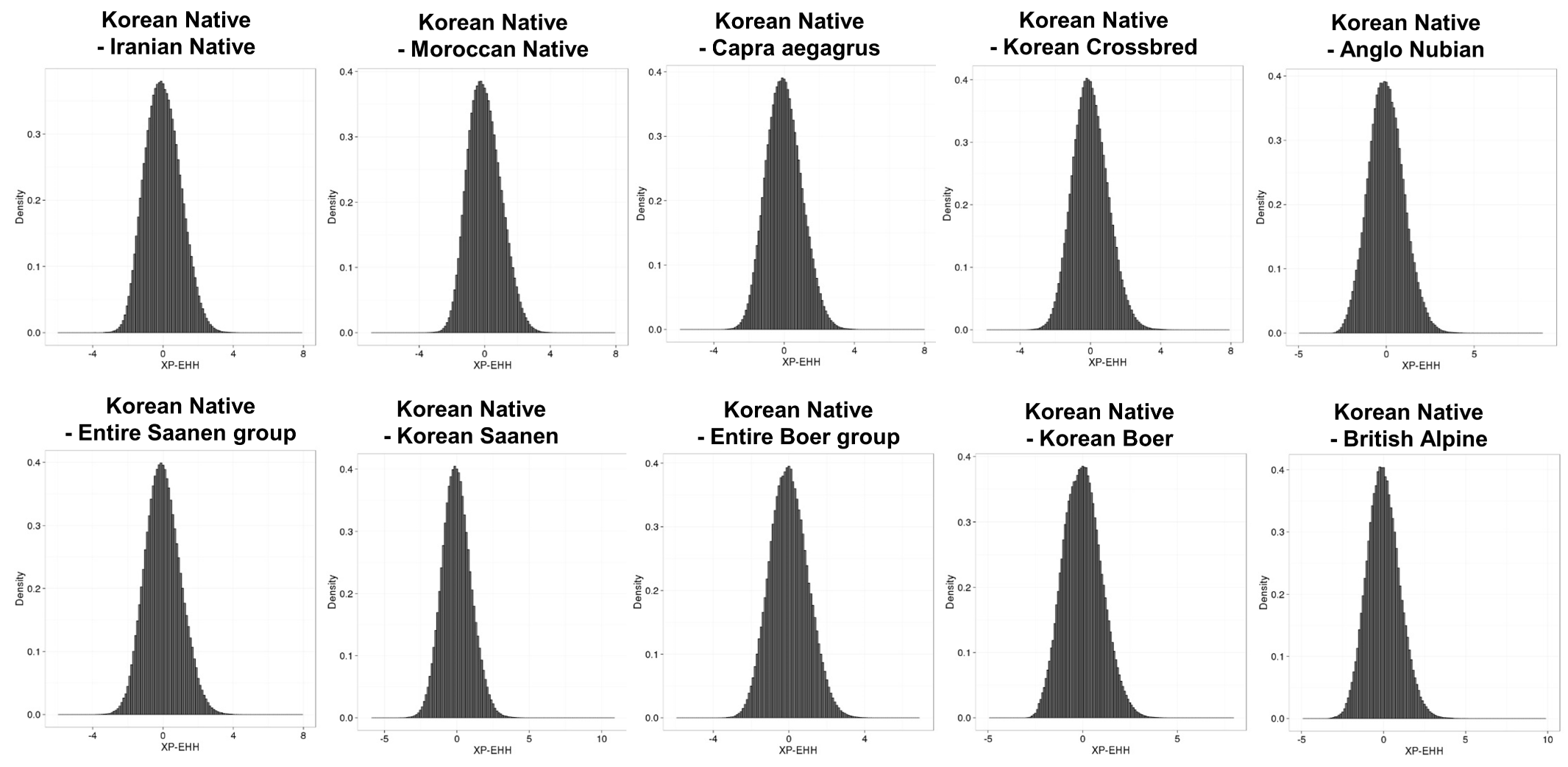
**

**Figure S10.** The distributions of the XP-CLR raw score for 10 goat populations. Each plot contains the population name compared with Korean indigenous goat.


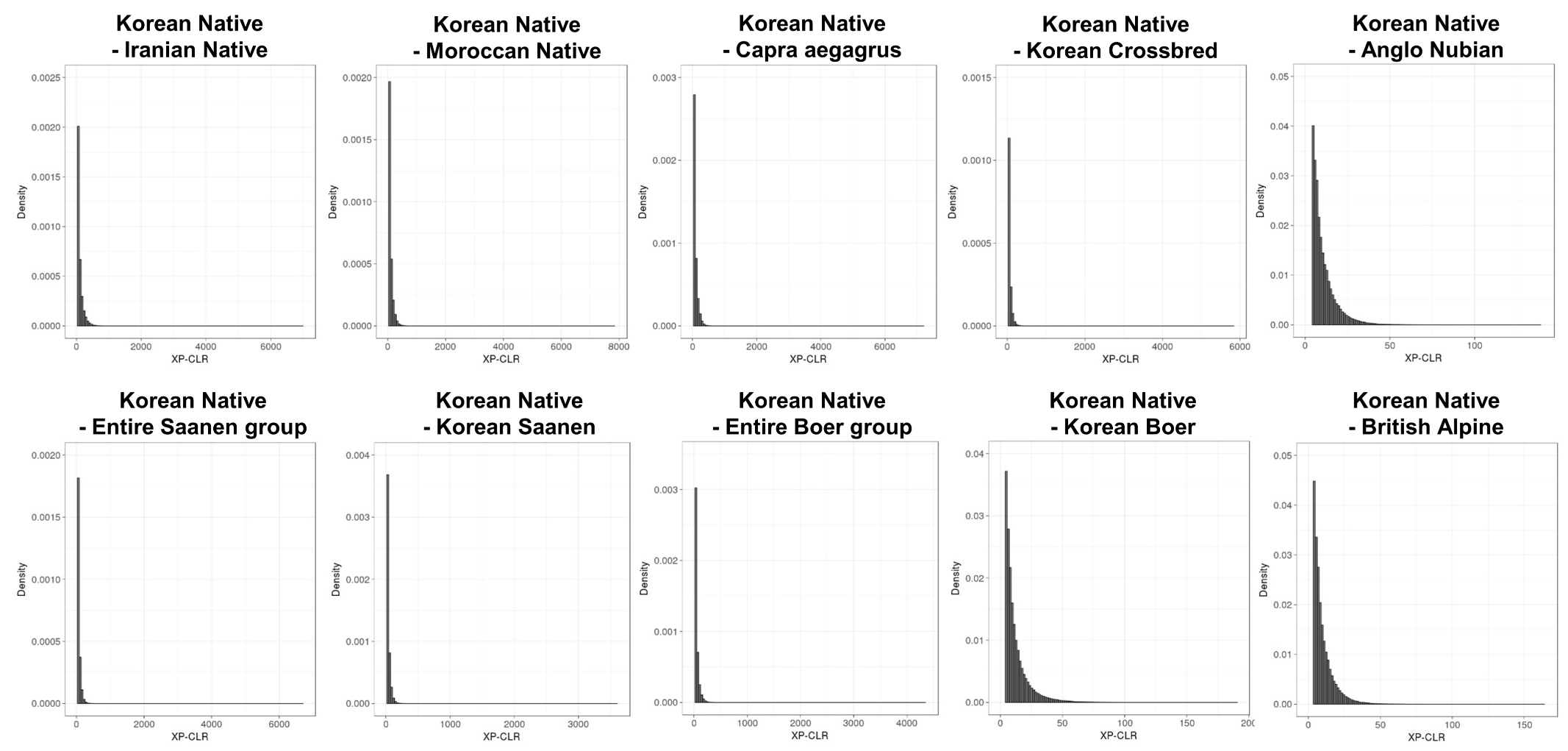


**Figure S11.** Manhattan plots resulted from the selection analysis compared Korean indigenous goat to **(A)** Iranian indigenous goat, **(B)** Moroccan indigenous goat, **(C)** *C. aegagrus*, **(D)** Korean Boer, **(E)** entire Boer group, **(F)** Korean Saanen, **(G)** entire Saanen group, **(H)** Anglo-Nubian, **(I)** British-Alpine, and **(J)** Korean crossbred, respectively. The left and right sides show the results calculated by the XP-CLR and the XP-EHH, respectively. The cut-off line for the top 0.1% outlier is indicated by a red dotted line.


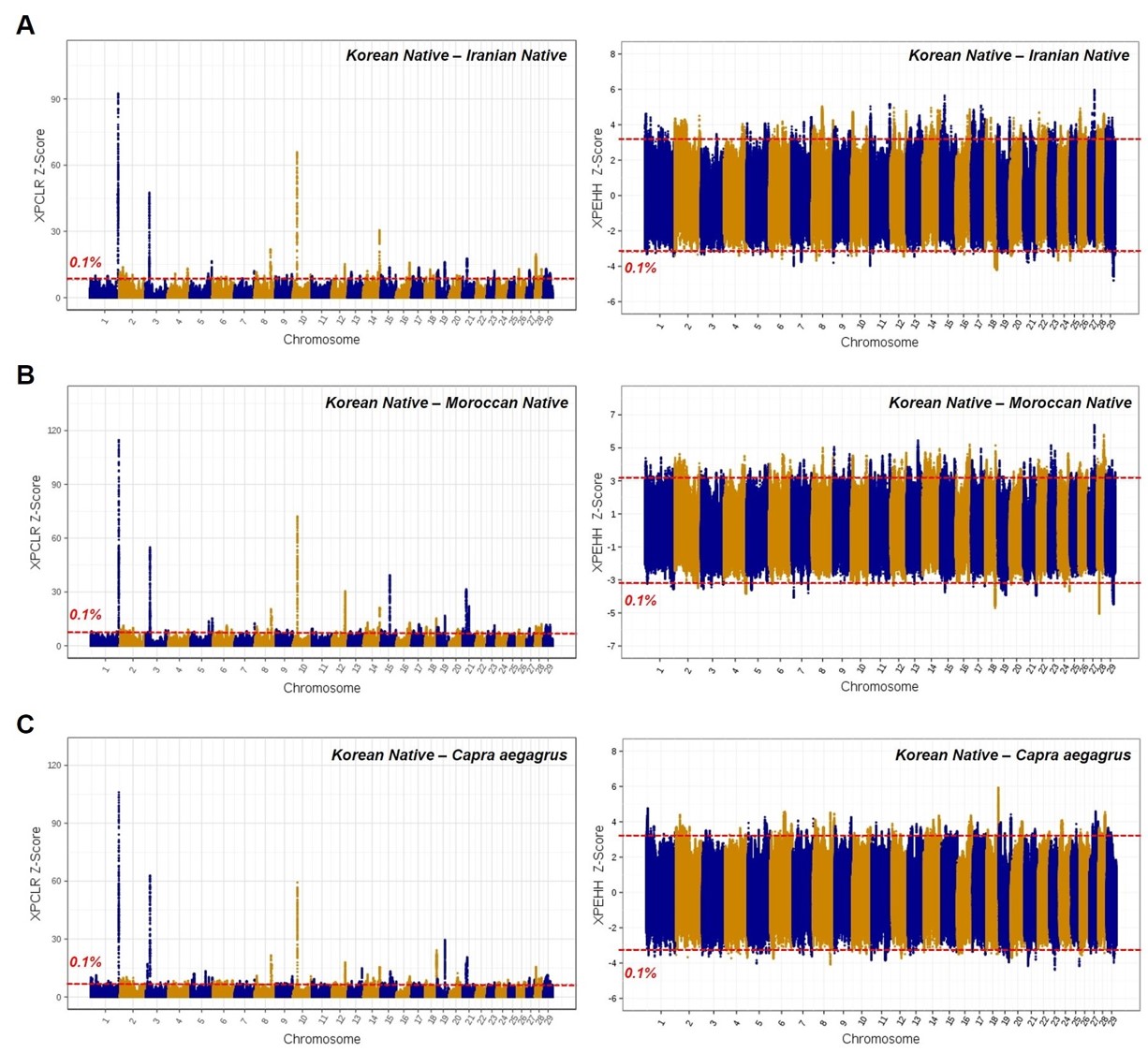


**Figure S11 (cont.).** Manhattan plots resulted from the selection analysis compared Korean indigenous goat to **(A)** Iranian indigenous goat, **(B)** Moroccan indigenous goat, **(C)** *C. aegagrus*, **(D)** Korean Boer, **(E)** entire Boer group, **(F)** Korean Saanen, **(G)** entire Saanen group, **(H)** Anglo-Nubian, **(I)** British-Alpine, and **(J)** Korean crossbred, respectively. The left and right sides show the results calculated by the XP-CLR and the XP-EHH, respectively. The cut-off line for the top 0.1% outlier is indicated by a red dotted line.


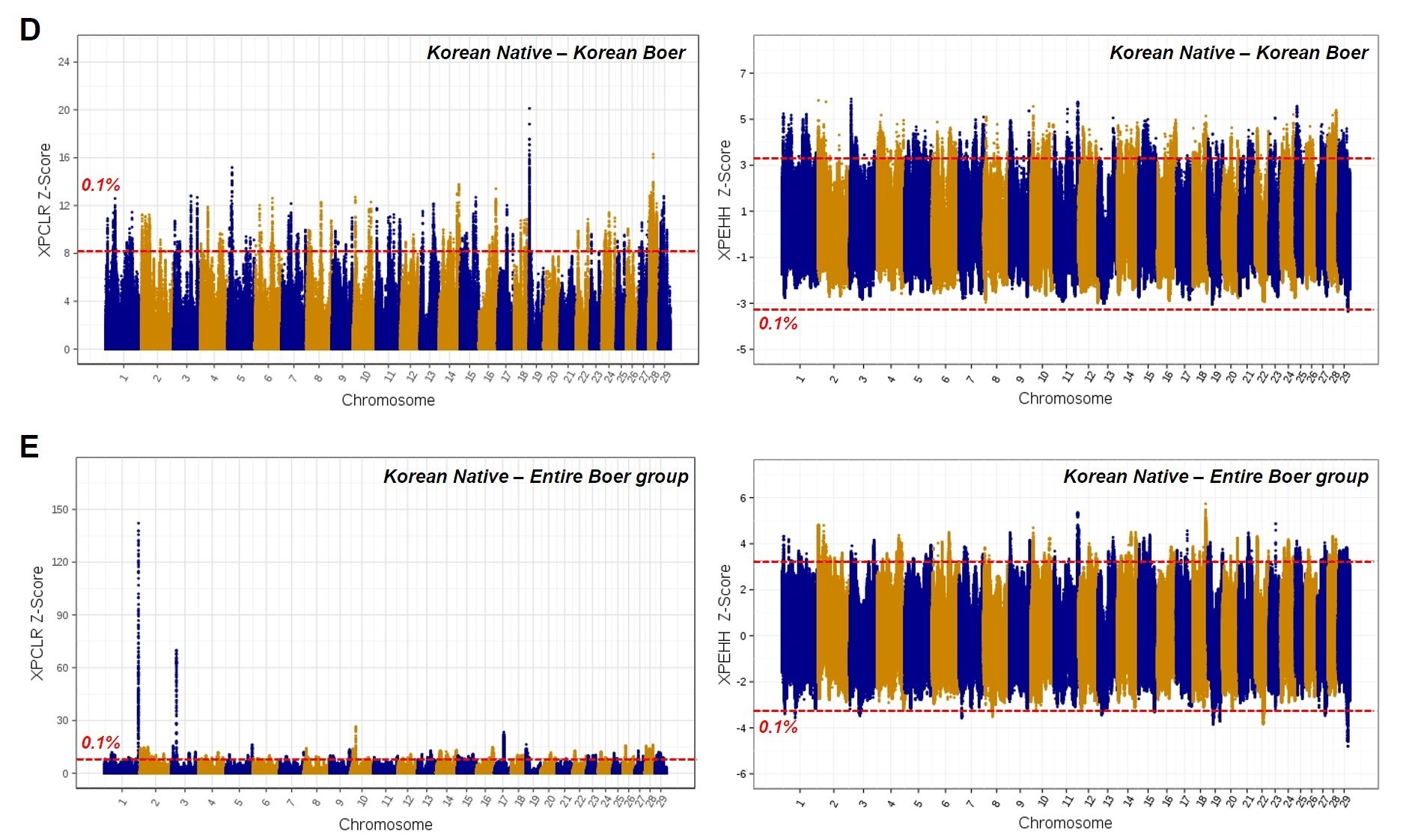


**Figure S11 (cont.).** Manhattan plots resulted from the selection analysis compared Korean indigenous goat to **(A)** Iranian indigenous goat, **(B)** Moroccan indigenous goat, **(C)** *C. aegagrus*, **(D)** Korean Boer, **(E)** entire Boer group, **(F)** Korean Saanen, **(G)** entire Saanen group, **(H)** Anglo-Nubian, **(I)** British-Alpine, and **(J)** Korean crossbred, respectively. The left and right sides show the results calculated by the XP-CLR and the XP-EHH, respectively. The cut-off line for the top 0.1% outlier is indicated by a red dotted line.


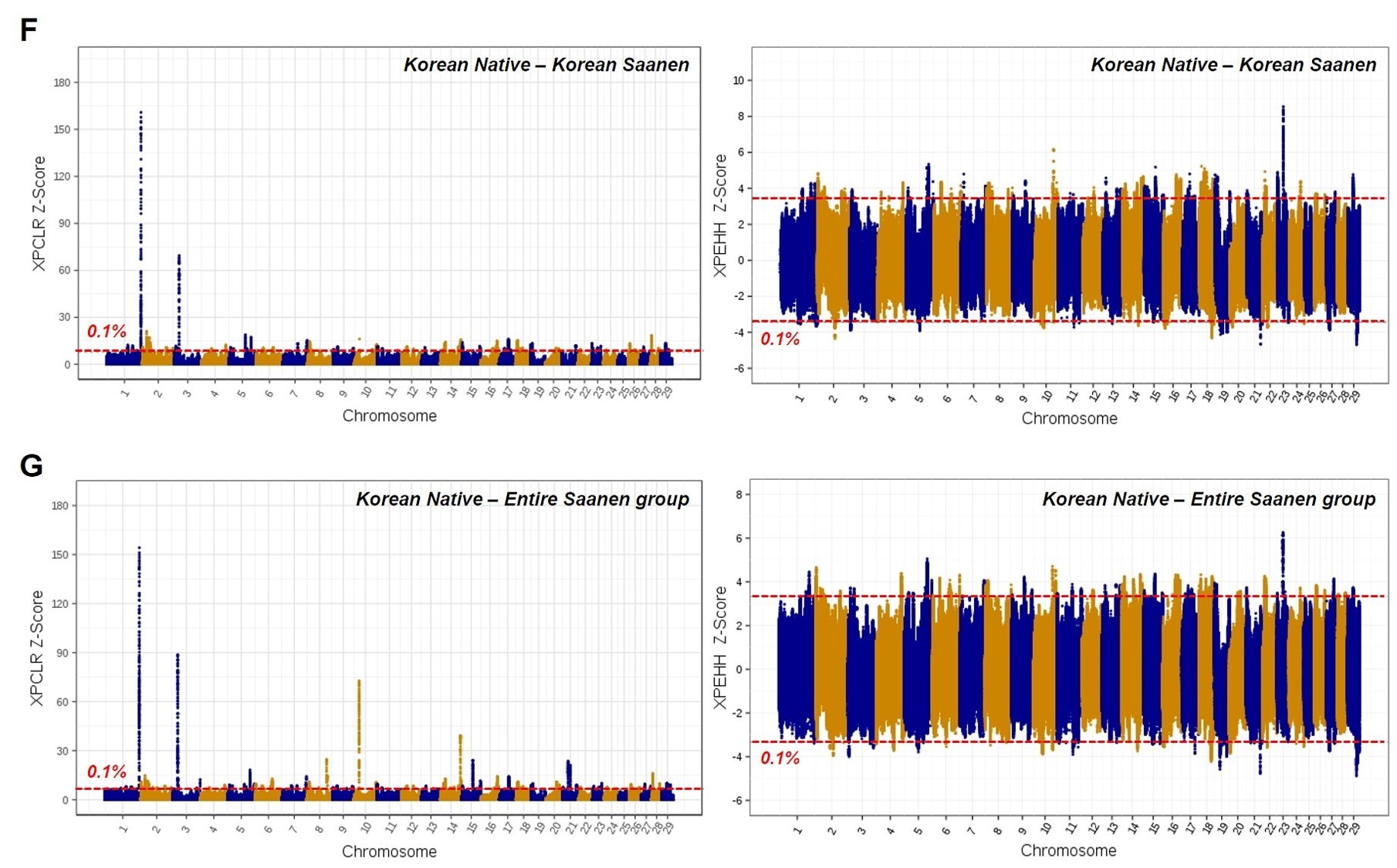


**Figure S11 (cont.).** Manhattan plots resulted from the selection analysis compared Korean indigenous goat to **(A)** Iranian indigenous goat, **(B)** Moroccan indigenous goat, **(C)** *C. aegagrus*, **(D)** Korean Boer, **(E)** entire Boer group, **(F)** Korean Saanen, **(G)** entire Saanen group, **(H)** Anglo-Nubian, **(I)** British-Alpine, and **(J)** Korean crossbred, respectively. The left and right sides show the results calculated by the XP-CLR and the XP-EHH, respectively. The cut-off line for the top 0.1% outlier is indicated by a red dotted line.


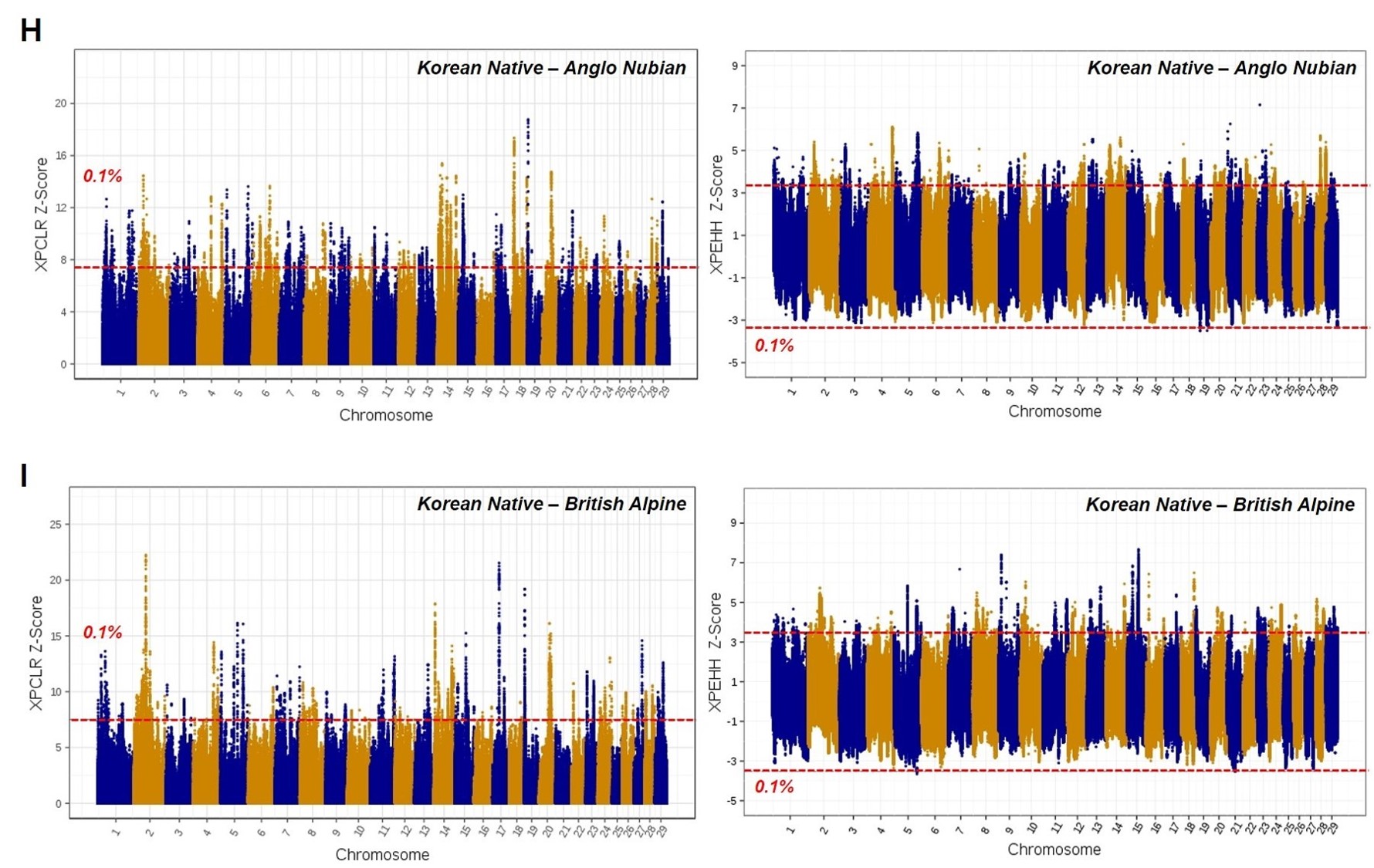
**
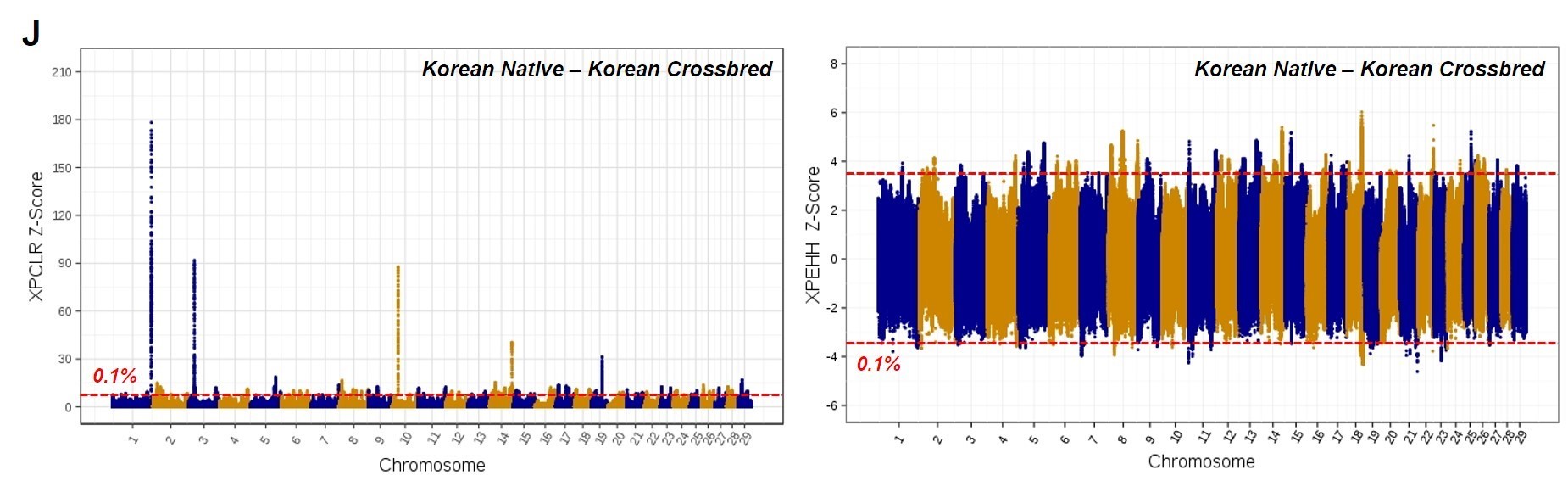
**

**Figure S12.** Haplotype and nucleotide diversity patterns for 14 goat populations in LBP and BPI genes associated with *Salmonella* infection pathway. **(A), (B)** Patterns for the entire region of the LBP gene located on 68,734,204 bp - 68,771,108 bp of chromosome 13. **(C), (D)** Patterns for the entire region of the BPI gene located on 68,691,139 bp - 68,722,074 bp of chromosome 13. The legend of the 14 goat populations is displayed on the right, and the red color represents Korean indigenous goat.

**
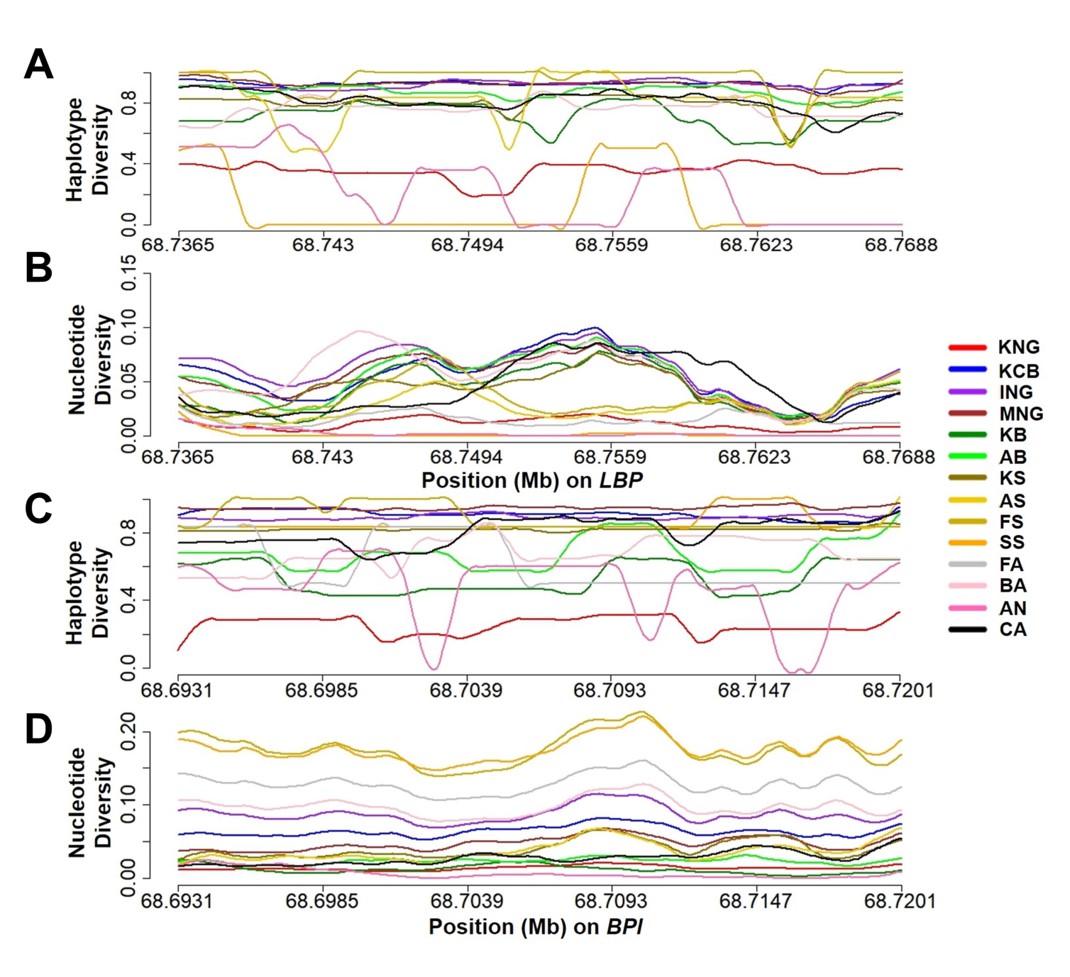
**

**Figure S13.** Patterns of the entire gene region of ITGB6 gene located on 38,768,167 bp - 38,910,516 bp region on chromosome 2, associated with the cardiomyopathy pathway. **(A)** Haplotype diversity, **(B)** nucleotide diversity, and **(C)** average linkage disequilibrium patterns for 14 goat populations. The legend of the 14 goat populations is displayed on the right, and the red color represents Korean indigenous goat.

**
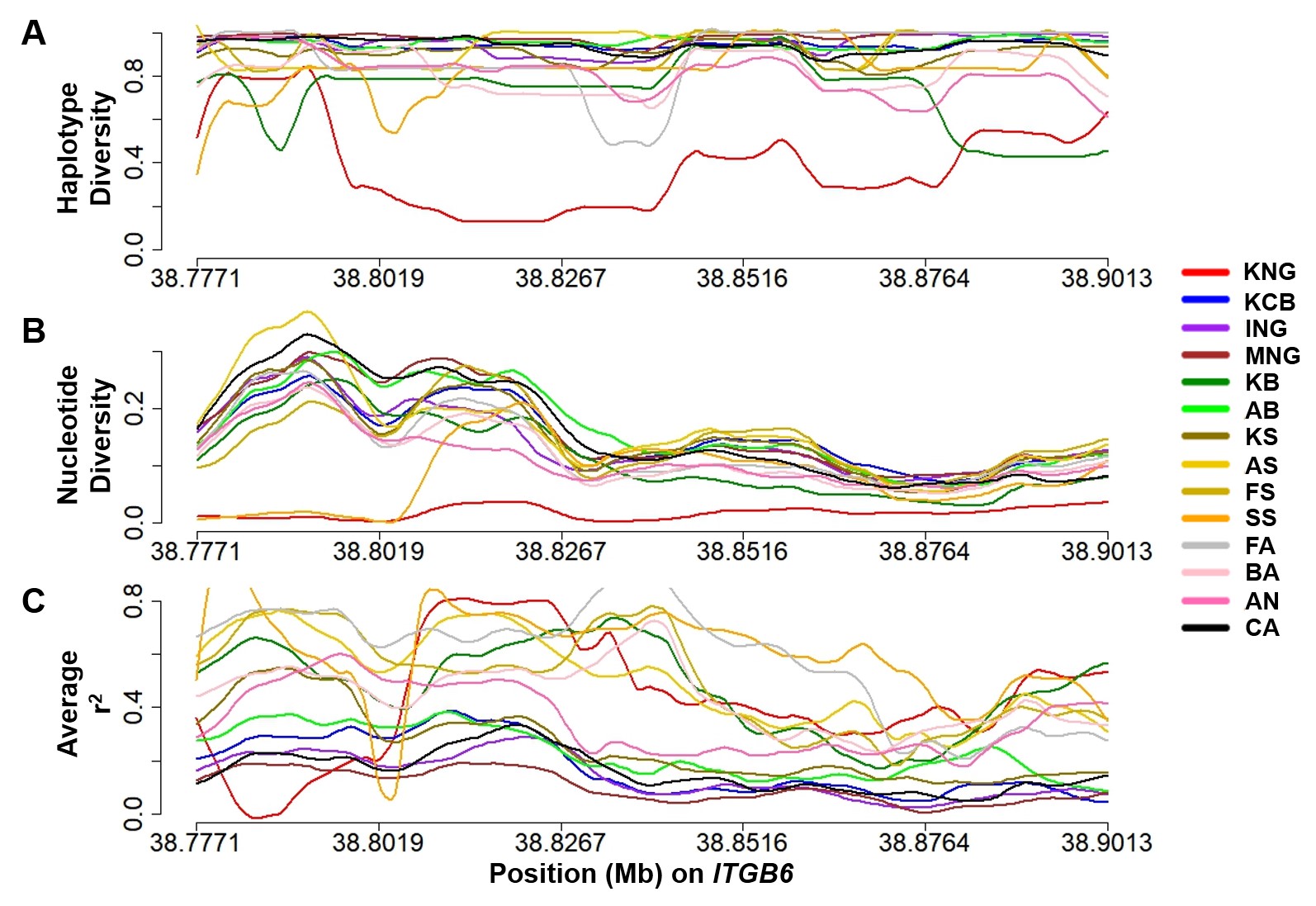
**

**Figure S14.** Selection signature for ITGB6 gene. Average LD, haplotype diversity, and nucleotide diversity patterns (above) and haplotype sharing pattern (bottom) of the ITGB6 gene in the region of **(A)** 38,768,167 bp - 38,847,042 bp, and **(B)** 38,847,329 bp-38,910,516 bp region on chromosome 2, respectively. In the haplotype sharing plot, the yellow rectangle highlights a pattern in which the Korean indigenous goat is differentiated from other goat populations.

**
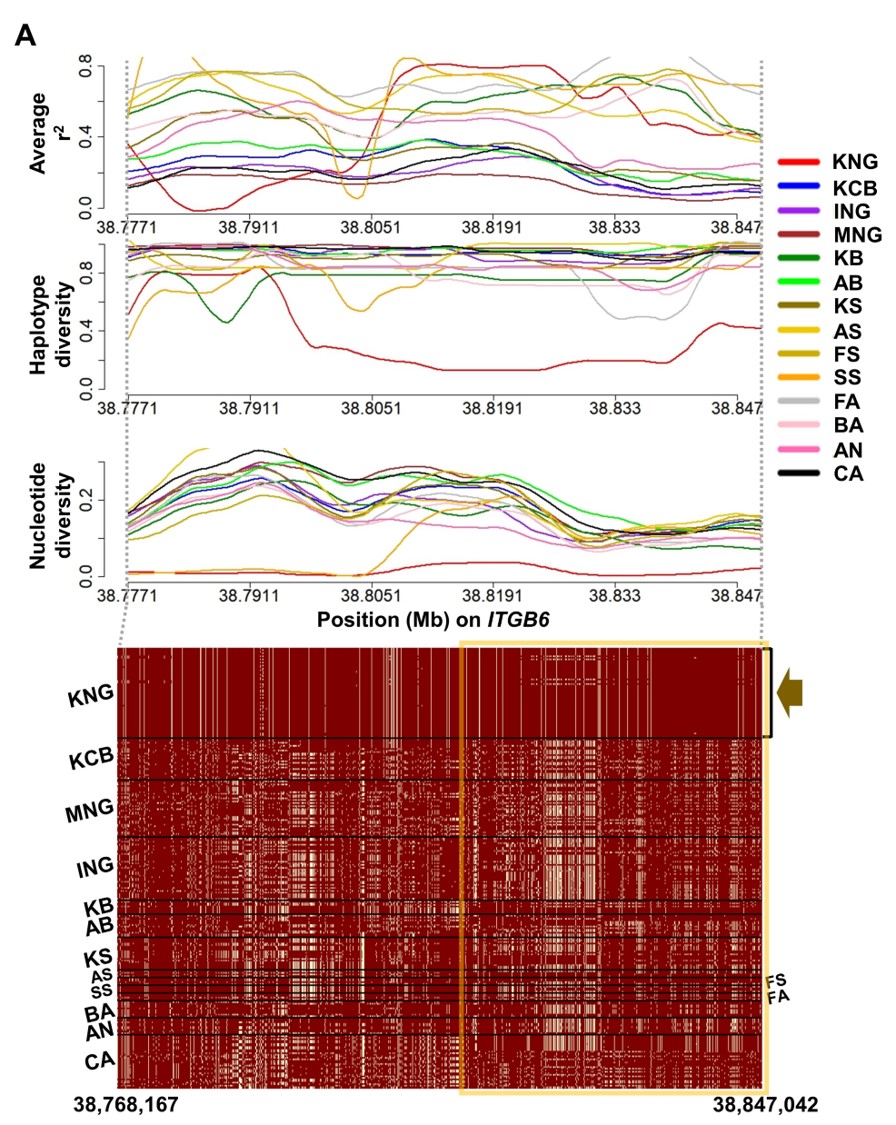
**

**Figure S14 (cont.).** Selection signature for ITGB6 gene. Average LD, haplotype diversity, and nucleotide diversity patterns (above) and haplotype sharing pattern (bottom) of the ITGB6 gene in the region of **(A)** 38,768,167 bp - 38,847,042 bp, and **(B)** 38,847,329 bp-38,910,516 bp region on chromosome 2, respectively. In the haplotype sharing plot, the yellow rectangle highlights a pattern in which the Korean indigenous goat is differentiated from other goat populations.

**
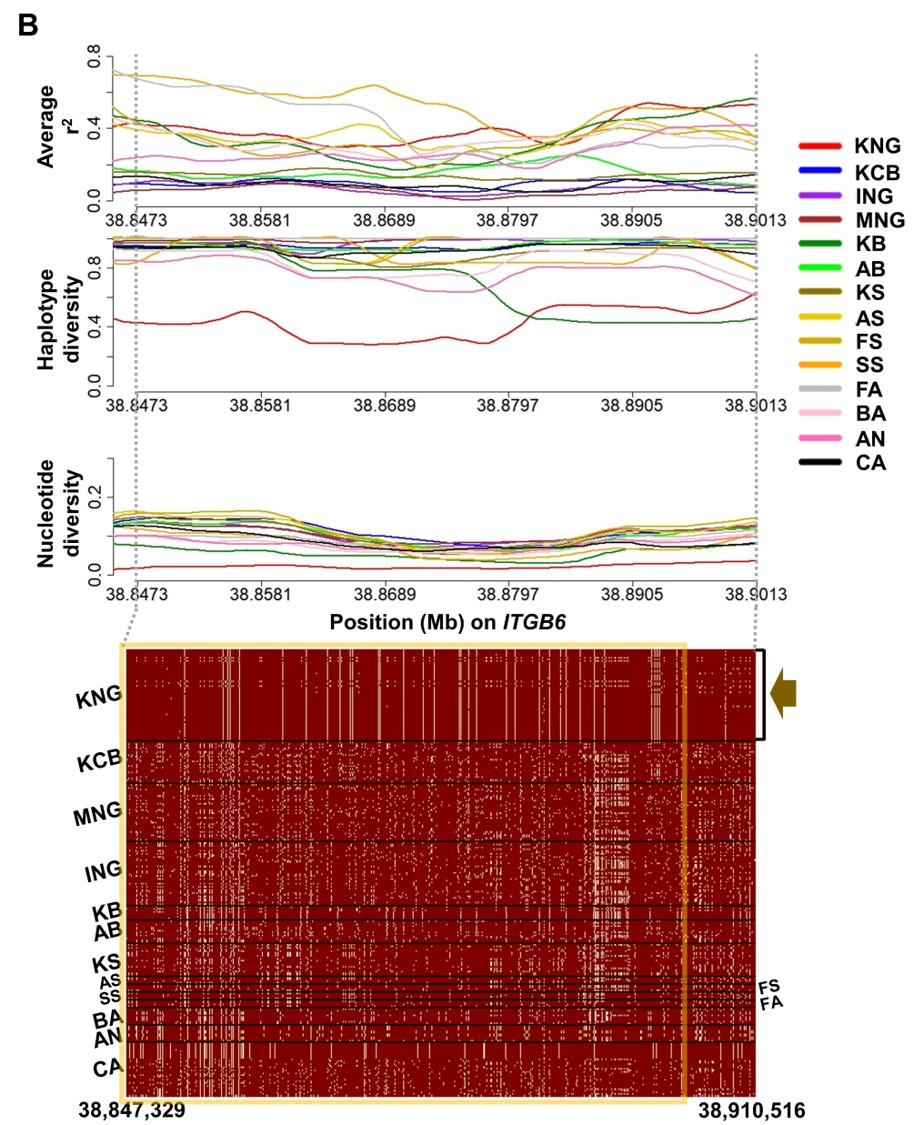
**

**Figure S15.** Patterns of the entire gene region of TTN gene located on 19,099,153 bp - 19,374,848 bp region on chromosome 2 and related to the cardiomyopathy pathway. **(A)** nucleotide diversity patterns for all 14 goat populations and **(B)** only Korean indigenous goat, respectively. **(C)** haplotype diversity patterns for all 14 goat populations and **(D)** only Korean indigenous goat, respectively**.** The legend of the 14 goat populations is displayed on the right, and the red color represents Korean indigenous goat.

**
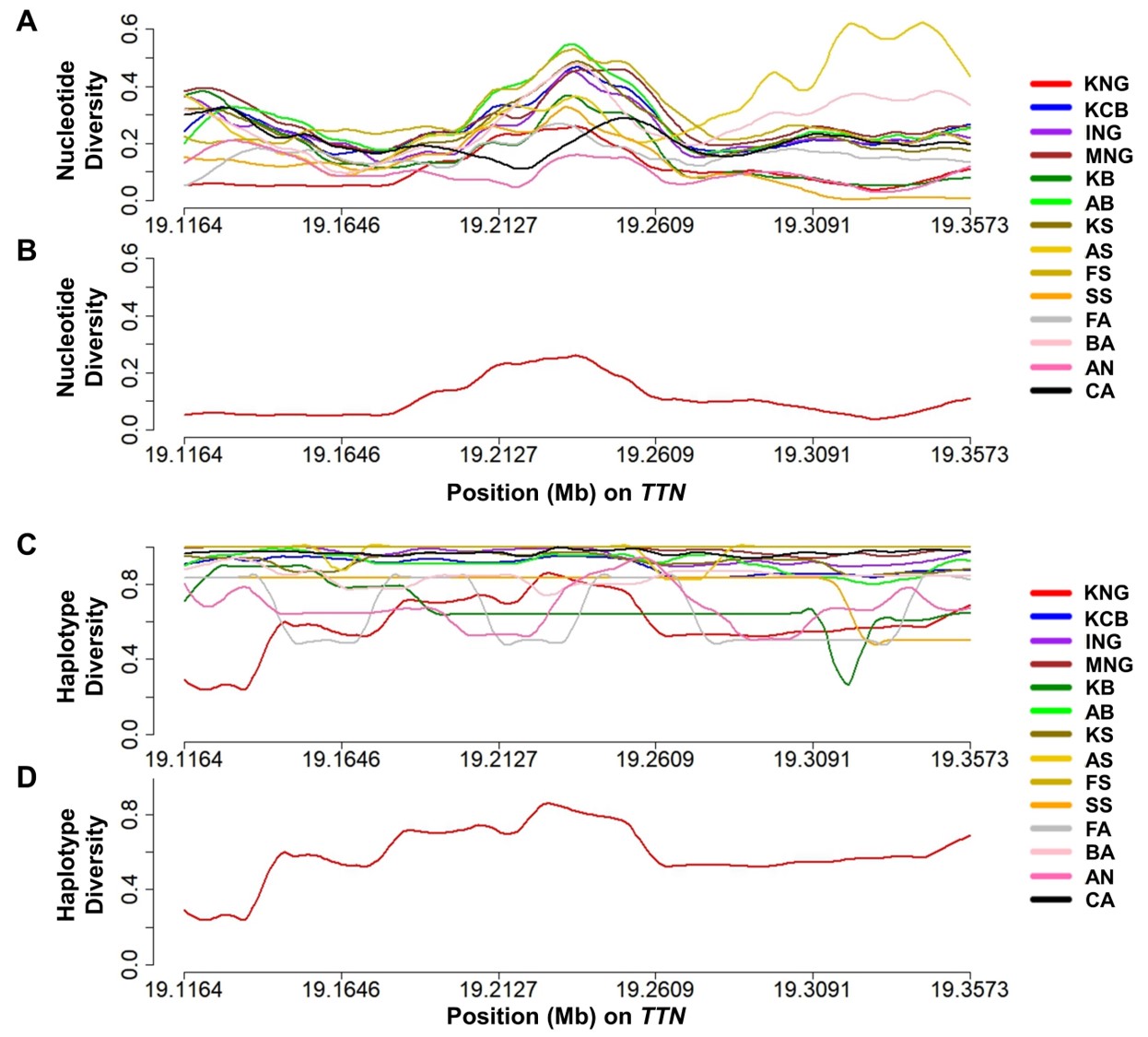
**

**Figure S16.** Selection signature for TTN gene. (**A)** Gene structure and haplotype frequencies of a region containing a missense SNP (p.Ile1202Thr) located on the 19,127,870 position on chromosome 2 in the TTN gene. **(B), (C), (D), (E), (F)** Haplotype and nucleotide diversity patterns (above) and haplotype sharing pattern (middle) of the TTN gene in the region of 19,099,153 bp - 19,374,848 bp region on chromosome 2. In the haplotype sharing plot, the yellow rectangle highlights a pattern in which the Korean indigenous goat is differentiated from other goat populations.

**
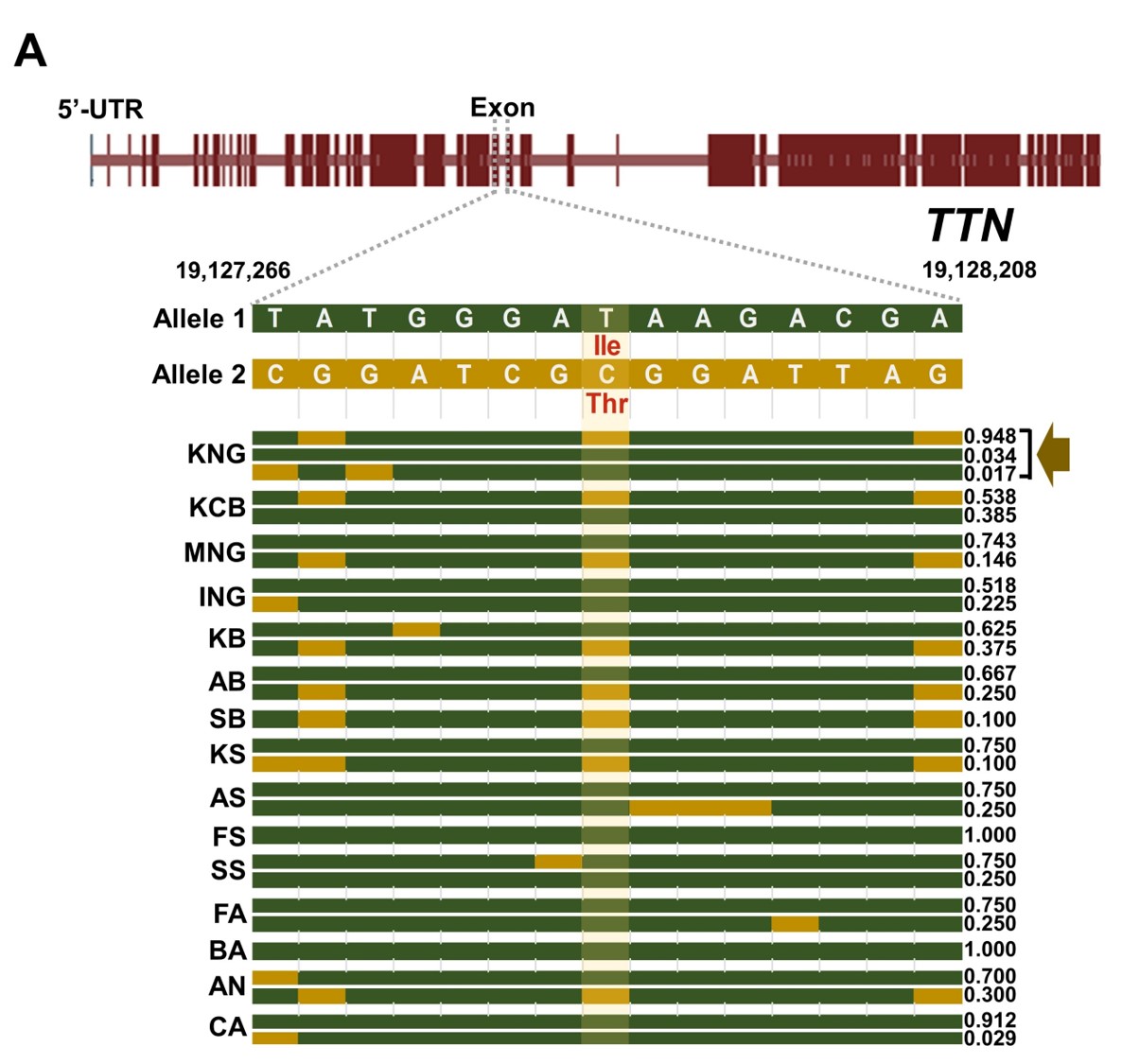
**

**Figure S16 (cont.).** Selection signature for TTN gene. (**A)** Gene structure and haplotype frequencies of a region containing a missense SNP (p.Ile1202Thr) located on the 19,127,870 position on chromosome 2 in the TTN gene. **(B), (C), (D), (E), (F)** Haplotype and nucleotide diversity patterns (above) and haplotype sharing pattern (middle) of the TTN gene in the region of 19,099,153 bp - 19,374,848 bp region on chromosome 2. In the haplotype sharing plot, the yellow rectangle highlights a pattern in which the Korean indigenous goat is differentiated from other goat populations.

**
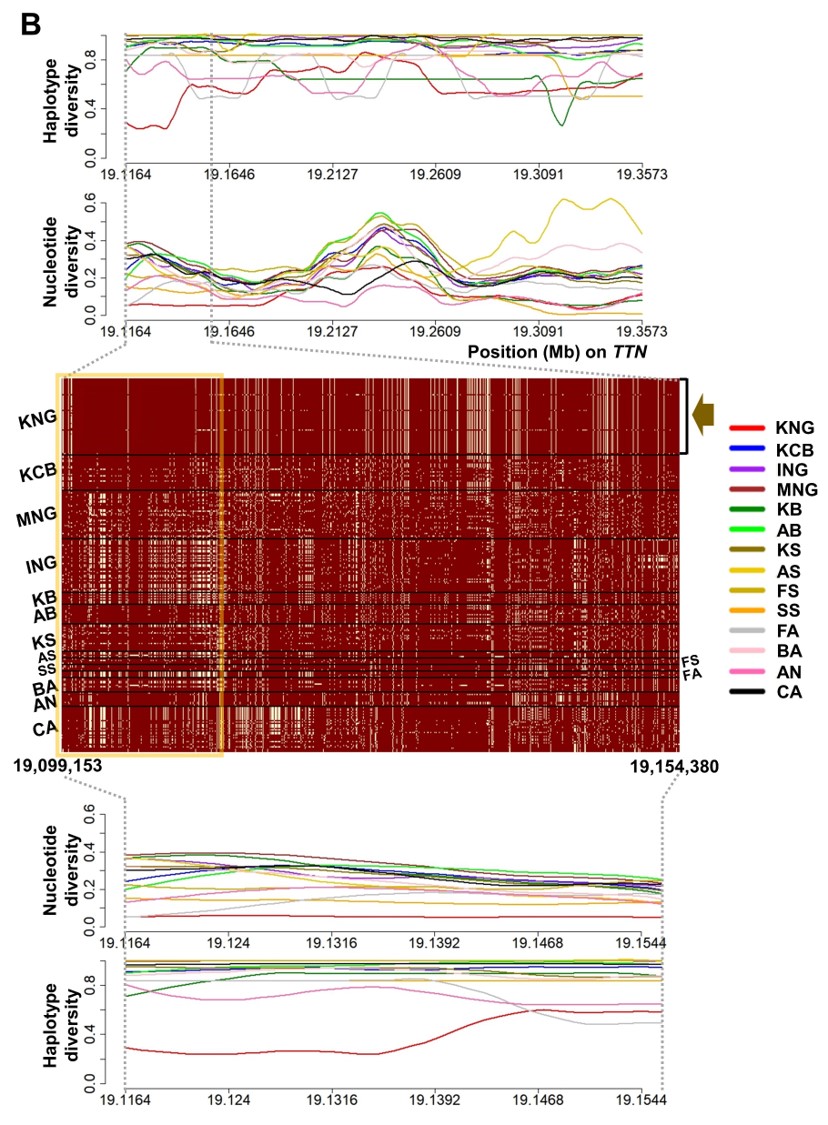
**

**Figure S16 (cont.).** Selection signature for TTN gene. (**A)** Gene structure and haplotype frequencies of a region containing a missense SNP (p.Ile1202Thr) located on the 19,127,870 position on chromosome 2 in the TTN gene. **(B), (C), (D), (E), (F)** Haplotype and nucleotide diversity patterns (above) and haplotype sharing pattern (middle) of the TTN gene in the region of 19,099,153 bp - 19,374,848 bp region on chromosome 2. In the haplotype sharing plot, the yellow rectangle highlights a pattern in which the Korean indigenous goat is differentiated from other goat populations.

**
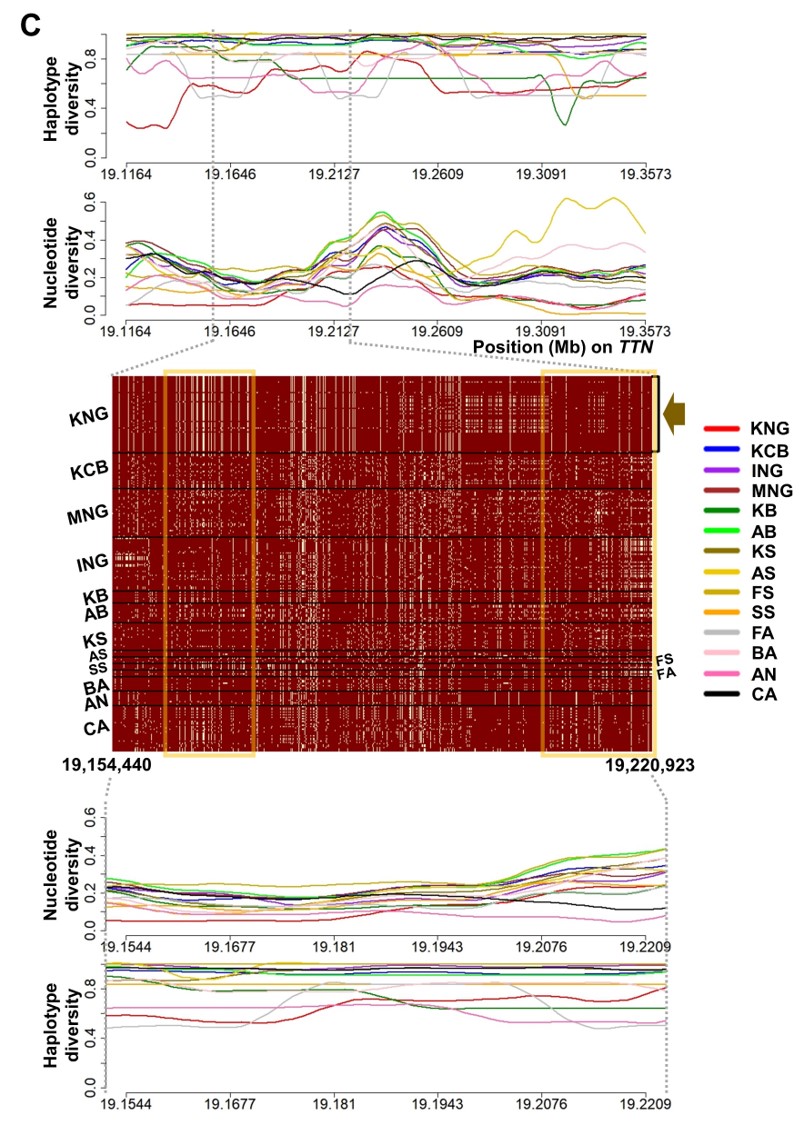
**

**Figure S16 (cont.).** Selection signature for TTN gene. (**A)** Gene structure and haplotype frequencies of a region containing a missense SNP (p.Ile1202Thr) located on the 19,127,870 position on chromosome 2 in the TTN gene. **(B), (C), (D), (E), (F)** Haplotype and nucleotide diversity patterns (above) and haplotype sharing pattern (middle) of the TTN gene in the region of 19,099,153 bp - 19,374,848 bp region on chromosome 2. In the haplotype sharing plot, the yellow rectangle highlights a pattern in which the Korean indigenous goat is differentiated from other goat populations.

**
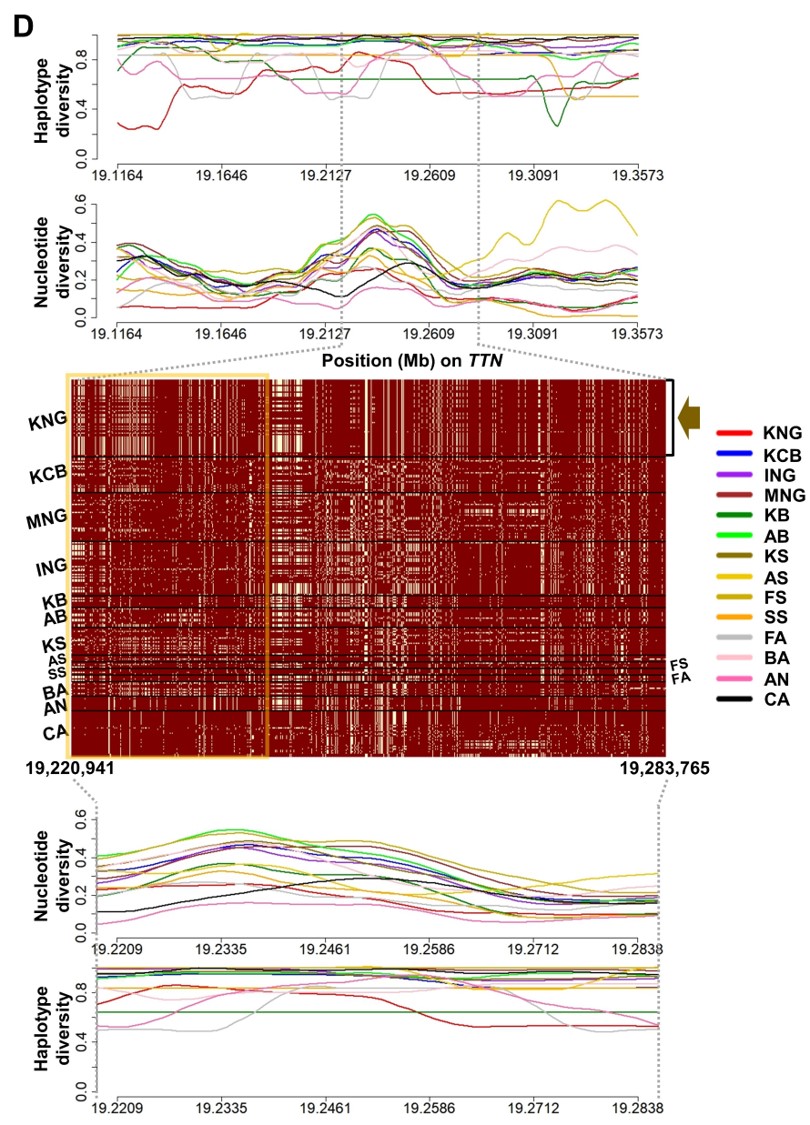
**

**Figure S16 (cont.).** Selection signature for TTN gene. (**A)** Gene structure and haplotype frequencies of a region containing a missense SNP (p.Ile1202Thr) located on the 19,127,870 position on chromosome 2 in the TTN gene. **(B), (C), (D), (E), (F)** Haplotype and nucleotide diversity patterns (above) and haplotype sharing pattern (middle) of the TTN gene in the region of 19,099,153 bp - 19,374,848 bp region on chromosome 2. In the haplotype sharing plot, the yellow rectangle highlights a pattern in which the Korean indigenous goat is differentiated from other goat populations.

**
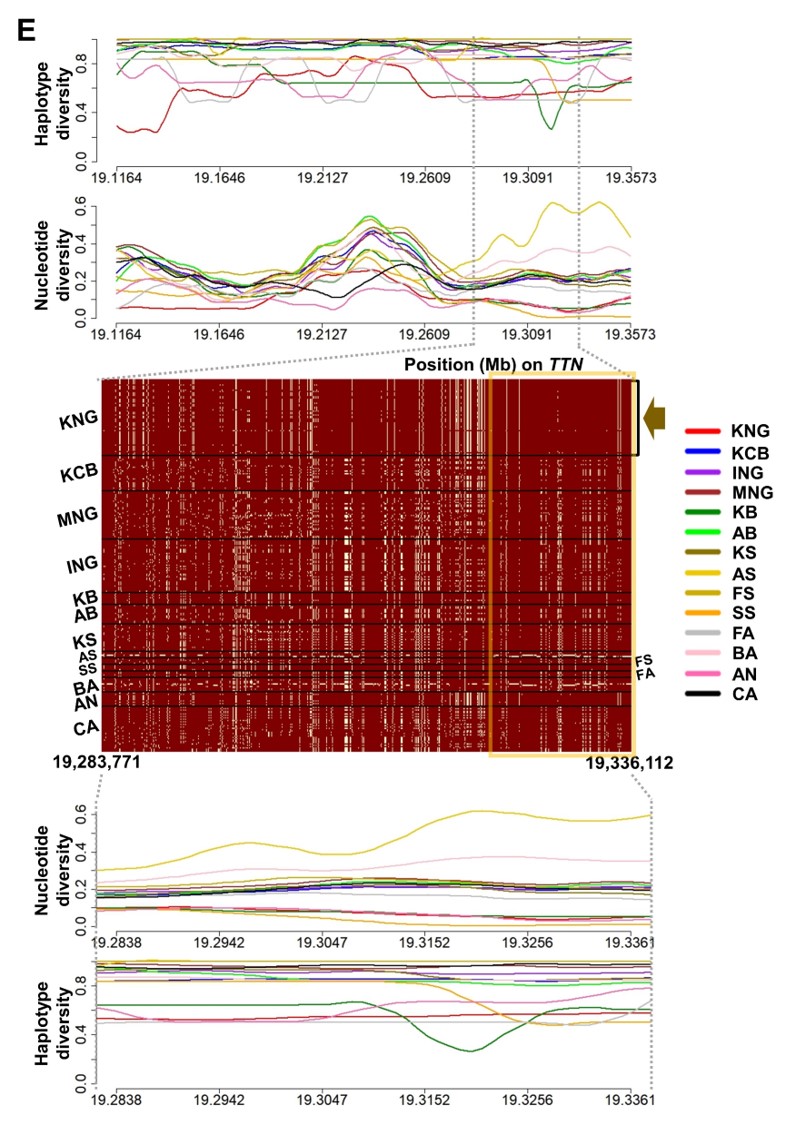
**

**Figure S16 (cont.).** Selection signature for TTN gene. (**A)** Gene structure and haplotype frequencies of a region containing a missense SNP (p.Ile1202Thr) located on the 19,127,870 position on chromosome 2 in the TTN gene. **(B), (C), (D), (E), (F)** Haplotype and nucleotide diversity patterns (above) and haplotype sharing pattern (middle) of the TTN gene in the region of 19,099,153 bp - 19,374,848 bp region on chromosome 2. In the haplotype sharing plot, the yellow rectangle highlights a pattern in which the Korean indigenous goat is differentiated from other goat populations.


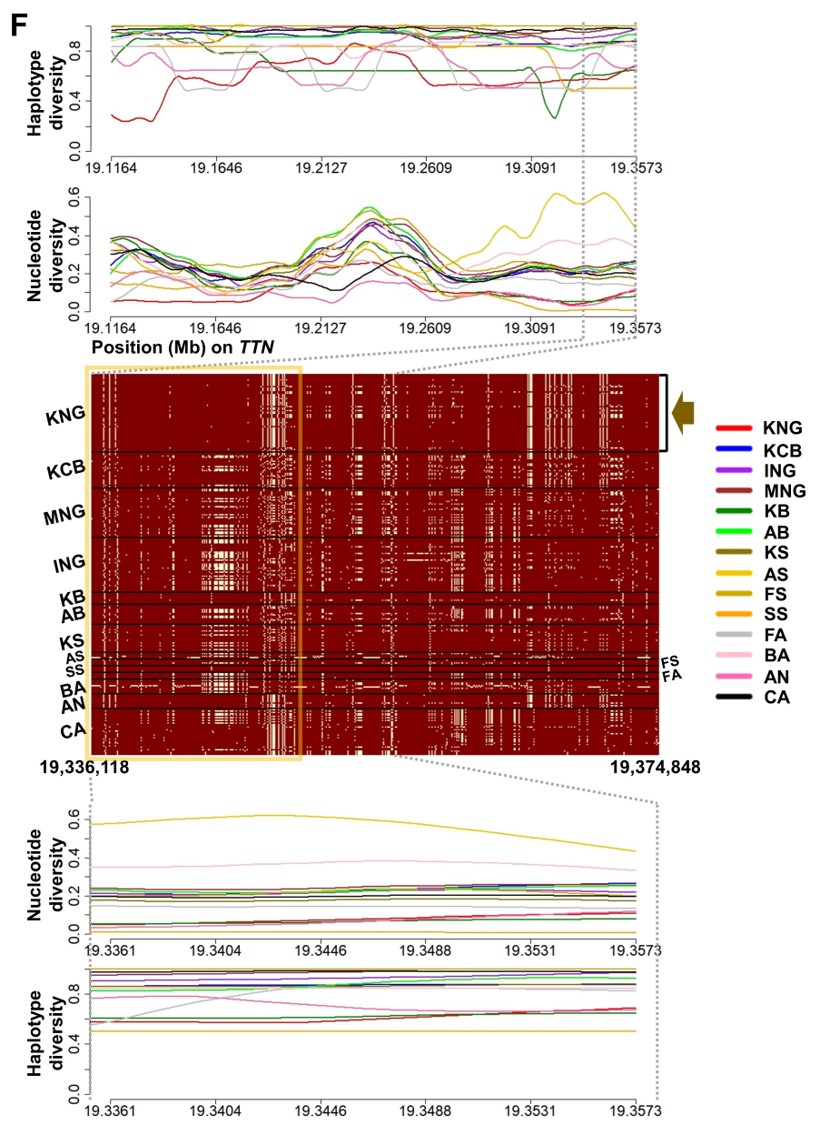


**S4: Supplementary Tables S1-S10**

**Table S1. The sample information of 90 publicly available goats.**

| **Breed** | **Sample Size** | **SRA study** | **Bio-project ID** |  |
| --- | --- | --- | --- | --- |
| Korean Indigenous Goat | 15 | SRP041525 | PRJNA245770 |  |
| Moroccan Indigenous Goat | 20 | ERP001578 | PRJEB3134 |  |
| Iranian Indigenous Goat | 18 | ERP001579 | PRJEB3135 |  |
| Korean crossbred | 13 | SRP041525 | PRJNA245770 |  |
| French Saanen | 2 | ERP005335 | PRJEB5900 |  |
| Swiss Saanen | 2 | SRP069284 | PRJNA310684 |  |
| French Alpine | 2 | ERP005335 | PRJEB5900 |  |
| Swiss Boer | 1 | SRP069284 | PRJNA310684 |  |
| *Capra aegagrus* | 5 | ERP001584 | PRJEB3140 |  |
| *Capra aegagrus* | 12 | ERP001580 | PRJEB3136 |  |
|  | | | |  |

**Table S2.** The data statistic for 90 publicly available goats.

| **Sample ID** | **SRA run ID** | **SRA study ID** | **SRA Sample ID** | **Bio-project ID** | **DNA Sequenced (bp)** | **Total**  **Reads** | **Alignment Rate (%)** | **Read Depth** | **Genome Coverage (%)** |
| --- | --- | --- | --- | --- | --- | --- | --- | --- | --- |
| Morocco Native1 | ERR219545 | ERP001578 | ERS154645 | PRJEB3134 | 39,282,768,222 | 404,519,671 | 99.81 | 14.42 | 98.9 |
| Morocco Native2 | ERR219546 | ERP001578 | ERS154656 | PRJEB3134 | 38,851,543,154 | 400,283,363 | 99.73 | 14.26 | 98.9 |
| Morocco Native3 | ERR219547 | ERP001578 | ERS154625 | PRJEB3134 | 38,068,834,332 | 390,782,339 | 99.8 | 13.97 | 98.9 |
| Morocco Native4 | ERR229468 | ERP001578 | ERS154550 | PRJEB3134 | 37,191,479,489 | 385,836,547 | 99.68 | 13.65 | 98.8 |
| Morocco Native5 | ERR229471 | ERP001578 | ERS154619 | PRJEB3134 | 38,204,531,960 | 398,264,901 | 99.53 | 14.02 | 98.9 |
| Morocco Native6 | ERR229476 | ERP001578 | ERS154602 | PRJEB3134 | 38,643,780,353 | 399,827,911 | 99.71 | 14.19 | 98.9 |
| Morocco Native7 | ERR229477 | ERP001578 | ERS154568 | PRJEB3134 | 35,950,245,171 | 377,401,319 | 99.61 | 13.2 | 98.8 |
| Morocco Native8 | ERR229478 | ERP001578 | ERS154608 | PRJEB3134 | 38,976,983,876 | 402,792,851 | 99.79 | 14.31 | 98.9 |
| Morocco Native9 | ERR229481 | ERP001578 | ERS154610 | PRJEB3134 | 38,882,826,164 | 401,713,390 | 99.7 | 14.27 | 98.9 |
| Morocco Native10 | ERR229484 | ERP001578 | ERS154598 | PRJEB3134 | 43,487,262,112 | 445,408,995 | 99.81 | 15.96 | 99 |
| Morocco Native11 | ERR229485 | ERP001578 | ERS154657 | PRJEB3134 | 37,056,935,130 | 386,719,872 | 99.68 | 13.6 | 98.9 |
| Morocco Native12 | ERR229486 | ERP001578 | ERS154618 | PRJEB3134 | 37,946,936,958 | 389,959,466 | 99.79 | 13.93 | 98.9 |
| Morocco Native13 | ERR229487 | ERP001578 | ERS154601 | PRJEB3134 | 37,935,730,282 | 390,793,264 | 99.71 | 13.93 | 98.9 |
| Morocco Native14 | ERR232487 | ERP001578 | ERS154583 | PRJEB3134 | 36,556,537,923 | 394,086,753 | 99.77 | 13.42 | 98.9 |
| Morocco Native15 | ERR232496 | ERP001578 | ERS154592 | PRJEB3134 | 35,715,303,754 | 382,576,806 | 99.77 | 13.11 | 98.8 |
| Morocco Native16 | ERR232497 | ERP001578 | ERS154631 | PRJEB3134 | 35,811,765,975 | 384,197,057 | 99.79 | 13.15 | 98.8 |
| Morocco Native17 | ERR234309 | ERP001578 | ERS154562 | PRJEB3134 | 41,685,862,368 | 425,285,743 | 99.8 | 15.3 | 99 |
| Morocco Native18 | ERR234310 | ERP001578 | ERS154547 | PRJEB3134 | 43,982,436,270 | 449,652,523 | 99.79 | 16.14 | 99 |
| Morocco Native19 | ERR234312 | ERP001578 | ERS154571 | PRJEB3134 | 40,589,553,098 | 413,879,093 | 99.78 | 14.9 | 98.9 |
| Morocco Native20 | ERR234316 | ERP001578 | ERS154681 | PRJEB3134 | 44,806,769,368 | 462,234,079 | 99.81 | 16.45 | 99 |
| Iran Native1 | ERR297229 | ERP001579 | ERS239041 | PRJEB3135 | 37,138,965,201 | 381,012,697 | 99.82 | 13.63 | 98.9 |
| Iran Native2 | ERR299449 | ERP001579 | ERS239034 | PRJEB3135 | 37,151,242,156 | 382,524,134 | 99.8 | 13.64 | 98.9 |
| Iran Native3 | ERR299456 | ERP001579 | ERS239027 | PRJEB3135 | 36,652,107,477 | 375,758,172 | 99.83 | 13.45 | 98.9 |

**Table S2 (cont.).** The data statistic for 90 publicly available goats.

| **Sample ID** | **SRA run ID** | **SRA study ID** | **SRA Sample ID** | **Bio-project ID** | **DNA Sequenced (bp)** | **Total**  **Reads** | **Alignment Rate (%)** | **Read Depth** | **Genome Coverage (%)** |
| --- | --- | --- | --- | --- | --- | --- | --- | --- | --- |
| Iran Native4 | ERR313198 | ERP001579 | ERS239031 | PRJEB3135 | 34,153,862,746 | 346,597,859 | 99.84 | 12.54 | 98.9 |
| Iran Native5 | ERR313199 | ERP001579 | ERS239035 | PRJEB3135 | 38,883,731,565 | 400,601,500 | 99.72 | 14.27 | 99 |
| Iran Native6 | ERR313200 | ERP001579 | ERS239029 | PRJEB3135 | 39,083,796,164 | 398,365,187 | 99.85 | 14.35 | 99 |
| Iran Native7 | ERR313202 | ERP001579 | ERS239036 | PRJEB3135 | 37,063,643,520 | 379,801,390 | 99.8 | 13.61 | 99 |
| Iran Native8 | ERR313204 | ERP001579 | ERS239040 | PRJEB3135 | 36,806,036,713 | 376,403,046 | 99.81 | 13.51 | 98.9 |
| Iran Native9 | ERR313207 | ERP001579 | ERS239033 | PRJEB3135 | 38,354,988,865 | 390,583,241 | 99.84 | 14.08 | 98.9 |
| Iran Native10 | ERR313209 | ERP001579 | ERS239037 | PRJEB3135 | 34,136,778,768 | 348,546,930 | 99.81 | 12.53 | 98.9 |
| Iran Native11 | ERR313210 | ERP001579 | ERS239039 | PRJEB3135 | 37,631,362,820 | 382,760,132 | 99.85 | 13.81 | 98.9 |
| Iran Native12 | ERR313211 | ERP001579 | ERS239030 | PRJEB3135 | 36,759,141,169 | 374,630,484 | 99.76 | 13.49 | 98.9 |
| Iran Native13 | ERR313212 | ERP001579 | ERS239026 | PRJEB3135 | 37,117,069,985 | 377,391,252 | 99.84 | 13.62 | 99 |
| Iran Native14 | ERR313213 | ERP001579 | ERS239028 | PRJEB3135 | 37,208,667,728 | 379,025,532 | 99.76 | 13.66 | 98.9 |
| Iran Native15 | ERR313215 | ERP001579 | ERS239042 | PRJEB3135 | 36,055,901,983 | 370,173,195 | 99.8 | 13.24 | 98.9 |
| Iran Native16 | ERR340332 | ERP001579 | ERS239044 | PRJEB3135 | 32,496,200,563 | 329,565,222 | 99.82 | 11.93 | 98.8 |
| Iran Native17 | ERR340337 | ERP001579 | ERS239043 | PRJEB3135 | 31,484,511,168 | 319,397,178 | 99.77 | 11.56 | 98.8 |
| Iran Native18 | ERR340339 | ERP001579 | ERS239032 | PRJEB3135 | 32,848,023,662 | 336,789,846 | 99.77 | 12.06 | 98.8 |
| Iran Capra Aegagrus1 | ERR219206 | ERP001584 | ERS154870 | PRJEB3140 | 20,882,655,447 | 296,339,525 | 99.36 | 7.67 | 98.2 |
| Iran Capra Aegagrus3 | ERR219210 | ERP001584 | ERS154870 | PRJEB3140 | 18,930,619,563 | 279,684,926 | 99.15 | 6.95 | 97.9 |
| Iran Capra Aegagrus6 | ERR219214 | ERP001584 | ERS154870 | PRJEB3140 | 25,798,973,150 | 264,409,400 | 98.84 | 9.47 | 98.2 |
| Iran Capra Aegagrus7 | ERR219216 | ERP001584 | ERS154870 | PRJEB3140 | 27,527,689,646 | 285,338,294 | 99.27 | 10.1 | 98.4 |
| Iran Capra Aegagrus8 | ERR219217 | ERP001584 | ERS154870 | PRJEB3140 | 26,437,846,527 | 275,660,830 | 98.94 | 9.7 | 98.3 |
| Iran Capra Aegagrus9 | ERR340330 | ERP001580 | ERS239025 | PRJEB3136 | 35,734,660,749 | 366,184,086 | 99.69 | 13.12 | 98.8 |
| Iran Capra Aegagrus10 | ERR340331 | ERP001580 | ERS239019 | PRJEB3136 | 33,790,126,472 | 345,791,278 | 99.73 | 12.4 | 98.7 |
| Iran Capra Aegagrus11 | ERR340334 | ERP001580 | ERS239007 | PRJEB3136 | 35,069,014,459 | 360,762,975 | 99.71 | 12.87 | 98.8 |

**Table S2 (cont.).** The data statistic for 90 publicly available goats.

| **Sample ID** | **SRA run ID** | **SRA study ID** | **SRA Sample ID** | **Bio-project ID** | **DNA Sequenced (bp)** | **Total**  **Reads** | **Alignment Rate (%)** | **Read Depth** | **Genome Coverage (%)** |
| --- | --- | --- | --- | --- | --- | --- | --- | --- | --- |
| Iran Capra Aegagrus12 | ERR340338 | ERP001580 | ERS239021 | PRJEB3136 | 32,922,765,329 | 334,566,973 | 99.77 | 12.09 | 98.7 |
| Iran Capra Aegagrus13 | ERR340340 | ERP001580 | ERS239015 | PRJEB3136 | 34,935,696,458 | 357,916,316 | 99.33 | 12.82 | 98.8 |
| Iran Capra Aegagrus14 | ERR340344 | ERP001580 | ERS239008 | PRJEB3136 | 28,165,657,718 | 331,004,267 | 99.52 | 10.34 | 98.6 |
| Iran Capra Aegagrus15 | ERR340345 | ERP001580 | ERS239010 | PRJEB3136 | 33,654,418,363 | 349,737,863 | 98.26 | 12.35 | 98.7 |
| Iran Capra Aegagrus16 | ERR340347 | ERP001580 | ERS239013 | PRJEB3136 | 36,695,280,328 | 376,425,769 | 99.74 | 13.47 | 98.8 |
| Iran Capra Aegagrus17 | ERR340426 | ERP001580 | ERS347739 | PRJEB3136 | 35,795,442,658 | 369,232,298 | 99.34 | 13.14 | 98.9 |
| Iran Capra Aegagrus18 | ERR470100 | ERP001580 | ERS419577 | PRJEB3136 | 43,118,803,724 | 442,200,262 | 99.41 | 15.83 | 98.7 |
| Iran Capra Aegagrus19 | ERR470104 | ERP001580 | ERS419576 | PRJEB3136 | 41,653,971,833 | 422,607,853 | 99.71 | 15.29 | 99 |
| Iran Capra Aegagrus20 | ERR470106 | ERP001580 | ERS419578 | PRJEB3136 | 42,079,307,151 | 451,420,898 | 95.29 | 15.45 | 98.7 |
| Korea Cross Breed1 | SRR1265081 | SRP041525 | SRS598518 | PRJNA245770 | 37,738,072,131 | 401,085,599 | 99.27 | 13.85 | 98.8 |
| Korea Cross Breed2 | SRR1265082 | SRP041525 | SRS598524 | PRJNA245770 | 34,243,517,038 | 364,475,021 | 99.13 | 12.57 | 98.7 |
| Korea Cross Breed3 | SRR1265088 | SRP041525 | SRS598525 | PRJNA245770 | 35,131,072,194 | 373,828,379 | 99.23 | 12.9 | 98.7 |
| Korea Cross Breed4 | SRR1265089 | SRP041525 | SRS598526 | PRJNA245770 | 37,751,747,602 | 401,448,208 | 99.3 | 13.86 | 98.9 |
| Korea Cross Breed5 | SRR1265097 | SRP041525 | SRS598537 | PRJNA245770 | 37,502,526,325 | 398,281,505 | 99.39 | 13.77 | 98.9 |
| Korea Cross Breed6 | SRR1265903 | SRP041525 | SRS598538 | PRJNA245770 | 35,416,161,963 | 378,336,441 | 98.93 | 13 | 98.8 |
| Korea Cross Breed7 | SRR1265904 | SRP041525 | SRS598539 | PRJNA245770 | 35,210,586,644 | 392,484,297 | 98.81 | 12.92 | 98.8 |
| Korea Cross Breed8 | SRR1265905 | SRP041525 | SRS598540 | PRJNA245770 | 36,761,891,538 | 391,526,744 | 99.14 | 13.49 | 98.8 |
| Korea Cross Breed9 | SRR1265906 | SRP041525 | SRS598541 | PRJNA245770 | 35,353,622,660 | 377,360,924 | 99 | 12.98 | 98.8 |
| Korea Cross Breed10 | SRR1265907 | SRP041525 | SRS598542 | PRJNA245770 | 33,037,016,831 | 366,323,855 | 99.15 | 12.13 | 98.7 |
| Korea Cross Breed11 | SRR1265908 | SRP041525 | SRS598543 | PRJNA245770 | 37,409,002,329 | 415,962,824 | 99.14 | 13.73 | 98.9 |
| Korea Cross Breed12 | SRR1265909 | SRP041525 | SRS598544 | PRJNA245770 | 38,280,762,649 | 425,210,161 | 99.11 | 14.05 | 98.9 |
| Korea Cross Breed13 | SRR1265910 | SRP041525 | SRS598545 | PRJNA245770 | 31,396,276,158 | 348,046,371 | 99.16 | 11.52 | 98.6 |
| Korea Native13 | SRR1265911 | SRP041525 | SRS598546 | PRJNA245770 | 34,237,071,853 | 425,036,369 | 99.2 | 12.57 | 98.6 |

**Table S2 (cont.).** The data statistic for 90 publicly available goats.

| **Sample ID** | **SRA run ID** | **SRA study ID** | **SRA Sample ID** | **Bio-project ID** | **DNA Sequenced (bp)** | **Total**  **Reads** | **Alignment Rate (%)** | **Read Depth** | **Genome Coverage (%)** |
| --- | --- | --- | --- | --- | --- | --- | --- | --- | --- |
| Korea Native14 | SRR1265923 | SRP041525 | SRS598547 | PRJNA245770 | 31,879,305,240 | 395,266,201 | 99.23 | 11.7 | 98.5 |
| Korea Native15 | SRR1265924 | SRP041525 | SRS598548 | PRJNA245770 | 31,798,665,386 | 395,678,426 | 99.01 | 11.67 | 98.5 |
| Korea Native16 | SRR1265926 | SRP041525 | SRS598549 | PRJNA245770 | 33,150,617,400 | 367,496,011 | 99.11 | 12.17 | 98.5 |
| Korea Native17 | SRR1265927 | SRP041525 | SRS598550 | PRJNA245770 | 32,696,270,069 | 361,469,629 | 99.32 | 12 | 98.6 |
| Korea Native18 | SRR1265928 | SRP041525 | SRS598551 | PRJNA245770 | 38,046,082,555 | 420,936,991 | 99.25 | 13.97 | 98.7 |
| Korea Native19 | SRR1265929 | SRP041525 | SRS598552 | PRJNA245770 | 37,372,316,158 | 412,941,923 | 99.37 | 13.72 | 98.6 |
| Korea Native20 | SRR1265930 | SRP041525 | SRS598553 | PRJNA245770 | 37,001,029,326 | 409,229,857 | 99.33 | 13.58 | 98.6 |
| Korea Native21 | SRR1265931 | SRP041525 | SRS598554 | PRJNA245770 | 37,031,700,313 | 408,942,849 | 99.4 | 13.59 | 98.6 |
| Korea Native22 | SRR1265932 | SRP041525 | SRS598555 | PRJNA245770 | 34,061,913,686 | 376,305,089 | 99.36 | 12.5 | 98.6 |
| Korea Native23 | SRR1265933 | SRP041525 | SRS598556 | PRJNA245770 | 30,464,056,844 | 336,650,991 | 99.26 | 11.18 | 98.6 |
| Korea Native24 | SRR1265934 | SRP041525 | SRS598557 | PRJNA245770 | 29,055,548,033 | 320,875,368 | 99.3 | 10.67 | 98.5 |
| Korea Native25 | SRR1265935 | SRP041525 | SRS598558 | PRJNA245770 | 28,590,163,843 | 316,092,685 | 99.25 | 10.49 | 98.5 |
| Korea Native26 | SRR1265936 | SRP041525 | SRS598559 | PRJNA245770 | 27,043,468,634 | 299,087,618 | 99.27 | 9.93 | 98.5 |
| Korea Native27 | SRR1265937 | SRP041525 | SRS598560 | PRJNA245770 | 29,493,894,796 | 325,574,317 | 99.37 | 10.83 | 98.5 |
| France Saanen1 | ERR470102 | ERP005335 | ERS423495 | PRJEB5900 | 38,275,719,582 | 386,563,936 | 99.85 | 14.05 | 98.9 |
| France Saanen2 | ERR470101 | ERP005335 | ERS423494 | PRJEB5900 | 38,941,498,406 | 398,326,728 | 99.73 | 14.29 | 98.9 |
| France Alpine1 | ERR470103 | ERP005335 | ERS423496 | PRJEB5900 | 41,175,552,440 | 416,443,500 | 99.83 | 15.11 | 99 |
| France Alpine2 | ERR470105 | ERP005335 | ERS423497 | PRJEB5900 | 40,214,654,830 | 406,125,183 | 99.85 | 14.76 | 99 |
| Swiss Sannen1 | SRR3144625 | SRP069284 | SRS1275352 | PRJNA310684 | 45,625,250,548 | 475,224,757 | 96.69 | 16.75 | 98.9 |
| Swiss Sannen2 | SRR3144630 | SRP069284 | SRS1275353 | PRJNA310684 | 30,444,566,993 | 329,632,298 | 96.79 | 11.18 | 98.6 |
| Swiss Boer1 | SRR3144618 | SRP069284 | SRS1275345 | PRJNA310684 | 31,888,720,755 | 322,047,873 | 99.24 | 11.71 | 98.6 |

**Table S3.** The data statistic for 46 newly re-sequenced goat samples (Accession number deposited in EBI: PRJEB25062).

| **Sample ID** | **ENA**  **Accession ID** | **ENA**  **Study ID** | **ENA**  **Sample ID** | **DNA**  **Sequenced (bp)** | **Total**  **Reads** | **Alignment**  **Rate (%)** | **Read**  **Depth** | **Genome Coverage (%)** |
| --- | --- | --- | --- | --- | --- | --- | --- | --- |
| Australia Anglo Nubian1 | ERR2309113 | ERP106943 | ERS2212966 | 33,600,245,813 | 347,763,494 | 99.49 | 12.33 | 98.9 |
| Australia Anglo Nubian2 | ERR2309114 | ERP106943 | ERS2212967 | 32,944,128,405 | 347,498,168 | 99.43 | 12.09 | 98.8 |
| Australia Anglo Nubian3 | ERR2309115 | ERP106943 | ERS2212968 | 33,991,719,499 | 354,018,559 | 99.41 | 12.48 | 98.8 |
| Australia Anglo Nubian4 | ERR2309116 | ERP106943 | ERS2212969 | 31,895,162,470 | 330,840,515 | 99.45 | 11.71 | 98.8 |
| Australia Anglo Nubian5 | ERR2309117 | ERP106943 | ERS2212970 | 30,007,265,990 | 319,625,644 | 99.41 | 11.01 | 98.6 |
| Australia Boer1 | ERR2309118 | ERP106943 | ERS2212971 | 32,308,137,201 | 353,620,690 | 99.41 | 11.86 | 98.5 |
| Australia Boer2 | ERR2309119 | ERP106943 | ERS2212972 | 34,695,027,142 | 378,918,870 | 99.4 | 12.74 | 98.6 |
| Australia Boer3 | ERR2309120 | ERP106943 | ERS2212973 | 35,833,230,465 | 387,500,604 | 99.44 | 13.15 | 98.8 |
| Australia Boer4 | ERR2309121 | ERP106943 | ERS2212974 | 35,654,669,029 | 374,819,849 | 99.36 | 13.09 | 99 |
| Australia Boer5 | ERR2309122 | ERP106943 | ERS2212975 | 31,549,340,425 | 333,231,265 | 99.39 | 11.58 | 98.7 |
| Australia Boer6 | ERR2309123 | ERP106943 | ERS2212976 | 32,889,040,758 | 352,057,315 | 99.41 | 12.07 | 98.8 |
| Australia British Alpine1 | ERR2309108 | ERP106943 | ERS2212961 | 29,656,512,856 | 300,681,077 | 99.55 | 10.89 | 98.7 |
| Australia British Alpine2 | ERR2309109 | ERP106943 | ERS2212962 | 31,445,982,484 | 317,471,498 | 99.67 | 11.54 | 98.7 |
| Australia British Alpine3 | ERR2309110 | ERP106943 | ERS2212963 | 30,257,536,780 | 319,658,733 | 99.27 | 11.11 | 98.6 |
| Australia British Alpine4 | ERR2309111 | ERP106943 | ERS2212964 | 31,524,370,472 | 328,222,440 | 99.49 | 11.57 | 98.8 |
| Australia British Alpine5 | ERR2309112 | ERP106943 | ERS2212965 | 34,647,946,982 | 359,833,350 | 99.45 | 12.72 | 98.9 |
| Australia Saanen1 | ERR2309106 | ERP106943 | ERS2212959 | 31,146,811,443 | 325,028,012 | 99.47 | 11.43 | 98.8 |
| Australia Saanen2 | ERR2309107 | ERP106943 | ERS2212960 | 29,427,545,059 | 308,304,404 | 99.11 | 10.8 | 98.7 |
| Korea Boer1 | ERR2309134 | ERP106943 | ERS2212987 | 33,909,290,592 | 341,798,208 | 99.89 | 12.45 | 98.2 |
| Korea Boer2 | ERR2309135 | ERP106943 | ERS2212988 | 34,656,397,330 | 349,238,476 | 99.9 | 12.72 | 98.2 |
| Korea Boer3 | ERR2309136 | ERP106943 | ERS2212989 | 31,265,543,639 | 314,771,750 | 99.86 | 11.48 | 97.9 |
| Korea Boer4 | ERR2309137 | ERP106943 | ERS2212990 | 33,555,113,271 | 338,510,144 | 99.84 | 12.32 | 98.7 |
| Korea Native1 | ERR2309138 | ERP106943 | ERS2212991 | 33,215,076,432 | 371,582,074 | 93.24 | 12.19 | 98.3 |
| Korea Native2 | ERR2309139 | ERP106943 | ERS2212992 | 31,587,585,000 | 353,478,759 | 99.8 | 11.6 | 98.3 |

**Table S3 (cont.).** The data statistic for 46 newly re-sequenced goat samples (Accession number deposited in EBI: PRJEB25062).

| **Sample ID** | **ENA**  **Accession ID** | **ENA**  **Study ID** | **ENA**  **Sample ID** | **DNA Sequenced (bp)** | **Total**  **Reads** | **Alignment**  **Rate (%)** | **Read**  **Depth** | **Genome Coverage (%)** |
| --- | --- | --- | --- | --- | --- | --- | --- | --- |
| Korea Native3 | ERR2309140 | ERP106943 | ERS2212993 | 30,777,651,543 | 355,550,726 | 99.92 | 11.3 | 98.3 |
| Korea Native4 | ERR2309141 | ERP106943 | ERS2212994 | 28,320,031,092 | 325,566,556 | 99.92 | 10.4 | 98.1 |
| Korea Native5 | ERR2309142 | ERP106943 | ERS2212995 | 34,088,136,267 | 380,947,641 | 99.91 | 12.51 | 98.3 |
| Korea Native6 | ERR2309143 | ERP106943 | ERS2212996 | 28,138,169,346 | 314,488,763 | 99.93 | 10.33 | 98.1 |
| Korea Native7 | ERR2309144 | ERP106943 | ERS2212997 | 30,754,316,475 | 343,804,684 | 99.92 | 11.29 | 98.3 |
| Korea Native8 | ERR2309145 | ERP106943 | ERS2212998 | 30,525,664,094 | 341,789,010 | 99.81 | 11.21 | 98.2 |
| Korea Native9 | ERR2309146 | ERP106943 | ERS2212999 | 30,934,521,754 | 345,881,886 | 99.92 | 11.36 | 98.2 |
| Korea Native10 | ERR2309147 | ERP106943 | ERS2213000 | 30,635,170,160 | 342,834,824 | 99.81 | 11.25 | 98.3 |
| Korea Native11 | ERR2309148 | ERP106943 | ERS2213001 | 32,081,059,079 | 359,241,713 | 99.79 | 11.78 | 98.3 |
| Korea Native12 | ERR2309149 | ERP106943 | ERS2213002 | 34,593,864,488 | 386,687,004 | 99.91 | 12.7 | 98.5 |
| Korea Native28^a^ | ERR2309150, ERR2309151 | ERP106943, ERP106943 | ERS2213003, ERS2213004 | 37,853,822,165 | 434,183,493 | 99.8 | 13.9 | 98.5 |
| Korea Native29^a^ | [ERR2309152](https://www.ncbi.nlm.nih.gov/Traces/sra/?run=ERR2309152), [ERR230915](https://www.ncbi.nlm.nih.gov/Traces/sra/?run=ERR2309152)3 | ERP106943, ERP106943 | ERS2213005, ERS2213006 | 37,119,446,978 | 415,553,478 | 99.78 | 13.63 | 98.5 |
| Korea Saanen1 | ERR2309124 | ERP106943 | ERS2212977 | 39,089,849,048 | 393,443,027 | 99.94 | 14.35 | 98.5 |
| Korea Saanen2 | ERR2309125 | ERP106943 | ERS2212978 | 37,446,692,654 | 377,446,603 | 99.94 | 13.75 | 98.5 |
| Korea Saanen3 | ERR2309126 | ERP106943 | ERS2212979 | 35,101,943,511 | 353,219,128 | 99.94 | 12.89 | 98.3 |
| Korea Saanen4 | ERR2309127 | ERP106943 | ERS2212980 | 38,210,517,627 | 385,093,710 | 99.83 | 14.03 | 98.4 |
| Korea Saanen5 | ERR2309128 | ERP106943 | ERS2212981 | 32,926,283,770 | 332,604,867 | 99.84 | 12.09 | 98.3 |
| Korea Saanen6 | ERR2309129 | ERP106943 | ERS2212982 | 32,179,173,731 | 323,492,921 | 99.96 | 11.81 | 97.9 |
| Korea Saanen7 | ERR2309130 | ERP106943 | ERS2212983 | 35,338,902,724 | 355,946,478 | 99.96 | 12.97 | 98.1 |
| Korea Saanen8 | ERR2309131 | ERP106943 | ERS2212984 | 35,065,653,104 | 353,713,296 | 99.85 | 12.87 | 98.1 |
| Korea Saanen9 | ERR2309132 | ERP106943 | ERS2212985 | 33,743,144,130 | 340,341,735 | 99.85 | 12.39 | 98.1 |
| Korea Saanen10 | ERR2309133 | ERP106943 | ERS2212986 | 35,036,287,987 | 369,977,113 | 99.8 | 12.86 | 98.3 |

^a^The “Korea Native28” and “Korea Native29” samples were sequenced twice. The two paired-end files of each sample were separately deposited in the ENA public database, and this analysis was performed by pooling these two files.

**Table S4. The number of variants of 136 goat samples detected for each chromosome.**

| **Chromosome** | **Length (bp)^a^** | **Numbers of total variants** | **Number of**  **filtered variants^b^** | **Numbers of  INDEL variants** | **Numbers of  bi-allelic SNPs** | **Average distance  between SNPs** |
| --- | --- | --- | --- | --- | --- | --- |
| 1 | 161,917,960 | 3,136,864 | 3,033,854 | 324,730 | 2,667,220 | 60.71 |
| 2 | 142,713,314 | 2,580,832 | 2,477,822 | 263,053 | 2,200,044 | 64.87 |
| 3 | 123,911,021 | 2,169,382 | 2,066,372 | 218,123 | 1,849,436 | 67.00 |
| 4 | 123,319,836 | 2,280,795 | 2,177,785 | 234,818 | 1,941,110 | 63.53 |
| 5 | 123,141,556 | 2,181,615 | 2,078,605 | 222,368 | 1,852,896 | 66.46 |
| 6 | 120,061,775 | 2,371,051 | 2,268,041 | 244,643 | 2,010,645 | 59.71 |
| 7 | 111,947,015 | 1,934,452 | 1,831,442 | 198,355 | 1,643,151 | 68.13 |
| 8 | 115,850,422 | 2,104,182 | 2,001,172 | 213,763 | 1,794,649 | 64.55 |
| 9 | 95,715,379 | 1,741,402 | 1,638,392 | 180,563 | 1,483,707 | 64.51 |
| 10 | 103,731,018 | 1,795,440 | 1,692,430 | 184,047 | 1,532,953 | 67.67 |
| 11 | 111,528,667 | 1,894,415 | 1,791,405 | 189,257 | 1,614,280 | 69.09 |
| 12 | 87,684,794 | 1,736,448 | 1,633,438 | 179,319 | 1,444,210 | 60.71 |
| 13 | 85,971,111 | 1,471,417 | 1,368,407 | 146,402 | 1,263,048 | 68.07 |
| 14 | 95,176,881 | 1,751,008 | 1,647,998 | 177,901 | 1,491,211 | 63.83 |
| 15 | 86,022,100 | 1,661,607 | 1,558,597 | 167,800 | 1,404,461 | 61.25 |
| 16 | 83,288,587 | 1,499,865 | 1,396,855 | 151,725 | 1,273,963 | 65.38 |
| 17 | 75,397,539 | 1,373,894 | 1,270,884 | 141,522 | 1,162,463 | 64.86 |
| 18 | 67,588,387 | 1,063,948 | 960,938 | 105,264 | 895,565 | 75.47 |
| 19 | 66,178,820 | 1,105,058 | 1,002,048 | 110,717 | 937,718 | 70.57 |
| 20 | 74,161,552 | 1,441,414 | 1,338,404 | 145,518 | 1,229,547 | 60.32 |
| 21 | 71,319,391 | 1,316,706 | 1,213,696 | 132,745 | 1,115,329 | 63.94 |
| 22 | 63,495,901 | 1,080,289 | 977,279 | 107,877 | 924,897 | 68.65 |
|  | | | | | | |
|  | | | | | | |

**Table S4 (cont.). The number of variants of 136 goat samples detected for each chromosome.**

| **Chromosome** | **Length (bp)^a^** | **Numbers of total variants** | **Number of**  **filtered variants^b^** | **Numbers of  INDEL variants** | **Numbers of  bi-allelic SNPs** | **Average distance  between SNPs** |
| --- | --- | --- | --- | --- | --- | --- |
| 23 | 53,682,125 | 1,026,887 | 923,877 | 861,557 | 62.31 | 62.31 |
| 24 | 65,280,213 | 1,232,591 | 1,129,581 | 124,849 | 1,049,177 | 62.22 |
| 25 | 45,931,244 | 741,336 | 638,326 | 71,357 | 630,411 | 72.86 |
| 26 | 52,998,766 | 1,010,194 | 907,184 | 101,133 | 859,896 | 61.63 |
| 27 | 47,299,517 | 914,255 | 811,245 | 94,705 | 780,144 | 60.63 |
| 28 | 45,893,513 | 912,409 | 809,399 | 91,973 | 779,224 | 58.90 |
| 29 | 52,633,165 | 977,566 | 874,556 | 95,728 | 827,545 | 63.60 |
| X | 131,643,222 | 1,493,279 | 1,390,269 | 147,709 | 1,171,392 | 112.38 |
| ^a^Based on CHIR2.0 genome of reference. | | | | | | |
| ^b^The number of variants that have filtered out variants with genotype missing rate > 50%. | | | | | | |

**Table S5.** The number of variants detected in 17 goat populations including the entire Saanen group and the entire Boer group.

| **Group** | **Sample size** | **Numbers of total variants** | **Number of**  **filtered variants^a^** | **Numbers of  INDEL variants** | **Numbers of  bi-allele SNPs** |
| --- | --- | --- | --- | --- | --- |
| ***Capra aegagrus*** | 17 | 47,339,700 | 43,095,834 | 4,622,198 | 39,222,625 |
| **Korean Indigenous Goat** | 29 | 45,946,288 | 41,528,354 | 4,544,873 | 37,715,208 |
| **Iranian Indigenous Goat** | 18 | 41,803,928 | 39,615,249 | 4,681,993 | 35,742,191 |
| **Moroccan Indigenous Goat** | 20 | 38,607,480 | 36,535,300 | 4,405,593 | 32,914,220 |
| **Korean crossbred** | 13 | 40,171,798 | 37,061,579 | 4,339,870 | 33,464,841 |
| **British-Alpine** | 5 | 41,624,527 | 36,643,921 | 3,305,863 | 27,509,379 |
| **Anglo-Nubian** | 5 | 43,073,289 | 26,954,569 | 3,007,123 | 23,726,534 |
| **Entire Saanen group** | 16 | 44,350,369 | 40,543,879 | 4,429,033 | 36,845,217 |
| *Korean Saanen* | *10* | 34,684,402 | 30,519,270 | 3,573,192 | 27,580,008 |
| *Australian Saanen* | *2* | 36,462,269 | 24,519,886 | 2,861,809 | 21,438,252 |
| *Swiss Saanen* | *2* | 20,909,519 | 18,534,496 | 2,534,680 | 16,498,364 |
| *French Saanen* | *2* | 17,908,852 | 16,068,339 | 2,396,453 | 14,147,817 |
| **Entire Boer group** | 11 | 47,121,982 | 35,468,084 | 3,658,465 | 32,384,827 |
| *Korean Boer* | *4* | 26,471,553 | 21,619,878 | 2,681,446 | 19,434,940 |
| *Australian Boer* | *6* | 46,549,979 | 25,461,854 | 2,796,444 | 23,126,047 |
| *Swiss Boer* | *1* | 17,277,461 | 6,945,064 | 967,387 | 6,169,011 |
| **French Alpine** | 2 | 16,671,886 | 15,094,736 | 2,340,430 | 13,219,860 |

^a^Variants filtered out having the missing rate of the genotype of more than 50%.

**Table S6.** The summary of variants annotated by SnpEFF for 17 goat populations including the entire Saanen group and the Boer group.

|  | ***Capra aegagrus*** | | **Korean Indigenous** | | **Iranian Indigenous** | | **Moroccan Indigenous** | | **Korean crossbred** | |
| --- | --- | --- | --- | --- | --- | --- | --- | --- | --- | --- |
|  | **SNP** | **INDEL** | **SNP** | **INDEL** | **SNP** | **INDEL** | **SNP** | **INDEL** | **SNP** | **INDEL** |
| **Region** |  |  |  |  |  |  |  |  |  |  |
| Down stream | 2,297,971 | 392,146 | 2,164,808 | 372,114 | 2,069,227 | 407,528 | 1,967,024 | 386,269 | 1,942,621 | 372,049 |
| Exon | 444,958 | 19,212 | 412,302 | 16,265 | 406,114 | 18,966 | 399,370 | 18,564 | 386,306 | 17,041 |
| Gene | - | 119 | - | 112 | - | 113 | - | 79 | - | 78 |
| Intergenic | 27,299,228 | 4,132,475 | 26,338,911 | 4,141,925 | 25,077,414 | 4,560,733 | 22,939,549 | 4,341,553 | 23,357,017 | 4,172,789 |
| Intron | 22,008,256 | 3,390,423 | 21,076,127 | 3,339,788 | 19,461,807 | 3,585,477 | 18,041,763 | 3,390,121 | 18,609,452 | 3,320,584 |
| Splice site acceptor | 645 | 295 | 567 | 284 | 536 | 274 | 558 | 245 | 528 | 248 |
| Splice site donor | 903 | 456 | 802 | 451 | 815 | 428 | 802 | 367 | 770 | 403 |
| Splice site region | 43,080 | 6,647 | 40,279 | 6,599 | 38,809 | 7,329 | 37,267 | 7,010 | 37,470 | 6,341 |
| Transcript | - | 266 | - | 234 | - | 314 | - | 313 | - | 276 |
| Upstream | 2,275,300 | 373,371 | 2,100,382 | 344,824 | 2,055,110 | 391,719 | 1,916,438 | 370,701 | 1,892,347 | 355,844 |
| UTR 3' | 336,497 | 61,500 | 320,635 | 60,757 | 293,033 | 66,270 | 277,947 | 62,411 | 290,718 | 59,602 |
| UTR 5' | 78,425 | 10,015 | 68,128 | 8,135 | 72,963 | 10,266 | 70,398 | 9,810 | 67,460 | 9,059 |
| **Functional class** |  |  |  |  |  |  |  |  |  |  |
| Missenese | 203,849 | 4,034 | 188,265 | 3,478 | 184,017 | 4,063 | 180,021 | 4,066 | 175,289 | 3,746 |
| Nonsense | 3,165 | 89 | 2,843 | 80 | 2,873 | 96 | 2,876 | 95 | 2,649 | 90 |
| Silent | 241,450 | 3,793 | 224,869 | 3,425 | 222,339 | 3,875 | 219,339 | 3,782 | 211,506 | 3,684 |
| **Impact** |  |  |  |  |  |  |  |  |  |  |
| High | 5,502 | 7,290 | 4,918 | 6,263 | 4,951 | 7,089 | 4,965 | 6,806 | 4,649 | 6,131 |
| Low | 291,755 | 10,632 | 271,186 | 10,183 | 268,029 | 11,410 | 263,293 | 10,974 | 255,220 | 10,198 |
| Moderate | 203,119 | 8,860 | 187,615 | 7,391 | 183,352 | 8,670 | 179,353 | 8,496 | 174,644 | 7,873 |
| Modifier | 54,284,887 | 8,360,143 | 52,059,222 | 8,267,651 | 49,019,496 | 9,022,248 | 45,203,505 | 8,561,167 | 46,150,176 | 8,290,112 |
| **Total number of effects** | 54,785,263 | 8,386,925 | 52,522,941 | 8,291,488 | 49,475,828 | 9,049,417 | 45,651,116 | 8,587,443 | 46,584,689 | 8,314,314 |

**Table S6 (cont.).** The summary of variants annotated by SnpEFF for 17 goat populations including the entire Saanen group and the Boer group.

|  | **British-Alpine** | | | **Anglo-Nubian** | | **Entire Saanen group** | | **Korean Saanen** | | **Australian Saanen** | |  |
| --- | --- | --- | --- | --- | --- | --- | --- | --- | --- | --- | --- | --- |
|  | | **SNP** | **INDEL** | **SNP** | **INDEL** | **SNP** | **INDEL** | **SNP** | **INDEL** | **SNP** | **INDEL** | |
| **Region** | |  |  |  |  |  |  |  |  |  |  | |
| Down stream | | 1,544,424 | 275,206 | 1,299,550 | 238,245 | 2,111,481 | 367,374 | 1,592,350 | 303,398 | 1,269,457 | 252,004 | |
| Exon | | 259,196 | 10,667 | 182,127 | 8,177 | 418,103 | 17,191 | 302,473 | 13,841 | 224,093 | 9,826 | |
| Gene | | - | 87 | - | 104 | - | 93 | - | 93 | - | 51 | |
| Intergenic | | 19,297,635 | 3,041,784 | 17,336,183 | 2,794,600 | 25,742,717 | 4,049,922 | 19,277,526 | 3,410,861 | 15,521,811 | 2,806,376 | |
| Intron | | 15,400,426 | 2,459,118 | 13,598,196 | 2,208,082 | 20,555,519 | 3,280,980 | 15,270,414 | 2,727,064 | 12,303,630 | 2,230,642 | |
| Splice site acceptor | | 358 | 173 | 297 | 177 | 593 | 280 | 432 | 201 | 350 | 197 | |
| Splice site donor | | 547 | 320 | 382 | 212 | 824 | 435 | 643 | 328 | 459 | 284 | |
| Splice site region | | 26,246 | 4,495 | 20,192 | 3,785 | 40,209 | 6,459 | 29,762 | 5,377 | 23,025 | 4,405 | |
| Transcript | | - | 142 | - | 144 | - | 227 | - | 182 | - | 174 | |
| Upstream | | 1,523,679 | 262,328 | 1,324,555 | 232,895 | 2,038,777 | 342,209 | 1,553,832 | 287,668 | 1,226,069 | 239,940 | |
| UTR 3' | | 220,593 | 43,282 | 182,696 | 37,716 | 314,560 | 60,005 | 235,126 | 49,857 | 181,271 | 40,550 | |
| UTR 5' | | 43,453 | 5,735 | 32,524 | 4,742 | 68,970 | 8,434 | 52,927 | 6,959 | 36,976 | 5,181 | |
| **Functional class** | |  |  |  |  |  |  |  |  |  |  | |
| Missenese | | 118,644 | 2,214 | 84,291 | 1,701 | 190,747 | 3,776 | 135,441 | 3,277 | 101,323 | 2,121 | |
| Nonsense | | 1,850 | 45 | 1,282 | 31 | 2,912 | 87 | 1,999 | 73 | 1,542 | 46 | |
| Silent | | 140,844 | 2,187 | 98,267 | 1,443 | 228,056 | 3,664 | 167,548 | 3,047 | 123,232 | 2,107 | |
| **Impact** | |  |  |  |  |  |  |  |  |  |  | |
| High | | 3,252 | 4,157 | 2,278 | 3,231 | 5,049 | 6,444 | 3,643 | 5,016 | 2,764 | 3,734 | |
| Low | | 170,997 | 6,764 | 121,165 | 5,303 | 274,478 | 10,290 | 202,230 | 8,557 | 149,255 | 6,599 | |
| Moderate | | 118,187 | 4,829 | 83,992 | 3,867 | 190,077 | 7,796 | 134,911 | 6,329 | 100,936 | 4,416 | |
| Modifier | | 38,024,121 | 6,087,587 | 33,769,267 | 5,516,478 | 50,822,149 | 8,109,079 | 37,974,701 | 6,785,927 | 30,534,186 | 5,574,881 | |
| **Total number of effects** | | 38,316,557 | 6,103,337 | 33,976,702 | 5,528,879 | 51,291,753 | 8,133,609 | 38,315,485 | 6,805,829 | 30,787,141 | 5,589,630 | |

**Table S6 (cont.).** The summary of variants annotated by SnpEFF for 17 goat populations including the entire Saanen group and the Boer group.

|  | **French Saanen** | | | **Swiss Saanen** | | **Entire Boer group** | | **Korean Boer** | | **Australian Boer** | |
| --- | --- | --- | --- | --- | --- | --- | --- | --- | --- | --- | --- |
|  | **SNP** | **INDEL** | **SNP** | | **INDEL** | **SNP** | **INDEL** | **SNP** | **INDEL** | **SNP** | **INDEL** |
| **Region** |  |  |  | |  |  |  |  |  |  |  |
| Down stream | 843,904 | 240,987 | 957,157 | | 240,171 | 1,774,588 | 287,287 | 1,105,902 | 237,687 | 1,196,953 | 213,863 |
| Exon | 164,090 | 9,923 | 187,415 | | 10,685 | 259,063 | 9,825 | 202,088 | 9,988 | 115,486 | 5,374 |
| Gene | - | 48 | - | | 30 | - | 104 | - | 52 | - | 106 |
| Intergenic | 9,906,284 | 2,880,256 | 11,623,414 | | 2,849,976 | 22,770,937 | 3,190,465 | 13,653,804 | 2,703,374 | 16,518,980 | 2,547,546 |
| Intron | 7,599,677 | 2,176,205 | 8,775,935 | | 2,162,150 | 18,209,874 | 2,605,015 | 10,691,833 | 2,134,142 | 12,633,230 | 1,980,620 |
| Splice site acceptor | 239 | 143 | 260 | | 119 | 430 | 203 | 300 | 140 | 233 | 138 |
| Splice site donor | 357 | 159 | 422 | | 222 | 536 | 308 | 451 | 232 | 320 | 202 |
| Splice site region | 15,640 | 4,890 | 17,934 | | 4,572 | 28,188 | 4,184 | 20,504 | 4,287 | 14,558 | 2,530 |
| Transcript | - | 205 | - | | 182 | - | 126 | - | 178 | - | 113 |
| Upstream | 831,530 | 240,452 | 944,531 | | 235,901 | 1,745,555 | 267,419 | 1,087,041 | 224,793 | 1,258,528 | 213,060 |
| UTR 3' | 112,677 | 40,943 | 129,426 | | 40,111 | 247,172 | 43,531 | 160,483 | 39,624 | 141,883 | 29,061 |
| UTR 5' | 29,658 | 5,984 | 34,615 | | 6,122 | 42,211 | 5,075 | 35,789 | 5,070 | 22,587 | 3,342 |
| **Functional class** |  |  |  | |  |  |  |  |  |  |  |
| Missenese | 72,459 | 2,460 | 83,443 | | 2,704 | 119,996 | 2,057 | 89,650 | 2,515 | 54,525 | 1,120 |
| Nonsense | 987 | 54 | 1,246 | | 58 | 1,878 | 48 | 1,308 | 56 | 924 | 20 |
| Silent | 91,856 | 2,098 | 103,958 | | 2,403 | 139,644 | 1,960 | 112,865 | 2,353 | 61,142 | 952 |
| **Impact** |  |  |  | |  |  |  |  |  |  |  |
| High | 1,931 | 3,430 | 2,371 | | 3,533 | 3,289 | 3,985 | 2,522 | 3,534 | 1,707 | 2,337 |
| Low | 110,033 | 7,106 | 125,140 | | 7,129 | 171,543 | 6,195 | 136,459 | 6,742 | 77,722 | 3,501 |
| Moderate | 72,137 | 4,614 | 83,029 | | 5,020 | 119,577 | 4,458 | 89,214 | 4,508 | 54,305 | 2,503 |
| Modifier | 19,319,955 | 5,585,045 | 22,460,569 | | 5,534,559 | 44,784,145 | 6,398,904 | 26,730,000 | 5,344,783 | 31,769,024 | 4,987,614 |
| **Total number of effects** | 19,504,056 | 5,600,195 | 22,671,109 | | 5,550,241 | 45,078,554 | 6,413,542 | 26,958,195 | 5,359,567 | 31,902,758 | 4,995,955 |

**Table S6 (cont.).** The summary of variants annotated by SnpEFF for 17 goat populations including the entire Saanen group and the Boer group.

|  | **Swiss Boer** | | **French Alpine** | |
| --- | --- | --- | --- | --- |
|  | **SNP** | **INDEL** | **SNP** | **INDEL** |
| **Region** |  |  |  |  |
| Down stream | 335,038 | 90,056 | 763,369 | 240,042 |
| Exon | 50,235 | 2,900 | 141,219 | 9,362 |
| Gene | - | 9 | - | 78 |
| Intergenic | 4,379,486 | 1,198,551 | 9,321,186 | 2,882,531 |
| Intron | 3,346,621 | 871,627 | 7,055,080 | 2,174,995 |
| Splice site acceptor | 69 | 42 | 223 | 134 |
| Splice site donor | 108 | 26 | 291 | 184 |
| Splice site region | 5,656 | 1,600 | 13,744 | 4,812 |
| Transcript | - | 63 | - | 233 |
| Upstream | 327,032 | 86,907 | 752,330 | 237,772 |
| UTR 3' | 44,429 | 15,442 | 99,563 | 40,905 |
| UTR 5' | 9,148 | 1,574 | 24,952 | 5,767 |
| **Functional class** |  |  |  |  |
| Missenese | 21,128 | 898 | 61,880 | 2,482 |
| Nonsense | 242 | 11 | 906 | 46 |
| Silent | 29,347 | 828 | 79,287 | 2,013 |
| **Impact** |  |  |  |  |
| High | 579 | 793 | 1,769 | 3,148 |
| Low | 35,605 | 2,471 | 95,181 | 6,912 |
| Moderate | 20,973 | 1,314 | 61,549 | 4,489 |
| Modifier | 8,440,665 | 2,264,219 | 18,013,458 | 5,582,266 |
| **Total number of effects** | 8,497,822 | 2,268,797 | 18,171,957 | 5,596,815 |

**Table S7.** The summary of nucleotide diversity, inbreeding coefficient, and linkage disequilibrium for 17 goat populations including the entire Saanen group and the Boer group.

| **Group** | **Sample**  **size** | **Average**  **Pi** | **S. d.**  **Pi** | **Average**  **F** | **Average**  **r^2^ (30Kb)^a^** | **Average**  **r^2^ (50Kb)^a^** | **Average**  **r^2^ (100Kb)^a^** | **Average**  **r^2^ (500Kb)^a^** | **S. d.**  **r^2^ (500Kb)** |
| --- | --- | --- | --- | --- | --- | --- | --- | --- | --- |
| ***Capra aegagrus*** | 17 | 0.001804 | 0.000768 | 0.06821 | 0.105888 | 0.087908 | 0.069419 | 0.053188 | 0.019117 |
| **Korean Indigenous Goat** | 29 | 0.001472 | 0.000666 | 0.01661 | 0.181847 | 0.161031 | 0.136517 | 0.088431 | 0.031691 |
| **Iranian Indigenous Goat** | 18 | 0.001998 | 0.000798 | 0.06229 | 0.105888 | 0.087908 | 0.069419 | 0.034229 | 0.014073 |
| **Moroccan Indigenous Goat** | 20 | 0.001859 | 0.000776 | 0.06143 | 0.098639 | 0.079364 | 0.058419 | 0.034156 | 0.019898 |
| **Korean crossbred** | 13 | 0.001908 | 0.000767 | -0.02956 | 0.135434 | 0.117113 | 0.097428 | 0.068539 | 0.021502 |
| **British-Alpine** | 5 | 0.001251 | 0.000717 | -0.00711 | 0.453781 | 0.422692 | 0.382538 | 0.288801 | 0.058836 |
| **Anglo-Nubian** | 5 | 0.001117 | 0.000707 | -0.0234 | 0.517422 | 0.487128 | 0.444872 | 0.327566 | 0.072255 |
| **Entire Saanen group** | 16 | 0.001783 | 0.000753 | 0.00207 | 0.146189 | 0.063634 | 0.025877 | 0.063634 | 0.025877 |
| ***Korean Saanen*** | *10* | *0.001733* | *0.000749* | *-0.02360* | *0.196753* | *0.171958* | *0.144126* | *0.100624* | *0.030683* |
| ***Australian Saanen*** | *2* | *0.001577* | *0.000873* | *0.10373* | *0.539091* | *0.510988* | *0.476832* | *0.415701* | *0.039995* |
| ***Swiss Saanen*** | *2* | *0.001582* | *0.00087* | *-0.08018* | *0.541022* | *0.508691* | *0.469981* | *0.412954* | *0.039178* |
| ***French Saanen*** | *2* | *0.001623* | *0.001623* | *0.05853* | *0.530090* | *0.496187* | *0.453971* | *0.386762* | *0.044388* |
| **Entire Boer group** | 11 | 0.001724 | 0.000743 | -0.04455 | 0.220800 | 0.091387 | 0.028591 | 0.091387 | 0.028591 |
| ***Korean Boer*** | *4* | *0.001554* | *0.000808* | *-0.35578* | *0.402885* | *0.364865* | *0.31941* | *0.243723* | *0.05062* |
| ***Australian Boer*** | *6* | *0.001727* | *0.000751* | *0.03870* | *0.233220* | *0.209031* | *0.181594* | *0.137452* | *0.030774* |
| ***Swiss Boer*** | *1* | *0.001655* | *0.001230* | *-* | *-* | *-* | *-* | *-* | *-* |
| **French Alpine** | 2 | 0.001575 | 0.000861 | -0.08174 | 0.54274 | 0.506752 | 0.461822 | 0.389765 | 0.047156 |

^a^It means that the average value up to Kb specified in parentheses.

**Table S8.** The estimate of the mean of Fst value ​​for all pairs of 14 goat populations (Swiss Boer with sample size of 1 is excluded).

|  | **Korean  Indigenous** | **Korean crossbred** | **Moroccan Indigenous** | **Iranian Indigenous** | ***Capra aegagrus*** | **Anglo Nubian** | **British Alpine** | **French Alpine** | **French Saanen** | **Australian Saanen** | **Swiss Saanen** | **Korean Saanen** | **Australian Boer** | **Korean Boer** |
| --- | --- | --- | --- | --- | --- | --- | --- | --- | --- | --- | --- | --- | --- | --- |
| **Korean Indigenous** | - | 0.072116 (0.034435) | 0.10594 (0.037287) | 0.081081 (0.030869) | 0.130252 (0.038777) | 0.180935 (0.071141) | 0.188479 (0.072551) | 0.162052 (0.09002) | 0.160939 (0.090326) | 0.154618 (0.089916) | 0.159331 (0.090244) | 0.145953 (0.054123) | 0.147619 (0.059849) | 0.184183 (0.075346) |
| **Korean crossbred** | 0.072116 (0.034435) | - | 0.05114 (0.025225) | 0.026289 (0.016285) | 0.085427 (0.028496) | 0.104803 (0.050268) | 0.108326 (0.050105) | 0.054877 (0.055996) | 0.054096 (0.057111) | 0.04792 (0.058317) | 0.052784 (0.057982) | 0.07327 (0.035298) | 0.056769 (0.035713) | 0.092315 (0.045609) |
| **Moroccan Indigenous** | 0.10594 (0.037287) | 0.05114 (0.025225) | - | 0.029849 (0.01739) | 0.082482 (0.029423) | 0.103233 (0.048684) | 0.088452 (0.046878) | 0.019896 (0.056576) | 0.02119 (0.055816) | 0.020659 (0.056) | 0.02008 (0.056417) | 0.064699 (0.032184) | 0.055098 (0.035899) | 0.076914 (0.045432) |
| **Iranian Indigenous** | 0.081081 (0.030869) | 0.026289 (0.016285) | 0.029849 (0.01739) | - | 0.065148 (0.025283) | 0.071324 (0.039076) | 0.071824 (0.039627) | 0.002476 (0.046662) | 0.002234 (0.046723) | 0.000514 (0.047345) | 0.002752 (0.047706) | 0.055151 (0.027865) | 0.029875 (0.026701) | 0.049476 (0.034657) |
| ***Capra aegagrus*** | 0.130252 (0.038777) | 0.085427 (0.028496) | 0.082482 (0.029423) | 0.065148 (0.025283) | - | 0.13767 (0.049328) | 0.129555 (0.048399) | 0.075587 (0.059244) | 0.074534 (0.058639) | 0.073666 (0.058691) | 0.07229 (0.058496) | 0.102406 (0.035521) | 0.104164 (0.039617) | 0.130862 (0.048105) |
| **Anglo Nubian** | 0.180935 (0.071141) | 0.104803 (0.050268) | 0.103233 (0.048684) | 0.071324 (0.039076) | 0.13767 (0.049328) | - | 0.276981 (0.140582) | 0.290073 (0.151319) | 0.291374 (0.153561) | 0.285594 (0.160631) | 0.284124 (0.153414) | 0.161364 (0.073983) | 0.172107 (0.081933) | 0.293844 (0.118664) |
| **British Alpine** | 0.188479 (0.072551) | 0.108326 (0.050105) | 0.088452 (0.046878) | 0.071824 (0.039627) | 0.129555 (0.048399) | 0.276981 (0.140582) | - | 0.19021 (0.148277) | 0.193237 (0.146862) | 0.171668 (0.152181) | 0.181669 (0.145191) | 0.085526 (0.065056) | 0.161883 (0.07969) | 0.265983 (0.112486) |
| **French Alpine** | 0.162052 (0.09002) | 0.054877 (0.055996) | 0.019896 (0.056576) | 0.002476 (0.046662) | 0.075587 (0.059244) | 0.290073 (0.151319) | 0.19021 (0.148277) | - | 0.099725 (0.14294) | 0.097522 (0.156395) | 0.059899 (0.148992) | 0.045982 (0.072242) | 0.105195 (0.085646) | 0.250663 (0.109702) |
| **French Saanen** | 0.160939 (0.090326) | 0.054096 (0.057111) | 0.02119 (0.055816) | 0.002234 (0.046723) | 0.074534 (0.058639) | 0.291374 (0.153561) | 0.193237 (0.146862) | 0.099725 (0.14294) | - | 0.09511 (0.149122) | 0.06238 (0.147581) | 0.038747 (0.070733) | 0.105267 (0.082729) | 0.252326 (0.109712) |
| **Australian Saanen** | 0.154618 (0.089916) | 0.04792 (0.058317) | 0.020659 (0.056) | 0.000514 (0.047345) | 0.073666 (0.058691) | 0.285594 (0.160631) | 0.171668 (0.152181) | 0.097522 (0.156395) | 0.09511 (0.149122) | - | 0.061285 (0.159936) | 0.008463 (0.067902) | 0.100496 (0.08693) | 0.247324 (0.12101) |
| **Swiss Saanen** | 0.159331 (0.090244) | 0.052784 (0.057982) | 0.02008 (0.056417) | 0.002752 (0.047706) | 0.07229 (0.058496) | 0.284124 (0.153414) | 0.181669 (0.145191) | 0.059899 (0.148992) | 0.06238 (0.147581) | 0.061285 (0.159936) | - | 0.036739 (0.071206) | 0.101853 (0.086795) | 0.243521 (0.116675) |
| **Korean Saanen** | 0.145953 (0.054123) | 0.07327 (0.035298) | 0.064699 (0.032184) | 0.055151 (0.027865) | 0.102406 (0.035521) | 0.161364 (0.073983) | 0.085526 (0.065056) | 0.045982 (0.072242) | 0.038747 (0.070733) | 0.008463 (0.067902) | 0.036739 (0.071206) | - | 0.10465 (0.051077) | 0.151756 (0.064988) |
| **Australian Boer** | 0.147619 (0.059849) | 0.056769 (0.035713) | 0.055098 (0.035899) | 0.029875 (0.026701) | 0.104164 (0.039617) | 0.172107 (0.081933) | 0.161883 (0.07969) | 0.105195 (0.085646) | 0.105267 (0.082729) | 0.100496 (0.08693) | 0.101853 (0.086795) | 0.10465 (0.051077) | - | 0.060494 (0.062231) |
| **Korean Boer** | 0.184183 (0.075346) | 0.092315 (0.045609) | 0.076914 (0.045432) | 0.049476 (0.034657) | 0.130862 (0.048105) | 0.293844 (0.118664) | 0.265983 (0.112486) | 0.250663 (0.109702) | 0.252326 (0.109712) | 0.247324 (0.12101) | 0.243521 (0.116675) | 0.151756 (0.064988) | 0.060494 (0.062231) | - |

*The standard deviation of the estimated Fst value is indicated in parentheses

**Table S9.** The estimate of effective population size (Ne) for 14 goat populations (Swiss Boer with sample size of 1 is excluded).

| **Years (before the present)** | ***Capra aegagrus*** | **Iranian**  **Indigenous goat** | **Moroccan**  **Indigenous goat** | **Korean**  **Indigenous goat** | **Anglo**  **Nubian** |
| --- | --- | --- | --- | --- | --- |
| **1** | 446 | 18,487 | 12,917 | 1,671 | 140 |
| **47** | 260 | 13,113 | 31,433 | 1,016 | 224 |
| **121** | 671 | 22,072 | 46,703 | 331 | 107 |
| **236** | 251 | 80,255 | 92,882 | 680 | 143 |
| **417** | 520 | 62,646 | 40,156 | 1,820 | 1,326 |
| **700** | 5,662 | 54,241 | 90,076 | 5,931 | 2,320 |
| **1,143** | 33,743 | 118,824 | 22,508 | 8,948 | 11,304 |
| **1,836** | 152,666 | 79,949 | 56,004 | 37,597 | 23,619 |
| **2,921** | 99,589 | 86,600 | 19,597 | 30,509 | 76,690 |
| **4,619** | 135,020 | 78,939 | 100,915 | 44,521 | 110,064 |
| **7,277** | 40,477 | 81,186 | 153,488 | 71,001 | 161,300 |
| **11,438** | 27,123 | 16,550 | 200,290 | 62,290 | 84,555 |
| **17,951** | 79,629 | 33,732 | 28,073 | 79,288 | 86,019 |
| **28,147** | 68,521 | 33,201 | 35,820 | 67,284 | 90,428 |
| **44,107** | 52,229 | 92,834 | 35,382 | 71,176 | 58,807 |
| **69,089** | 34,449 | 111,139 | 88,869 | 50,906 | 63,423 |
| **108,194** | 45,268 | 58,155 | 58,289 | 66,330 | 73,943 |
| **169,408** | 96,546 | 52,215 | 47,779 | 89,223 | 85,792 |
| **265,227** | 101,709 | 79,761 | 78,642 | 36,544 | 37,682 |
| **415,216** | 29,299 | 107,750 | 112,523 | 38,530 | 35,536 |
| **650,000** | 64,243 | 19,551 | 13,983 | 71,928 | 7,762 |

**Table S9 (cont.).** The estimate of effective population size (Ne) for 14 goat populations (Swiss Boer with sample size of 1 is excluded).

| **Years (before the present)** | **French Alpine** | **French Saanen** | **Swiss Saanen** | **Australian Saanen** | **Korean Saanen** |
| --- | --- | --- | --- | --- | --- |
| **1** | 3,274 | 5,557 | 36,157 | 344 | 231 |
| **47** | 8,125 | 2,858 | 53,552 | 895 | 814 |
| **121** | 8,058 | 1,570 | 49,210 | 4,968 | 1,541 |
| **236** | 7,403 | 1,793 | 22,198 | 11,089 | 4,520 |
| **417** | 12,353 | 1,851 | 26,606 | 5,696 | 11,285 |
| **700** | 16,223 | 2,025 | 87,111 | 19,015 | 4,117 |
| **1,143** | 3,181 | 8,252 | 61,686 | 25,013 | 3,499 |
| **1,836** | 11,404 | 15,913 | 59,310 | 34,232 | 1,917 |
| **2,921** | 79,658 | 17,737 | 69,939 | 10,582 | 3,114 |
| **4,619** | 49,502 | 41,101 | 15,376 | 30,628 | 26,985 |
| **7,277** | 34,877 | 63,931 | 18,667 | 50,992 | 38,481 |
| **11,438** | 28,685 | 24,743 | 16,023 | 36,422 | 50,450 |
| **17,951** | 18,995 | 51,562 | 12,884 | 47,061 | 68,696 |
| **28,147** | 27,866 | 65,576 | 27,632 | 58,977 | 114,788 |
| **44,107** | 33,245 | 92,001 | 25,439 | 46,913 | 78,149 |
| **69,089** | 65,311 | 38,339 | 37,523 | 77,565 | 65,889 |
| **108,194** | 70,474 | 86,544 | 28,129 | 61,373 | 103,970 |
| **169,408** | 69,746 | 44,016 | 40,427 | 121,469 | 24,613 |
| **265,227** | 93,549 | 88,381 | 99,161 | 49,337 | 29,516 |
| **415,216** | 101,158 | 90,479 | 85,666 | 39,329 | 63,467 |
| **650,000** | 34,242 | 36,142 | 87,584 | 44,065 | 61,888 |

**Table S9 (cont.).** The estimate of effective population size (Ne) for 14 goat populations (Swiss Boer with sample size of 1 is excluded).

| **Years (before the present)** | **Australian Boer** | **Korean Boer** | **British-Alpine** | **Korean crossbred** |
| --- | --- | --- | --- | --- |
| **1** | 166 | 3,548 | 120 | 149 |
| **47** | 268 | 2,989 | 128 | 1,636 |
| **121** | 1,014 | 1,321 | 166 | 2,009 |
| **236** | 457 | 655 | 728 | 38,669 |
| **417** | 933 | 129 | 4,038 | 34,318 |
| **700** | 6,840 | 1,190 | 5,310 | 28,349 |
| **1,143** | 40,604 | 11,885 | 19,624 | 42,323 |
| **1,836** | 77,270 | 2,540 | 58,105 | 121,355 |
| **2,921** | 138,331 | 17,521 | 48,803 | 60,641 |
| **4,619** | 89,737 | 42,409 | 33,161 | 119,142 |
| **7,277** | 99,514 | 41,723 | 20,770 | 76,604 |
| **11,438** | 67,894 | 251,503 | 13,736 | 140,477 |
| **17,951** | 82,735 | 208,855 | 22,631 | 107,609 |
| **28,147** | 66,350 | 217,200 | 28,554 | 87,020 |
| **44,107** | 52,913 | 131,970 | 39,538 | 110,493 |
| **69,089** | 77,029 | 159,829 | 40,992 | 108,794 |
| **108,194** | 83,548 | 115,769 | 49,207 | 104,027 |
| **169,408** | 53,269 | 33,783 | 97,687 | 55,061 |
| **265,227** | 113,676 | 42,187 | 81,767 | 73,742 |
| **415,216** | 43,673 | 86,825 | 60,065 | 99,813 |
| **650,000** | 137,707 | 110,056 | 47,690 | 31,498 |

**Table S10. The number of selected genes corresponding to the top 0.1% regions in the XP-CLR and XP-EHH analysis results.**

| **Selected groups** | **The number of Genes** | | | |
| --- | --- | --- | --- | --- |
|  | **XP-CLR** | **XP-EHH** | **Union** | **Intersection** |
| ***Capra aegagrus*** | 341 | 574 | 801 | 114 |
| **Iranian Indigenous goat** | 301 | 748 | 893 | 156 |
| **Moroccan Indigenous goat** | 311 | 776 | 930 | 157 |
| **Korean crossbred** | 343 | 369 | 605 | 107 |
| **Korean Boer** | 255 | 1199 | 1232 | 152 |
| **Korean Saanen** | 363 | 383 | 628 | 115 |
| **Entire Boer group** | 338 | 466 | 673 | 133 |
| **Entire Saanen group** | 320 | 374 | 584 | 109 |
| **British-Alpine** | 339 | 435 | 629 | 145 |
| **Anglo-Nubian** | 394 | 518 | 751 | 161 |

**S5: Supplementary Excel Tables, provided as additional files 2-6**

**5.1 Additional File 2**

The results of D-statistic and three-population tests in Korean indigenous goat and Korean crossbred.

**5.2 Additional File 3**

The results of XP-EHH analysis for each of the 10 goat populations including the entire Saanen group and the Boer group.

**5.3 Additional File 4**

The results of XP-CLR analysis for each of the 10 goat populations including the entire Saanen group and the Boer group.

**5.4 Additional File 5**

The summary of Gene-set enrichment analysis for each of the 10 goat populations including the entire Saanen group and the Boer group.

**5.5 Additional File 6**

The summary of six non-synonymous variants and their surrounding haplotype frequencies found in four candidate selected genes.
